# AntiSplodge: a neural-network-based RNA-profile deconvolution pipeline designed for spatial transcriptomics

**DOI:** 10.1093/nargab/lqac073

**Published:** 2022-10-10

**Authors:** Jesper B Lund, Eric L Lindberg, Henrike Maatz, Fabian Pottbaecker, Norbert Hübner, Christoph Lippert

**Affiliations:** Digital Health & Machine Learning Research Group, Hasso Plattner Institut for Digital Engineering, Potsdam, Germany; Cardiovascular and Metabolic Sciences, Max Delbrück Center for Molecular Medicine in the Helmholtz Association (MDC), Berlin, Germany; Cardiovascular and Metabolic Sciences, Max Delbrück Center for Molecular Medicine in the Helmholtz Association (MDC), Berlin, Germany; DZHK (German Centre for Cardiovascular Research), Partner Site Berlin, Berlin, Germany; Digital Health & Machine Learning Research Group, Hasso Plattner Institut for Digital Engineering, Potsdam, Germany; Cardiovascular and Metabolic Sciences, Max Delbrück Center for Molecular Medicine in the Helmholtz Association (MDC), Berlin, Germany; DZHK (German Centre for Cardiovascular Research), Partner Site Berlin, Berlin, Germany; Charite, Universitätsmedizin Berlin, Berlin, Germany; Digital Health & Machine Learning Research Group, Hasso Plattner Institut for Digital Engineering, Potsdam, Germany; Hasso Plattner Institute for Digital Health at Mount Sinai, Icahn School of Medicine at Mount Sinai, New York, NY, USA

## Abstract

With the current surge of spatial transcriptomics (ST) studies, researchers are exploring the deep interactive cell-play directly in tissues, in situ. However, with the current technologies, measurements consist of mRNA transcript profiles of mixed origin. Recently, applications have been proposed to tackle the deconvolution process, to gain knowledge about which cell types (SC) are found within. This is usually done by incorporating metrics from single-cell (SC) RNA, from similar tissues. Yet, most existing tools are cumbersome, and we found them hard to integrate and properly utilize. Therefore, we present *AntiSplodge*, a simple feed-forward neural-network-based pipeline designed to effective deconvolute ST profiles by utilizing synthetic ST profiles derived from real-life SC datasets. *AntiSplodge* is designed to be easy, fast and intuitive while still being lightweight. To demonstrate *AntiSplodge*, we deconvolute the human heart and verify correctness across time points. We further deconvolute the mouse brain, where spot patterns correctly follow that of the underlying tissue. In particular, for the hippocampus from where the cells originate. Furthermore, *AntiSplodge* demonstrates top of the line performance when compared to current state-of-the-art tools. Software availability: https://github.com/HealthML/AntiSplodge/.

## INTRODUCTION

Recently, technologies capturing the *in-situ* diversities of the transcriptomic landscape have emerged (1–3). These have spawned a subfield of transcriptomics coined spatial transcriptomics (ST), also known as spatially resolved transcriptomics(4,5). ST allows for localized measurements of RNA content, mapped directly in the interrogated tissues. The most popular and highest throughput ST technologies such as the 10X Visium (1) and slide-seq (2,6) technologies, relies on spots (probe capture areas), rather than on fluorescent imaging, which still today remains lower throughput (3,7).

The advantages of these new technologies are manyfold. We can now perform inquires directly in the tissue of interest to gain previously unseen knowledge about what genes are up-regulated within the visible regions interrogated. However, the single-cellular populations remains a mystery in the spot-based approaches and thus does the corresponding cell-cell communication, cell function, and cell states kept within, which accounts for organ functionality, disease development, and pathology. The spot-based ST techniques and their corresponding RNA profiles(1) are obtained from populations of cells that are mixed, including mixed cell-states, as multiple cells can contribute to the RNA profiles generated.

With the recent vast improvements within SC transcriptomics (8), including highly detailed, explored, and phenotyped datasets (9), multi-modality projects spanning both fields have been enabled (10,11). As a result, researchers have already shown proof of concepts for tackling the deconvolution of the spatial transcriptomics profiles (12–17), these includes, *SPOTlight* (14), *Stereoscope* (12), *Cell2Location* (17) and *DSTG* (18).

*SPOTlight* constructs topic profiles based on a selected reference scRNA dataset, in a way such that each cell type has a uniquely assigned topic profile, i.e. each topic profile accounts for a specific cell type’s expression profile. This is done using seeded non-negative matrix factorization (NMF) regression, initialized using cell type marker genes, which can subsequently be used to deconvolute spatial transcriptomics spots using a non-negative least squares (NNLS) method.

*Stereoscope* uses a model-based approach to estimating both the cell type signatures and the relative cell abundance for each spot. This is done by estimating coefficients and rates, which resembles logits for each gene, to measure how much a gene’s expression contributes to a specific cell type proportion based on a scRNA reference dataset. These estimates are found through a training procedure, with assumptions that the data follows a negative binomial distribution. When the logits for all genes are estimated, a profile can be deconvoluted based on these logits by examining the log counts of each gene, where logits are summarized and scaled to form a proportion vector. One major issue with *Stereoscope* is its run-time-complexity, in our comparison it took more than 24 hours to train, others also report long training times, for example, *Cell2Location* reported >14 h for training for *Stereoscope* in their tests.

*Cell2Location* is a Bayesian model, which estimates absolute cell densities for each cell type by decomposing mRNA counts of each gene, i.e. cell type proportions can be estimated by scaling the resulting cell type count profiles to 1. *Cell2Location* comes with several analysis procedures, where the standard procedure for estimating cell types are a statistical method based on Negative Binomial regression, much like the *Stereoscope* method, where logits are estimated for each gene. One issue with *Cell2location* is that it is heavyweight in terms of the number of required dependencies, which can be non-trivial to implement into an existing pipeline.

*DSTG* which is short for Deconvoluting Spatial Transcriptomics data through Graph-based convolutional networks and is a tool implemented in Python using Tensorflow (19). It relies on constructing a graph based on the input data, where edges are generated in a preprocessing step and passed into the neural network, which is then used to train the convolutional filters of the network. *DSTG* uses single-cell data to construct synthetic ST spot data, and then computes approximate profiles by linking these real spot data in the graph, using a nearest neighbours approach. We had a few issues running *DSTG*, including having to alter the source code of the preprocessing step to exclude genes with no nearest neighbours (and therefore no connection) in the graphs (as our data had these, see the Comparison section), but it was fast once it was running.

Despite these existing applications, highly accurate and precise deconvolution of spatial transcriptomics spots remains a challenge, and we found that on average these tools produced profiles deviating from the ground truth profiles in the range of 10–25% (see Comparison). Therefore, we deemed it adequate to contribute with *AntiSplodge*, a neural-network-based tool, for fast and accurate deconvolution of spot-based RNA profiles.

The main strength of *AntiSplodge* comes from its ease of usage, speed, and accuracy. With the annotation of synthetic ST profiles using cell type information originating from real-life SC datasets, we further eliminate the data imbalance that usually exists in biological datasets. Additionally, because of the accompanying temperature sampling method, it is possible to generate millions of profiles in short amounts of time (usually minutes). The sampler captures both extreme and mean cell abundances, and uses the specific cell profiles which allow for learning the biases within each cell type, which further enhances the accuracy of the networks trained.

The utility of *AntiSplodge* comes from its high speed, a typical analysis is in the range of a few hours, where training usually take between 15 minutes to 90 minutes while requiring no more RAM than a standard laptop. *AntiSplodge* can be run with less than 16GB of RAM needed, and training the model required less than two GB of GPU memory (alternatively, this can be done on the CPU with an expected increase in training time). Only a few libraries outside the standard Python libraries are required, making it easy to integrate into existing environments and pipelines, and the whole pipeline has been wrapped into a python package (https://pypi.org/project/antisplodge/). The tool is presented with a comparison to existing state-of-the-art software, and a showcase of the various workflows enabled by the software.

## MATERIALS AND METHODS

### Cell density

Cell density is the measure of the number of contributing cells in a single RNA profile. In spatial transcriptomics, it is the measure of cells per spot, where each spot has a single RNA profile. As noted by recent publications (20), the average cell per spot is technology dependant, but also tissue-dependent. It is deemed reasonable that for the 10× genomics’ Visium Spatial Gene Expression, the cell density lies in the range of 1–10, whereas for the more custom technologies such as the Slide-seqV1 (2), a cell density of 10–40 is oftentimes observed. In the developing human heart study (20), they observed an average cell density of 20–40 by manual inspection of the tissue images. *AntiSplodge* requires a cell density range to be specified by the user.

### Marker genes

Prior to training the *AntiSplodge* model, it is recommended to reduce the number of genes of the profiles, to a set of genes that are heterogeneous among the cells of the SC dataset. This allows for saving memory, as we need to generate many synthetic profiles (usually in the range of hundreds of thousands to millions), each removed gene essentially saves a lot of memory. Additionally, this increases training speed, as we need to look at fewer genes, and therefore computations will be performed faster, but it also increases the effectiveness of the algorithm, as we only look at non-redundant genes, and thus, converge faster. Overall, the precision penalty of removing redundant genes will have little to no impact on the resulting deconvolutions.

#### Finding marker genes

In order to find marker genes, we use the following data-driven procedure. We find the intersecting gene set among all STs to be deconvoluted and the SC dataset. Using this new gene set, we find the marker genes by performing logistic regression for each gene, note that, logistic regression is more time consuming than performing *t*-tests, which can also be used. The fact that we are using a large number of genes diminishes the difference between the t-tests and logistic regressions. For smaller sets of marker genes, logistic regression is generally preferred (21). The marker genes models are computed using Scanpy (22). This choice of model is recommended by previous work (23). These tests are carried out on a gene basis by comparing one cell type to the rest of the cell types in the SC dataset.

By doing so, we get a score for each gene, which we can use to rank our genes for each cell type. We then select the top X genes for each cell type, which might contain duplicates across cell types, we simply use the unique set of these (scanpy ranks genes by *Z*-score). As a safety check, we assess the power of the resulting gene set using a gradient boosting trees model (XGBClassifier from scikit-learn (24)). To do this, we split the SC into 90% train and 10% test and train the classifier, with the SC profiles as input and their corresponding cell type as the outcome. If the test accuracy is at least 90%, then we deem this gene set a good differentiable gene set for the cell types. If the test accuracy is below 90% we recommend seeking other means to compute the marker genes. Note: We do not need the statistics from these tests, we simply need to know what genes to include when we produce the synthetic ST profiles.

### Sampling synthetic ST profiles

In order to generate synthetic ST profiles, based on a reference SC dataset, we sample scRNA profiles from multiple cell types. To ensure that we extract both the extreme (all cells of one type) and mean (mixed cell types) cell type distributions, we use a multinomial distribution, defined as:

$$\begin{equation*} f(x_1,...,x_K;p_1,...,p_K) = \frac{\Gamma {(\sum _ix_i+1)}}{\prod _i\Gamma {x_i+1}}\displaystyle \prod ^K_{i=1}{p_i}^{x_i} \end{equation*}$$

where *p*_*i*_ denotes the chance of drawing cell type *i*, *K* is the number of classes (in this case cell types), and $\sum _{i=1}^Kp_i=1$. Here, *x*_*i*_ is the number of sampled types for cell type *i*. The weights for the multinomial draws (*p*), are further sampled using a temperature function defined as:

$$\begin{equation*} p_i = ({CD}^{i})^{\left(\frac{temp}{steps}\right)} \end{equation*}$$

where *i* ∈ [0, *K*], and *K* is the number of classes. Here, *temp* ∈ [0, *steps*], and *steps* is the number of temperature steps that are used to sample weights. Each *p* are scaled, so that they in total sum to 1 by diving $\sum _{i=1}^K p_i$. Intuitively, the temperature function starts with equal weights (all equal to 1), and scales to weights that have *pow* times higher for each increasing *i*. This allows us to sample the extreme RNA profile cases, where the cells all originate from the same type (*temp* = *steps*), as well as, profiles that are fully mixed (*temp* = 0). For each profile sampled, the produced weights of the temperature function are shuffled to randomize which cell type get which power, thus ensuring that all cell types can sample extreme profiles when the temperature (*temp*) goes to 1.

Once a cell type distribution for a given profile has been found, the cells corresponding to the number of each cell type (*x*) are extracted from the SC dataset and combined into a single profile, where the sum of the gene counts of the profile are scaled to sum to 1. See Supplementary Section 1 for a more detailed explanation of the sampling method.

### The AntiSplodge model

#### Architecture

The neural network is a feed-forward network with decreasing nodes for each hidden layer block, before the output layer with nodes equal to the number of cell types in the SC dataset. The true power of the method comes from how the sampling is performed. The input is a vector of gene counts (profile), corresponding to the count for each marker gene, scaled in the range of between 1 and 0, but usually very close to 0 because of the high number of genes. The output is a vector containing the predicted estimate of the cell type proportion. Three groups of hidden layers are used. With the default settings, these start as a pair of hidden layers with size 512, followed by pairs of layers with sizes 256 and 128, leading to a last single hidden layer with size 64. The last layer outputs the predicted proportions. Between each group of layers (hidden pairs, and the last layer), ReLu activation units are used, while for each hidden pair, a batch normalization process is used to make training faster and more stable. For *AntiSplodge* the default loss is a L1 loss. The loss is computed before the output scaling is applied, as we found this to optimize the weights to not output zero sum vectors (as zero sum vectors are usually a problem that occurs with a large number of cell types). This was further enforced by having the connection between the last hidden layer and the output layer, linked by a smooth ReLu function with a 0.1 gradient, punishing the loss score for vectors containing negative values. Network topology is that of a standard multilayer perceptron.

#### scRNA dataset split during training

As with most machine-learning methods, to assess the power of the model, generated synthetic profiles are divided into a train, validation, and test sets. The training set is used to train the model, usually, 90% or 95% of the profiles are in this set. We leave the testing profiles for accuracy estimation for when the training is complete. We use the validation set to select the best model parameters during training. These will be saved to a checkpoint file during training, and once the training is complete we will load the best parameters found back onto the model (based on the error of the validation dataset).

During training, the network will see each copy of the synthetic training dataset (synthetic RNA profiles) in mini-batches, while at each finished epoch, we check how the current weights compare on the validation dataset. For each lower validation loss, we save the current weights of the model. This is to counter the overfitting of the training data.

#### Output scaling

As a proportion vector always sums to 1, any vector with a higher or lower sum is simply not a good estimate. Therefore, at the end of the model, linear scaling is applied, after a smooth ReLU activator:

$$\begin{equation*} X_i = \frac{x_i}{S},\ \ \ S = \sum _i X_i \end{equation*}$$

where *x*_*i*_ is the *i*th element of the vector *X*. And *S* is the initial sum of the *X* vector. Note, the error is computed beforehand to train the network to output elements between 0 and 1, as this worked better for problems with a higher number of cell types.

#### Hyperparameters

*AntiSplodge* has been implemented in Python using PyTorch (25). By default, is uses the Adam (26) optimizer with a L1 Loss (MAE). The number of genes to use in the synthetic profiles is found by trial and error, we recommend starting with profiles of around 1000 unique genes. You might need to increase this number if the Jensen-Shannon Divergence (JSD) reported is not satisfactory. JSD is a distance metric used to measure the similarity between two probability distributions, and it is the symmetric and smoothed version of the Kullback–Leibler divergence. JSD is often used for comparison of cell-count profiles in the literature (12,14,17,18). If the JSD reported is already satisfactory, it is possible to decrease the number of genes used in order to save computations. The number of profiles needed is heavily dependent on the homology of the cell types to deconvolute. We recommend starting with a manageable amount of profiles, depending on your system, and increasing this if the results do not look promising.

## RESULTS

### The AntiSplodge pipeline

**Figure 1.**
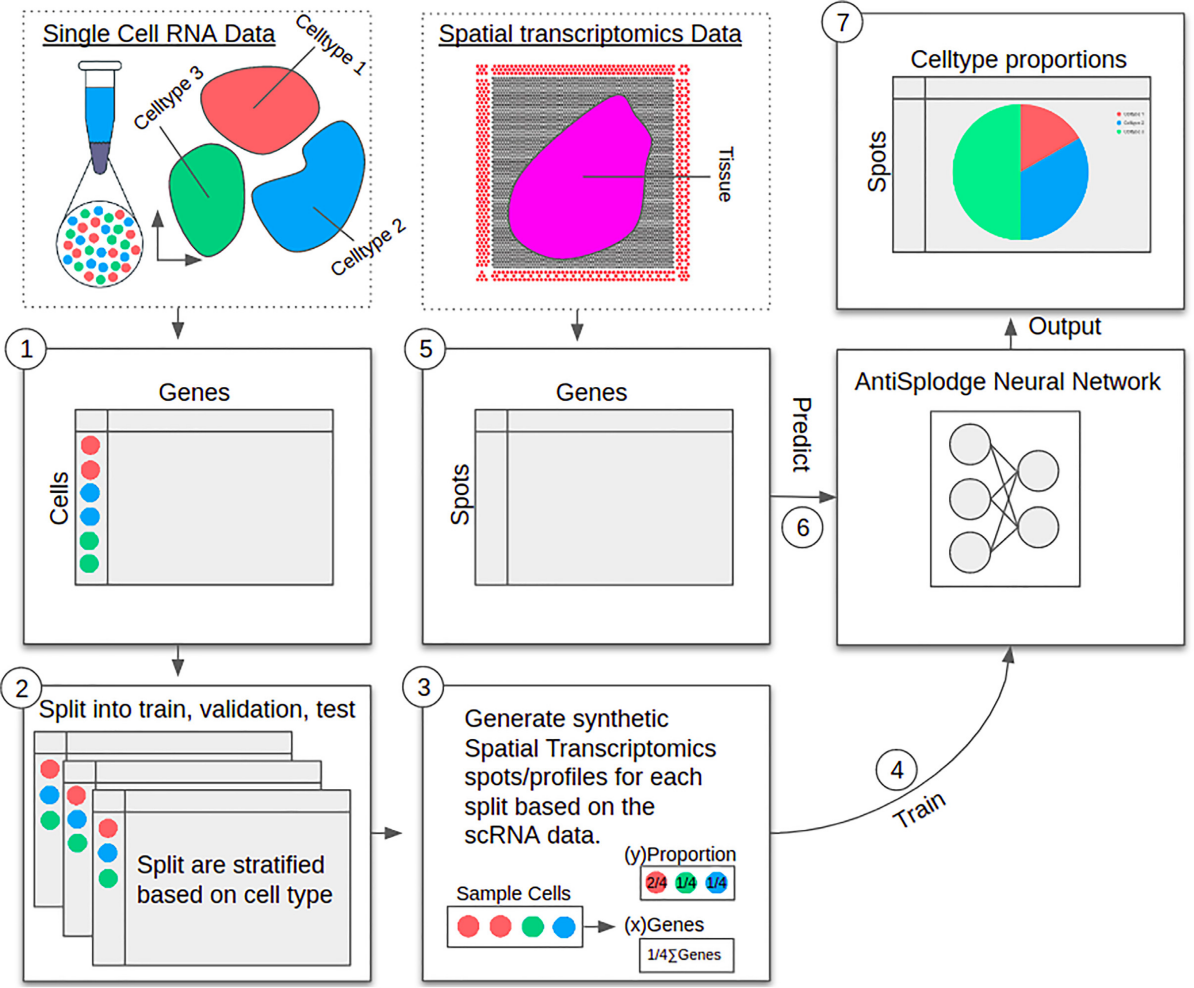
Overview of the AntiSplodge pipeline. Dotted boxes are outside the AntiSplodge regime, while full drawn boxes are the steps of AntiSplodge. (1) First the scRNA data along with cell type information are added. (2) The data is then split into three chunks, stratified by cell type information, to make train, validation, and test dataset splits. (3) For each of the dataset splits, synthetic spot profiles are generated based on the cells from the scRNA data. (4) The AntiSplodge neural network is initialized and trained using the synthetic profiles, where the ground truth is known. (5) Once the network has been trained, the spatial transcriptomics data is loaded. (6) With the trained network, the spot profiles are passed through the network to deconvolute each profile. (7) The output of the network is a proportion vector indicating the percentage of each cell type for each spot profile, these can then be used in downstream analysis tasks.

The *AntiSplodge* pipeline is presented in Figure 1. You only need two datasets to use *AntiSplodge*, an SC and an ST dataset (dotted boxes in Figure 1). Preferably, these dataset comes from the same individuals, but they could also originate from the same type of tissue (e.g. heart tissue), as long as they are comparable (e.g. individuals with similar phenotypes, for the example of heart tissues, both datasets could be from individuals with no heart diseases).

For the SC part of the pipeline, first, the chosen dataset (Figure 1(1)) is split stratified based on cell types into train, validation, and test splits (Figure 1(2)). This ensures that there is an equal proportion of each cell type in each split. Then synthetic profiles are sampled for each dataset split (Figure 1(3)). The synthetic profile uses the mean of each gene count for all cells selected. The user needs to decide the number of potential cells that contribute to each profile. See the Methods section for more details.

Finally, the network can be trained with the generated profiles (Figure 1(4)), where the user can freely decide what optimizer and error function to use, along with additional training parameters to control how long and how the training will be. We recommend using *L1 Loss*, as *L1 Loss* does a good job of reducing over-fitting in combination with using the validation dataset (see Supplementary Section 2).

For the ST part of the pipeline, all that is needed is to pass the spot profiles to the *AntiSplodge* neural-network (Figure 1(5 and 6)), and the network will output the predicted cell type proportions, which will be a percentage of each cell type, where each profile sums to 1 (100%).

The true strength of this approach comes from the synthetic profiles, as they will be closely comparable (given that the SC and ST datasets are comparable) to real-life spot profiles. We highly recommend doing data quality control and performing normalization techniques on both the SC and ST datasets, especially keeping profiles of each set on the same scale, for example, scale both sets to profiles with total gene counts of 1.

### Comparison

In order to validate our tool, we compared our methods to three state-of-the-art methods, namely; *SPOTlight*, *Stereoscope*, *Cell2Location* and *DSTG*. Furthermore, we made a baseline model, to check the complexity of the problem, and additionally, a regression-based Random Forest model was trained to check the utility of a traditional machine learning model. The models were tested to see how well they could deconvolute synthetic RNA profiles where we have the ground truth, compared in JSD. This is done by sampling SCs from the preprocessed Heart Cell Atlas (HCA), further referred to as synthetic ST spots.

#### Dataset and quality control

We downloaded the full catalogue of scRNA profiles from HCA (9), which comprises 486 134 SC and single-nuclei profiles, containing a total of 33 538 genes. We refer to both as cells. For preprocessing during our comparison, we removed cells with unassigned cell types or cells which were predicted as doublets. Furthermore, we performed strict quality control; removing cells with more than 1% mitochondrial genes, and the top and bottom 2.5% based on total gene count to get a more consistent set of cells, gene count-wise. Subsequently, we removed genes that were no longer found in any cell. After preprocessing a total of 305,875 cells remained, containing a total of 31 504 genes, distributed across 11 cell types. Using top 150 genes for each cell type, gave us a total of 1389 unique genes. The test accuracy for predicting each cell type using the procedure described above is 99.04% with a 90%/10% train/test split.

#### Comparison setup

We sampled a total of 2 100 000 training synthetic ST spot profiles (100 000 profiles for each number of cell densities used). For the validation and test dataset, we sampled 42 000 synthetic ST spots, 2000 of each cell density. The synthetic validation ST spots are only used during training of *AntiSplodge*. We here have an unscaled version (competing methods) and scaled version (our method and baselines) of the dataset.

In this comparison, each of the tools (*SPOTlight*, *Stereoscope*, *Cell2Location*, and *DSTG*) were trained on the training dataset, accordingly to their documentations. For *AntiSplodge* and the *Random Forest* model, we generated synthetic ST spots using normalized gene-count profiles (each profile sums to 1) based on the training dataset. The profiles were generated with cell densities in the range of 10–30 (both inclusive). Initially, we divided the SC samples into a 80% train, 10% validation, and 10% test datasets.

#### Hyperparameters for each model of the comparison

In order to reproduce our results, we here present the hyperparameters used for the models of the comparison. The *baseline* model predicts an equal proportion for each cell type defined by $\frac{1}{N_C}$ where *N*_*C*_ is the total number of classes, i.e. an equal proportion of all classes or cell types (C). In this comparison, *N*_*C*_ is 11. The *RandomForest* model is built using scikit-learn’s (27) implementation of Random Forest Regressor. It was trained using the synthetic train profiles and tested using the synthetic test profiles (parameters: n_estimators = 1000, max_depth = 20, min_samples_split = 0.05, min_samples_leaf = 0.05, max_features = ‘sqrt’).

*SPOTlight*, *Stereoscope*, *Cell2Location* and *DSTG*, was trained using the default parameters recommended by in their respective tutorials, using the unscaled gene-count matrices (the training split of the scRNA dataset). *AntiSplodge* was trained on the training dataset, with the validation dataset used to find the best loss, and tested on the test dataset (all synthetic).

#### Results

**Figure 2.**
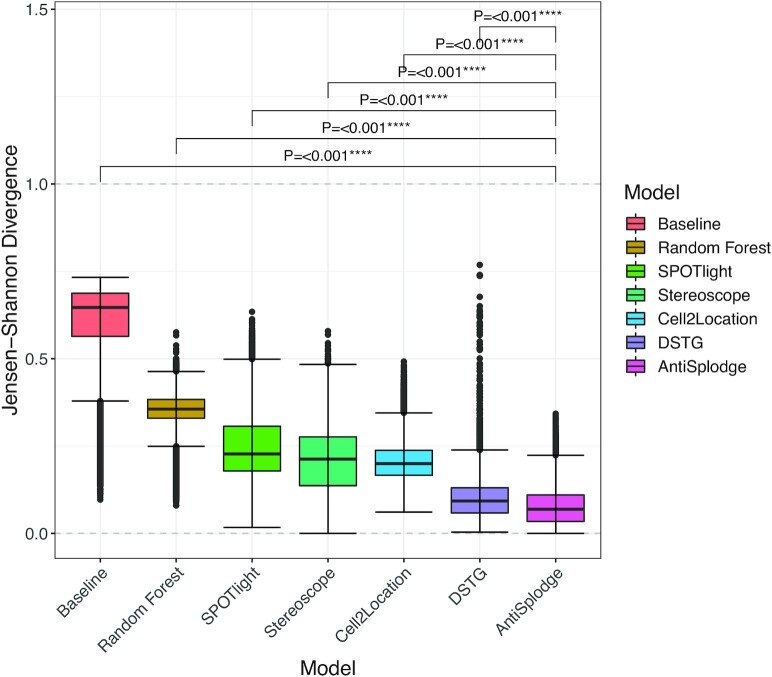
Results for performance compared across the models tested, based on synthetic data generated from single-cell data from the Heart Cell Atlas, measured by Jensen–Shannon divergence (JSD). Ordered by their mean JSD, from left to right: *Baseline* (mean: 60.5%), Regression-based *Random Forest* (mean: 35.1%), *SPOTlight* (mean: 24.8%) *Stereoscope* (mean: 20.6%), *Cell2Location* (mean: 20.6%), *DSTG* (mean: 10.0%), and our method *AntiSplodge* (mean: 7.6%). *P*-values at the top of the plot are Bonferroni adjusted *t*-test *P*-values, comparing the distributions of JSDs for each model against *AntiSplodge*.

The results for predicting the test profiles are presented in Figure 2. Note, all models output profiles that sum to 1, either through scaling (see Materials and Methods section) or by natural behaviour of the algorithms.

The *Baseline* model performs the worst with a mean JSD of 60.5%. Random Forest had a relatively high mean JSD of 35.1%, compared to the methods designed for deconvolution (both competing and our methods). Each of the competing models produced quite similar proportion profiles in terms of mean JSD (*SPOTlight*: 24.8%, *Stereoscope*: 20.6%, and *Cell2Location*: 20.6%). However, we want to emphasize here that we used the tools with the default settings (with only minor alterations to make some of them run). *DSTG* has a very comparable mean JSD to that of *AntiSplodge* with 10.0%. *AntiSplodge* had the lowest mean JSD of all the tools compared (7.6%), which is considerably lower than the competing methods. Cell population overview and marker genes can be found in Supplementary Section 3. Additionally, we computed Bonferroni adjusted *t*-test *P*-values for the output of *AntiSplodge* compared to each of the other models, in order to see if there was a significant difference between the distributions of the outputs. Here we found that there was a significant difference between the output of *AntiSplodge* and all the other models (these are shown at the top of Figure 2).

#### 10-fold cross-validation

**Figure 3.**
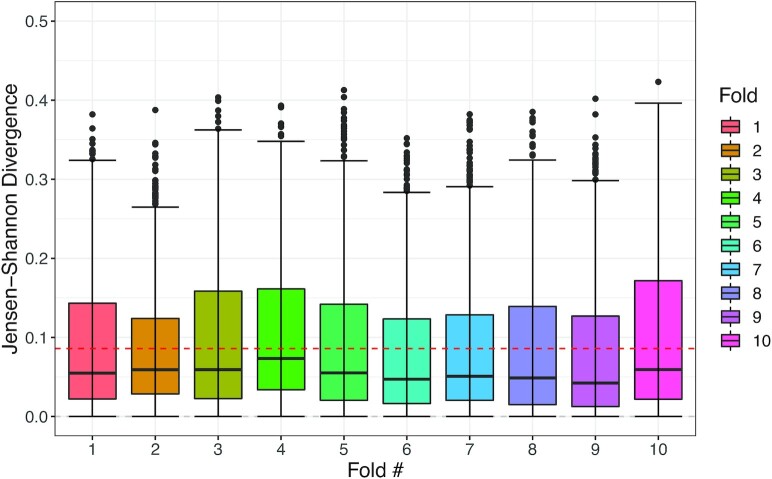
Results for the *AntiSplodge* 10-fold cross-validation. Each fold contains 2500 synthetic test profiles with a cell density of 10. From left to right, we have a mean JSD of: Fold 1: 8.64% (SD: 7.97%), Fold 2: 8.27% (SD: 7.11%), Fold 3: 9.18% (SD: 8.43%), Fold 4: 10.05% (SD: 8.07%), Fold 5: 8.67% (SD: 8.28%), Fold 6: 7.78% (SD: 7.74%), Fold 7: 8.11% (SD: 7.83%), Fold 8: 8.16% (SD: 8.14%), Fold 9: 7.72% (SD: 8.15%), Fold 10: 9.37% (SD: 8.53%). Red line denotes the mean JSD across all folds with 8.59%.

In order to test the robustness of *AntiSplodge*, we performed 10-fold cross-validation. Using the same dataset (HCA (9)), we filtered it to only contain samples that were gathered from Harvard Nucleic sequencing (*N* = 163 959), again removing doublets and cell types there were defined as not assigned and filtering genes such that we only had the marker genes found in the comparison (*N* = 1389), we did not do any trimming of the dataset. We then constructed 10-fold, where each fold was stratified to contain an equal number of samples for each cell type. For each fold, we set up an *AntiSplodge* experiment, where 9-fold was used for training and the remaining were used for testing. For each experiment, we constructed 100 000 training, 2500 validation and 2500 testing profiles, synthetically, each with a cell density of 10. We then initialized a model for each experiment and trained each with 25 warm restarts (where the best found weight configuration were loaded back onto the model) with restarts occurring when we did not see an improvement for five epochs. The total run time of all experiments time was 2 h and 23 min. The mean JSDs reported were from the lowest 7.71% mean JSD to the highest 10.05% mean JSD, the total mean across all 10-fold was 8.59% JSD. The folds have been plotted in Figure 3. Overall, this demonstrates that *AntiSplodge* is very robust, even with random initiation of the weights. We used the Adam (26) optimizer with an L1 Loss (MAE). A Python notebook with the full reproducible analysis is found in the GitHub repository. Note, even though 10-fold cross-validation is normally used to select the best model, we mainly use it to show the robustness of the model across multiple initiations, which produces very similar results. One could of course select any of the folds (usually the one with the best accuracy) for the down-steam analysis.

### Application 1: Deconvoluting the developing human heart

#### Overview

Recently, an organ-wide cell atlas of the developing human heart has been published (20). The dataset features 19 slices of fetal hearts, from three different time points in the first trimester: 4.5–5, 6.5 and 9 post-conception weeks (PCW, *N* = 4, 9 and 6, respectively), with ST measurements. Additionally, paired SC gene expression data, featuring 3717 SCs with an average of 2900 genes per cell, is available. The SCs are annotated with cell types, covering 14 classes, with some of the cell types sharing the same majority type.

We intersect all 19 ST samples with the paired SC dataset to a common gene set (*N* = 7638). Using the SC dataset, we compute the marker genes via logistic regression, as explained during the comparison. We found that using the top 200 genes for each cell type (1387 unique genes) yielded a 94.35% test accuracy, which makes the gene set a good gene set for the cell type differentiation.

#### AntiSplodge pipeline

First we divided the SC dataset into a a 90% train, 5% validation and 5% test split. We then created synthesized ST profiles, with cell densities in the range of 20–41 (which is noted in the ST dataset manuscript (20) as the average cell counts found for each spot). For each cell density we create 10 000 train, 2000 validation and 2000 test synthesized profiles, for a total of 210 000 train, 42 000 validation and 42 000 test profiles.

Training the model took 13.83 min on a single GPU, and it ran for 192 epochs (with the last 100 being the patience epochs with no increase in validation performance), the best model was found at epoch 93. We achieve a mean Jensen–Shannon divergence (JSD) of 12.91% in the test dataset.

#### Results

**Figure 4.**
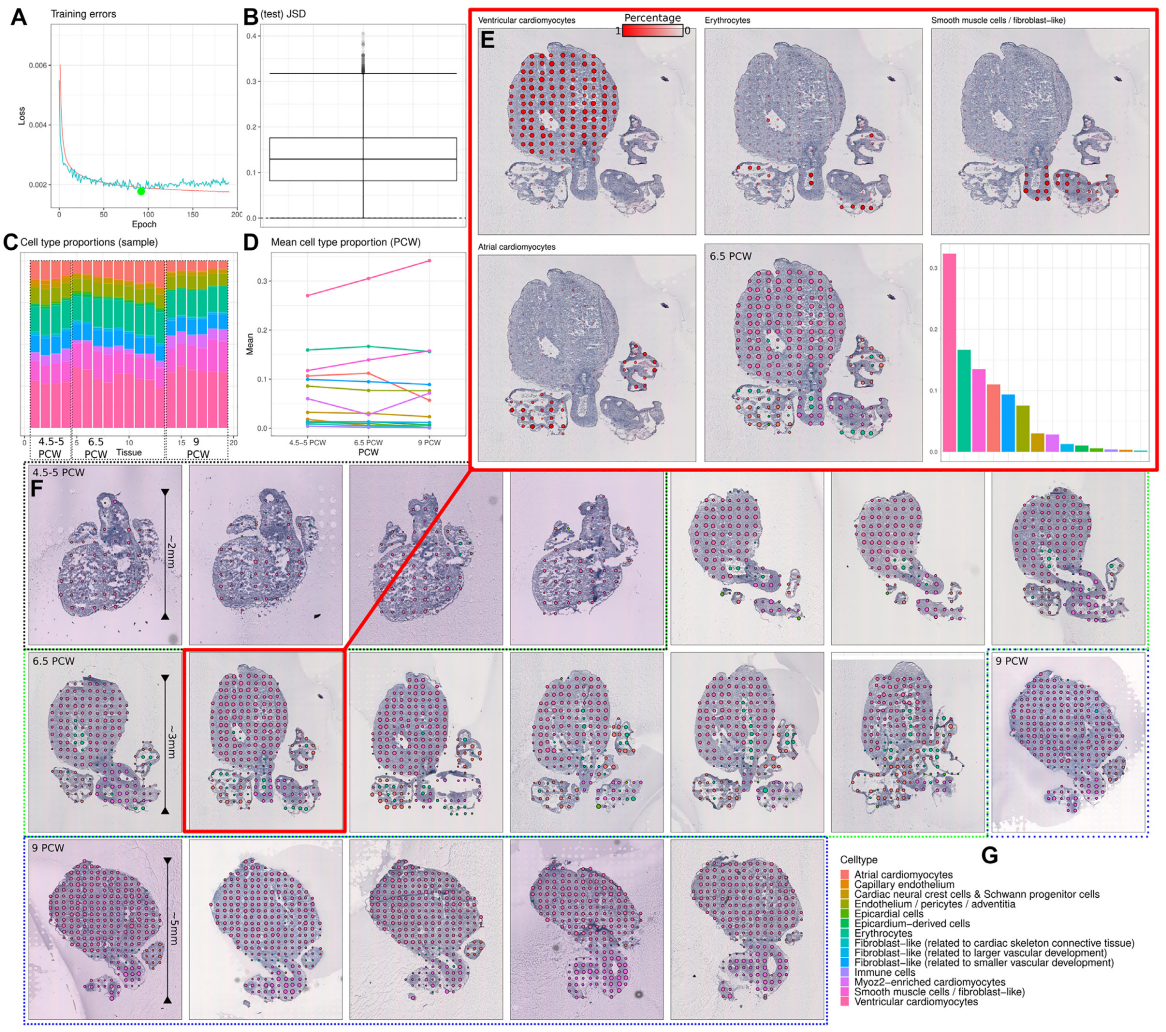
Results for ST samples from the developing human heart (*N* = 19). (**A**) Loss over epochs, best loss found is indicated with a green dot (epoch = 92, loss = 0.0017). (**B**) Jensen–Shannon divergence box plot for the test samples (mean JSD = 12.9%). (**C**) Distributions of cell types across samples, ordered by PCW. (**D**) Mean proportion of each cell type for each PCW. (**E**) Zoom of the fourth 6.5 PCW sample. First four plots are the top four cell types ordered by their cell type abundance across ST spots (highest first), ventricular cardiomyocytes, erythrocytes, smooth muscle cells/fibroblast-like, and atrial cardiomyocytes. Fifth plot is the corresponding plot in (F). Last plot is the cell type distribution across spots. (**F**) Map of maximum cell types for each spot for each of the samples. Samples are ordered as; first four is 4.5–5 PCW (black), next nine is 6.5 PCW (green), and last six is 9 PCW (purple). (**G**) Legend for cell types and their associated colors.

The results for this application has been displayed in Figure 4. With the fast training of the network (∼14 min, Figure 4A), we see a very satisfactory low mean JSD (12.91%, Figure 4B). Across all samples, we see a coherent pattern of the highest cell types abundances residing in the regions to which they are expected to be found (Figure 4F). Here, ventricular cardiomyocytes are found in the regions of the left and right ventricle, while atrial cardiomyocytes are found in the regions of the left and right atrium. Smooth muscle and fibroblast-like cells are found in the smooth muscle tissues connecting the heart muscles. Erythrocytes are found in spots with low-density tissues such as heart chambers. Furthermore, looking at Figure 4E, we see that these cell types are only predicted in the regions where we would expect, but have little to no contributions in the other regions. If we look at how the cell type distributions change over time (i.e. PCW, Figure 4C and D), we see a more or less stable abundancy relation between the cell types except for ventricular cardiomyocytes, which seems to increase over time (based on their proportions allocated in the spots), which is most likely since the ventricular regions are growing/developing over this period, which can be seen with the naked eye in Figure 4F. Cell population overview, marker genes, and cell type proportions per sample, can be found in Supplementary Section 4.

### Application 2: Deconvoluting the mouse brain

#### Overview

With the release of the 10× genomics visium spatial technology, several demonstration datasets were publicly released. These included datasets for the mouse brain, which we deconvolute in this application. For this, we use five datasets, namely the adult mouse brain sample (coronal view), and the four sagittal cuts of the mouse brain (two for the posterior and two for the anterior part of the mouse brain) (28). To deconvolute these samples, we downloaded scRNA profiles (*N* = 76 533, with 45 768 unique genes) from the Allen mouse brain atlas, which are SCs isolated from >20 areas of mouse cortex and hippocamopus (29), processed with a 10× SMART-seq machine. In this dataset, there are four major cell types, and 42 subclass cell types, with a nomenclature for the subcell types of either describing what part of the brain they reside (e.g. CA1, which is a region of the mouse hippocampus) or what genes are highly expressed (e.g. *Vip* and *Meis2*). In this application, we want to deconvolute the mouse brain using the 42 subclass cell types.

We find the intersecting gene set among all STs and the SC dataset (*N* genes=15 262). We compute the marker genes, as explained in the Comparison using t-tests and use the top 250 genes (3559 unique genes), which yield a 97.73% test accuracy. This makes this gene set a good set for differentiation among the 42 cell types. We used *t*-tests as the number of cell types and therefore the number of unique genes, were too high for the logistic regression to be beneficial, and thus we could save computational time.

#### AntiSplodge pipeline

We create synthesized ST profiles, with cell densities in the range of 1–20 (note the 10× samples usually have between 1 and 10 cells (20), but we add another 10 to serve as a buffer for the upper limit). For each cell density we create 30 000 train, 5000 validation and 5000 test synthesized profiles, for a total of 600 000 train, 100 000 validation and 100 000 test profiles. We used a 90% train, 5% validation and 5% test split, for the SC samples that were used to make the synthetic ST profiles.

Training the model took 96.24 min on a single GPU, and it ran for 490 epochs, the best model was found at epoch 389. We achieve a mean Jensen–Shannon divergence (JSD) of 12.12% in the test dataset.

#### Results

**Figure 5.**
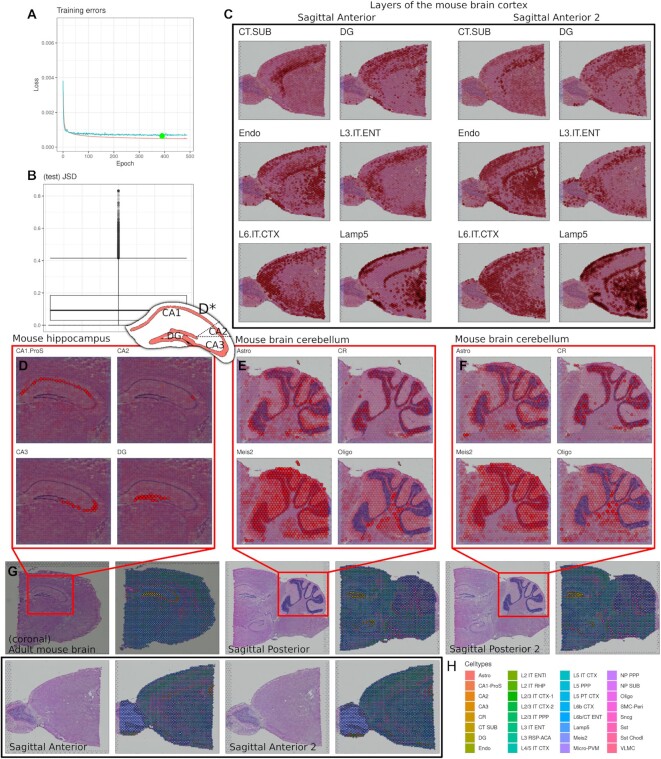
Results for ST samples from the developing human heart (*N* = 5). (**A**) Loss over epochs, best loss found is indicated with a green dot (epoch = 390, loss = 0.0006). (**B**) Jensen-Shannon Divergence box plot for the test samples (mean JSD = 12.12%). (**C**) Predicted cell types for layers of the mouse brain cortex in the sagittal anterior samples, different types are found in the different cortex layers. (**D**) Predicted distributions of cell types for CA1, CA2, CA3 (cornu ammonis) and DG (dentate gyrus). Note: In the Allen mouse brain atlas, the cell subclass types are defined by the regions in which they were found. (D*) Hippocampus regions according to the Allen mouse brain atlas. (**E**) Predicted cell types in the mouse brain for the first sagittal posterior sample. (**F**) Predicted cell types in the mouse brain for the second sagittal posterior sample. (**G**) Pairs of the tissue and deconvoluted spots, for the mouse brain ST samples. (**H**) Legend for cell types and their associated colors, for the figures in (G).

The results for this application has been displayed in Figure 5. Based on the network performance, we see a very satisfactory low mean JSD (12.12%, Figure 5B), even though the complexity of the problem is quite high, with 42 subcell types to estimate. The perhaps most intriguing result is shown in Figure 5D, which is a zoom-in of the mouse hippocampus. Here, we see that the cell types; CA1, CA2, CA3 (cornu ammonis), and DG (dentate gyrus) are extremely well predicted based on their associated brain region found in Figure 5D*. These cells are named after those particular regions in the Allen mouse brain atlas. For each of the five tissues and their respective spot predictions (Figure 5G), we see that the cell type majority (indicated by color), closely follows the tissue regions. For both the mouse cerebellum (Figure 5E and F) and brain cortex (Figure 5C), we see that the cell type expressions are heavily dependent on the regions of the brain. Furthermore, for both brain structures, we see coherent and matching cell types expression across the samples, which demonstrates the reliability of the network. Cell population overview, marker genes and cell type proportions per sample, can be found in Supplementary Section 5.

## DISCUSSION AND CONCLUSION

In this study, we present a completely new method for the process of deconvoluting spot areas of spatial transcriptomics by utilizing SC profiles from similar or matched tissues. Although the deconvolution idea is no longer novel, we think that the speed and accuracy of *AntiSplodge* could be of interest to the science community. We show that this procedure generates approximations far more accurate (or less diverged) compared to state-of-the-art methods which estimate profiles mainly based on the contributions of logits computed for each gene. In addition, by using the included sampler, researchers no longer need to take care of skewed distributions, as this is taken care of directly by the sampling procedure.

In our study, we illuminated the workflow of the pipeline with two applications. One is the deconvolution of the developing human heart, and the other is the adult mouse brain. For the developing human heart, we showed that the deconvoluting process, correctly estimated cell types based on where they are expected to frequently appear. This was in particular apparent for cells of the type; ventricular cardiomyocytes, smooth muscle cells, and atrial cardiomyocytes (Figure 4E and F). These patterns are further confirmed when we compare our results to a recent study preprint (30), especially for the two oldest sets of the tissues (6.5 and 9 PCW). However, in the same preprint, they did not find the same patterns of the expected cells types in 4.5–5 PCW samples, while we did in our study. Although the patterns are less sharply expressed (at least in our study), it seems that their method failed to fully deconvolute the cell type signals in the youngest of samples (4.5–5 PCW), perhaps because the profiles seems to be more differentially convoluted, compared to the older samples.

In the mouse brain applications, we similarly found credible patterns of cell types which based on the literature seems highly plausible. For example, in the mouse hippocampus (Figure 5D and D*), we found that the cell types CA1, CA2, CA3 (cornu ammonis), and DG (dentate gyrus) exactly followed the patterns of the regions (31–33) (from which they are named in the Allen mouse brain atlas, which is the SC dataset used). Furthermore, the expressions found in the mouse brain cortex (Figure 5C), followed similar patterns found in recent deconvolution publications which used the same ST dataset (14,34). In addition, by manual inspection, most cell types were distributed in patterns that closely followed the underlying tissue, which is broadly used as a confirmation of results in experimental ST publications.

As with most of the available deconvolution tools, we recommend using a smaller set of marker genes, as such a set yields faster training while reducing the memory footprint, in our case considerably if a large number of synthetic profiles are generated. While like most deconvolution tools, it is still feasible to run with the full gene set. Although in theory, more genes mean more heterogeneous cell type profiles, the extra noise added (combined with reduced performance) is usually not worth the effort. One additional advantage of using marker genes is that when stricter quality control and filtering is applied to both the SC and ST dataset, using marker genes found in the trimmed data ensures a higher orchestration of intersectional genes, which makes it easier to ensure that cell types markers are present across both data sets.

In conclusion, we have shown that *AntiSplodge* is a proven contender among state-of-the-art ST deconvolution tools and that it is capable of producing good results within very short time spans, both for problems with low and high complexity. One general problem persists for deconvolution of ST spots using SC data, unlike the developing human heart study (20) which contains SC and ST data from the same donor with adjacent tissues, most ST datasets do not contain SC data (and *vice-versa*), and determining if an SC dataset is a good representative for the cells in the convoluted ST spots, remains unsolved. However, this might be temporarily fixed by adding good uncertainty estimates to the results (35), which is something we are currently investigating and hope to share some results on in a near future.

## DATA AVAILABILITY

The software is available at: https://github.com/HealthML/AntiSplodge/. The single cell profiles from the Heart Cell Atlas can be found at: https://www.heartcellatlas.org/. The developing human heart spatial transcriptomics samples and along with the paired scRNA can be found at: https://www.spatialresearch.org/resources-published-datasets/doi-10-1016-j-cell-2019-11-025/. The 10X mouse brain spatial transcriptomics are located at: https://support.10xgenomics.com/spatial-gene-expression/datasets/. The mouse brain scRNA profiles from the Allen mouse brain atlas are found at: https://portal.brain-map.org/atlases-and-data/rnaseq/mouse-whole-cortex-and-hippocampus-smart-seq.

## Supplementary Material

lqac073_Supplemental_File
