# Supplementary material for: AntiSplodge: a neural-network-based RNA-profile deconvolution pipeline designed for spatial transcriptomics
Source: NAR Genom Bioinform. 2022 Oct 10;4(4):lqac073. doi: 10.1093/nargab/lqac073 (PMC9549785; doi:10.1093/nargab/lqac073)
Supplement: lqac073_Supplemental_File [file lqac073_supplemental_file.pdf]

Supplementary files for:

AntiSplodge: A neural network-based RNA-profile deconvolution pipeline. Designed for spatial transcriptomics.

Jesper B. Lund[1], Eric L. Lindberg[2], Henrike Maatz[2,3], Fabian Pottbaecker[1], Norbert Hübner[2,3,4], and, Christoph Lippert[1,5,*]

July 7, 2022

*[1]Digital Health & Machine Learning Research Group, Hasso Plattner Institut for Digital Engineering, Potsdam, Germany [2]Cardiovascular and Metabolic Sciences, Max Delbrück Center for Molecular Medicine in the Helmholtz Association (MDC), Berlin, Germany. [3]DZHK (German Centre for Cardiovascular Research), Partner Site Berlin, Berlin, Germany. [4]Charite, Universitätsmedizin Berlin, Berlin, Germany. [5]Hasso Plattner Institute for Digital Health at Mount Sinai, Icahn School of Medicine at Mount Sinai, New York, NY, USA. *Corresponding author, e-mail: Christoph.Lippert@hpi.de

Supplementary Section 1: Sampling properties

The main strength of *AntiSplodge* comes from the fact that we sample new synthetic spatial transcriptomics profiles from real scRNA cells. First, theoretical profiles are sampled, examples of these are shown in Supplementary Figure 2. Next, these profiles are converted to synthetic spatial transcriptomics profiles by picking a random number of cell types equal to the count in the profile, where cell type index corresponds to the respective index in the profile. The sampler will start by having an equal chance of each cell type (index), and then go towards the more extreme profiles, where some indices will have higher probabilities of being selected. These probabilities are shuffled for each profile generated, so that which cell type has the highest probability is random for each profile.

	0	1
**0**	1	1
**1**	1	1
**2**	2	0
**3**	1	1
**4**	1	1
**...**	...	...
**995**	2	0
**996**	2	0
**997**	1	1
**998**	0	2
**999**	0	2

1000 rows × 2 columns

Supplementary Figure 1: Theoretical profiles based on Supplementary Figure 2 (a) [Nc=2, M=1000, CD_min=2, CD_max=2]. Each column represents a cell type, and each row represents a profile.

An example of the profiles generated in Supplementary Figure 2 (a), is shown in Supplementary Figure 1. In this particular example, we have two cell types (Nc=2), and each profile has a cell density of 2 (the number of cell that are used to generate the profile CD_min, CD_max = 2). A total of 1000 profiles is generated (M=1000). If we for example had [M=1000, CD_min=1, CD_max=2] we would generate 1000 profiles with CD=1, and 1000 profiles with CD=2, totalling the number of profiles to 2000.

If we look at the profiles generated (in Supplementary Figure 1), we see that profile 1 (index 0) has 1 cell of cell type 0 and 1 cell of cell type 1. This is true for profile 1, 2, 4, 5. For profile 3, we see that cell type 0 has 2 cell for this profile, this is due to the fact that the probability selected cell type 0 two times (remember in the beginning each type has equal probabilities, so this is possible). If we look at the last 5 profiles (indices: 995, 996, 997, 998, 999), we see that 995, 996, 998, and 999 has 2 of the same cell types (because we are now sampling with a higher bias towards one cell), but it is still possible to sample equal profiles like in profile index: 997.

If we turn our attention to the distributions in Supplementary Figure 2. In **(a)** we see how the sampler does a good job of distributing cell types evenly across the possible profiles that it can generate. We know that in this case, whenever 1 cell type has been contributed with 1 in a profile, the other must be contributed with a 1 to satisfy the CD of 2. There is a total of 1000 profiles being generated and approximately 50% (500) has a 1 for each cell type. The remaining profiles will have a 2 in one of them and 0 in the rest (to satisfy CD=2). Each has approximately 25% of 2's, meaning a total of 50% 2, which leaves 50% of 0's, meaning we have equal distribution of all possible distributions of profiles.

In **(c)** and **(d)** we extended the problem to 4 cell types (with M=2000). In **(c)** all profiles have CD of 4, while in **(d)** we have 2000 profiles of CD 1, CD 2, CD 3, CD 4. Like in **(a)** we the distributions are actually equal, but now

shared across 4 cell types. In **(c)**, whenever we see a profile with 4 of one cell type, all the (three) others must now have a 0 in their counts. Whenever one cell type has 3, one of the others must have a 1 and the remaining 2 must have a 0. While for profiles with a 2, one of the others can have a 2, or two of the others can each have a 1, with the remaining having a 0. This gives this ladder distribution which can might not seem equal, but it is. For **d** we see the same distributions with more profiles in the lower counts, as a result of the lower CDs.

In **(e)** and **(f)** we see the generation with CD = 10, but for **(e)** we generate 1000 profiles and **(f)** we generate 100000 profiles. Here the overall distributions stays the same regardless of the number of profiles generated, which is what we would like to see.

In **(b)** we extend the number of cell types to 9 (with CD=10 and M=100000) which is a more realistic case. Again we see that the distributions are the same across cell types, but with higher concentration of 0s, as a 9 in one type will generate 8 zeroes, one in each of the rest of the cell types. A 8 will generate a 1 in one of the others and zero in the rest of the 7, etc.

For all of the distributions, each cell type is represented an equal number of times (Supplementary Figure 3), regardless of their proportionality in the scRNA dataset that will be used. If we look at Supplementary Figure 3**(a)**, we see that each cell type has approximately 1000 cell counts, 1006 and 994, for 0 and 1 ,respectively. The total number of cell counts can be computed with the formular:

$$\sum_{i=CD\_min}^{CD\_max} (i) \cdot M,$$

while the counts per cell type is then approximately divided among the cell types:

$$\frac{\sum_{i=CD\_min}^{CD\_max} (i) \cdot M}{N_c}.$$

For example, if we look at Supplementary Figure 3**(c)** and **(d)**. For **(c)**, we have:

$$4 \cdot 2000 = 8000$$

which is then divided between the four cell types so that each has approximately 2000 counts. While for **(d)**, we have:

$$10 \cdot 2000 = 20000$$

which is then divided between the four cell types so that each has approximately 5000 counts.

Conclusion: The distributions grasp all the possible cases of counts, which is in particular nice when working with neural networks in an unbiased way. We recommend using a sufficient high M but with a CD equal (both min and max the same) to the suspected number of CDs in the STs being deconvoluted. If you suspect that many of the spots will have a tendency towards a lower CD but with a few high CDs we recommend generating profiles in a range from the low suspected number of CDs towards the expected high CD. For example in the range of [1,2,3,4,5,6,7,8,9,10].

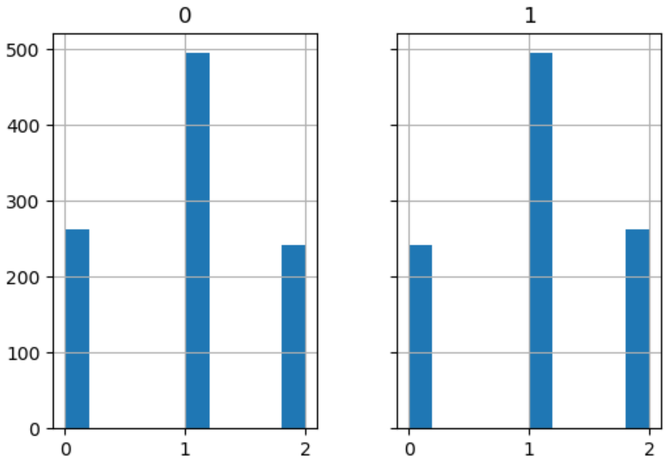

(a) **[Nc=2, M=1000, CD_min=2, CD_max=2]**

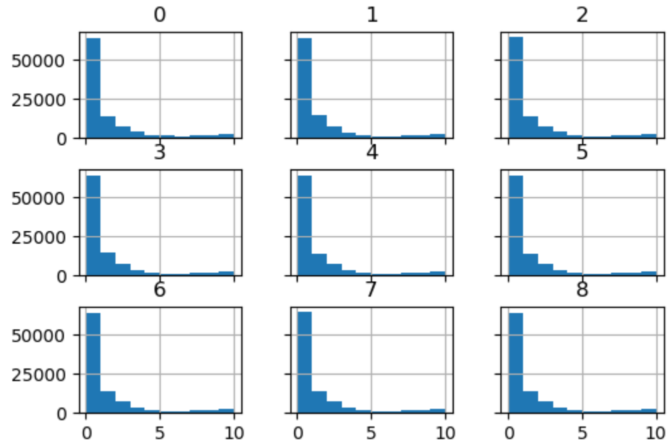

(b) **[Nc=9, M=100000, CD_min=10, CD_max=10]**

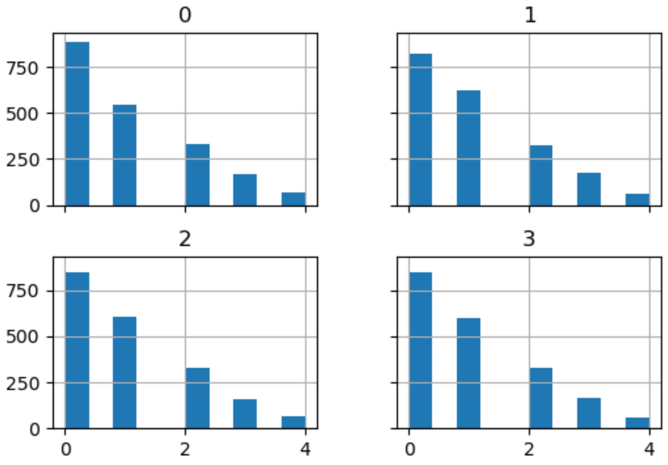

(c) **[Nc=4, M=2000, CD_min=4, CD_max=4]**

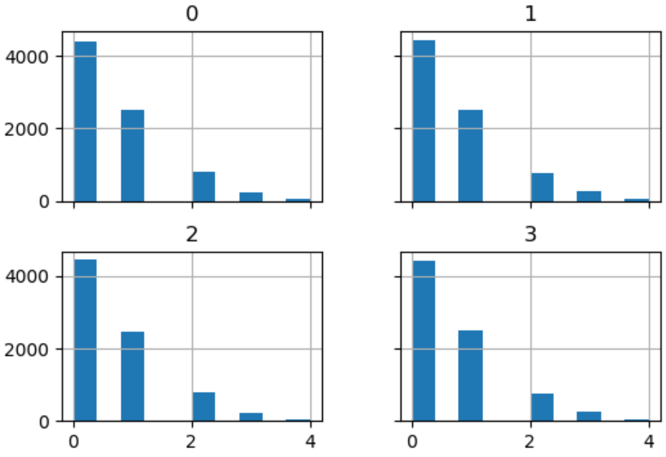

(d) **[Nc=4, M=2000, CD_min=1, CD_max=4]**

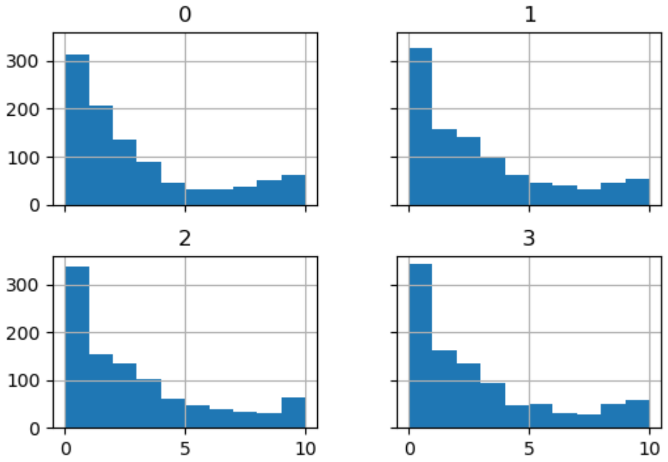

(e) **[Nc=4, M=1000, CD_min=10, CD_max=10]**

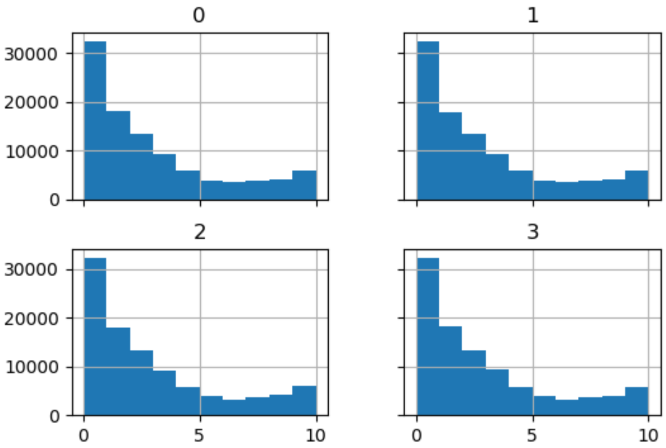

(f) **[Nc=4, M=100000, CD_min=10, CD_max=10]**

Supplementary Figure 2: The distributions are made with the AntiSplodge function: *multinomialSampler(Nc, M, CD_min, CD_max)*, which is the pre-step for sampling profiles. Subsequently profiles are generated by picking cells at random based on the index (integer on top of histgram), representing the cell type index.

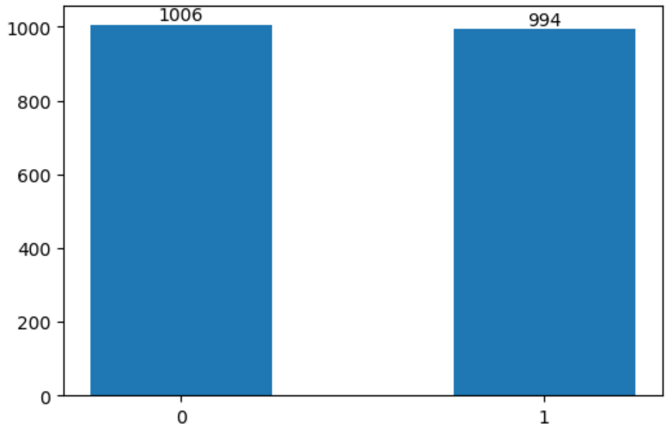

(a) **[Nc=2, M=1000, CD_min=2, CD_max=2]**

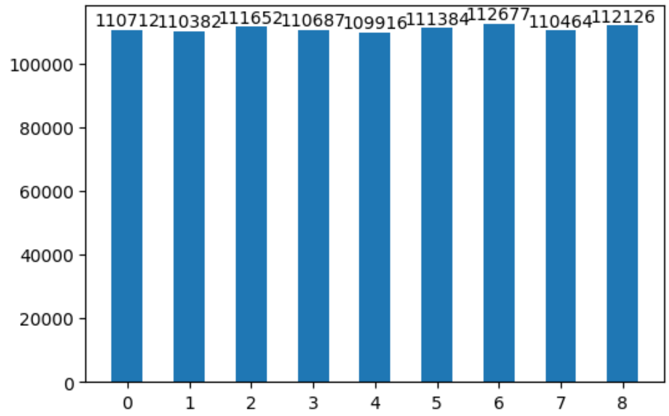

(b) **[Nc=9, M=100000, CD_min=10, CD_max=10]**

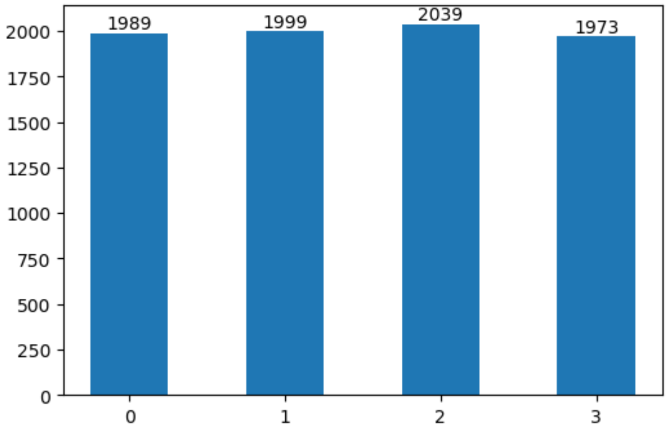

(c) **[Nc=4, M=2000, CD_min=4, CD_max=4]**

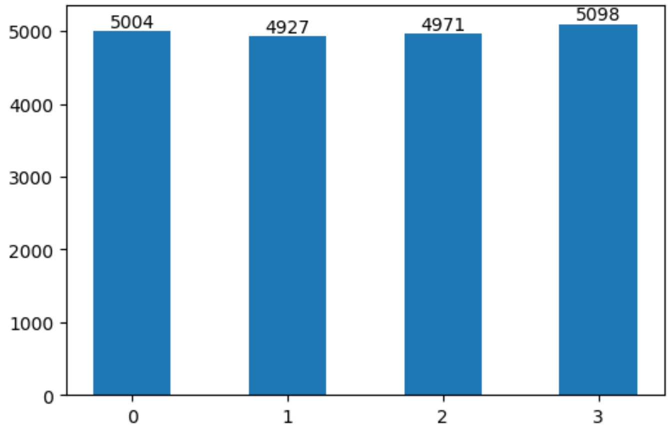

(d) **[Nc=4, M=2000, CD_min=1, CD_max=4]**

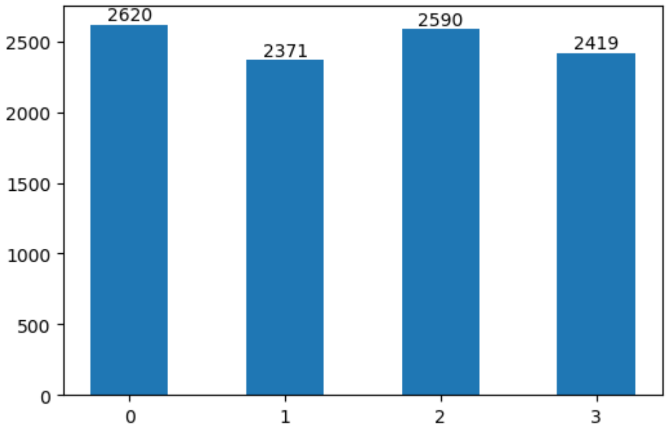

(e) **[Nc=4, M=1000, CD_min=10, CD_max=10]**

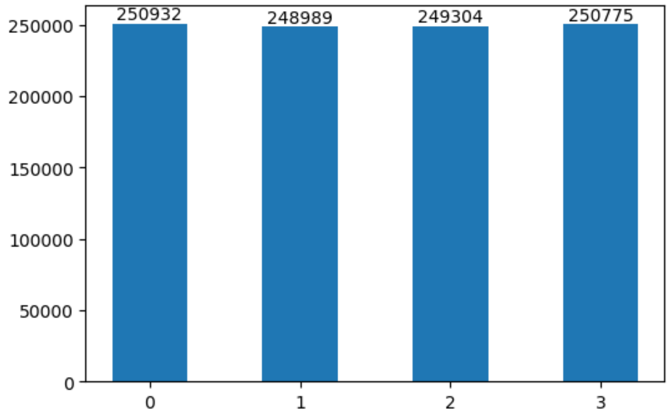

(f) **[Nc=4, M=100000, CD_min=10, CD_max=10]**

Supplementary Figure 3: The counts of each cell type (index, x-axis) made with the AntiSplodge function: *multinomialSampler(Nc, M, CD_min, CD_max)*, which is the pre-step for sampling profiles. These counts matches the respective distributions found in Supplementary Figure 2.

Supplementary Section 2: error function and run-time experiments

Initial experiments

Because of the versatility of *AntiSplodge*, you can supply it with any error function that is compatibly with Pytorch (including custom functions). Therefore, we tested *AntiSplodge* using the standard available error functions that makes sense for the deconvolution problem.

For this we generated synthetic datasets with various sizes but still small compared to what you should do in an actual anaylsis. Below are the overview of datasets (with and without dropout on the first layer), where the numbers in the brackets corresponds to [#training profiles, #validation profiles, #test profiles]. The indices corresponds to the respective columns in Supplementary Table 1 and Supplementary Table 2. For all experiments, we ran 5 warm restarts with a patience counter of 50, using the default AntiSplodge optimizer (Adam with default parameters), profiles contained 1,562 marker-genes found via logistic regression (one cell type vs. all), and synthetic profiles were generated based on cell samples from the Heart Cell Atlas (https://www.heartcellatlas.org/), using the global dataset without normalization. The train, validation, test-split is 90%, 5%, 5%, respectively.

Without dropout:
1=[10000, 5000, 5000], dropout=0
2=[25000, 10000, 10000], dropout=0
3=[50000, 10000, 10000], dropout=0
4=[100000, 10000, 10000], dropout=0
5=[100000, 10000, 20000], dropout=0
6=[100000, 20000, 10000], dropout=0

With dropout:
7=[10000, 5000, 5000], dropout=0.33
8=[25000, 10000, 10000], dropout=0.33
9=[50000, 10000, 10000], dropout=0.33
10=[100.000, 10000, 10000], dropout=0.33
11=[100.000, 10000, 20000], dropout=0.33
12=[100.000, 20000, 10000], dropout=0.33

(The synthetic datasets (and their generated profiles), are the same for 1 and 7, 2 and 8 etc.)

1	2	3	4	5	6	7	8	9	10	11	12	Loss function
**16.09%**	**13.75%**	**14.44%**	**12.69%**	**13.44%**	**12.25%**	**12.49%**	18.41%	26.38%	**11.96%**	**12.92%**	19.00%	**nn.L1Loss()**
18.02%	15.00%	15.81%	14.35%	14.91%	13.88%	13.98%	13.58%	14.63%	13.40%	14.39%	13.55%	**nn.MSELoss()**
17.09%	14.89%	16.09%	13.87%	14.83%	13.79%	13.92%	**13.31%**	**13.94%**	13.67%	14.29%	**13.41%**	**nn.SmoothL1Loss(beta=0.1)**
17.60%	14.95%	16.17%	14.00%	15.56%	14.25%	14.22%	13.37%	14.34%	13.57%	14.15%	13.79%	**nn.SmoothL1Loss(beta=0.25)**
24.30%	14.93%	15.75%	14.03%	15.12%	13.98%	14.45%	13.42%	14.32%	21.57%	14.28%	13.50%	**nn.SmoothL1Loss(beta=0.5)**
17.66%	15.09%	16.55%	14.09%	15.36%	14.01%	14.09%	13.45%	14.66%	13.58%	14.17%	13.62%	**nn.SmoothL1Loss(beta=1)**
17.28%	15.01%	15.92%	14.13%	14.64%	13.92%	13.92%	13.43%	14.52%	13.45%	21.66%	13.69%	**nn.HuberLoss(delta=0.1)**
17.63%	14.96%	15.95%	14.04%	15.59%	13.96%	14.19%	13.41%	14.26%	13.55%	14.12%	13.72%	**nn.HuberLoss(delta=0.25)**
17.36%	15.11%	16.15%	13.97%	15.32%	13.92%	14.14%	13.37%	14.48%	13.43%	14.35%	13.60%	**nn.HuberLoss(delta=0.5)**
33.46%	33.29%	51.76%	32.78%	53.62%	56.23%	32.65%	56.39%	33.45%	54.96%	69.87%	33.00%	**nn.KLDivLoss()**

Supplementary Table 1: Loss function JSD-divergences for small synthetic datasets generated based on cells from the Heart Cell Atlas. Bold text is the lowest divergence for each column.

1	2	3	4	5	6	7	8	9	10	11	12	Loss function
**2.40**	**2.15**	**3.22**	**5.04**	**5.09**	**9.07**	**8.86**	15.33	9.63	**17.62**	**9.48**	15.27	**nn.L1Loss()**
1.42	2.05	3.02	3.77	4.77	7.29	8.31	13.83	8.72	12.89	9.72	14.19	**nn.MSELoss()**
1.66	1.86	2.69	3.92	4.94	6.33	8.94	**12.62**	**9.03**	14.97	9.06	**14.47**	**nn.SmoothL1Loss(beta=0.1)**
1.33	1.80	3.05	3.49	4.65	6.81	8.40	13.05	8.73	14.91	9.10	13.95	**nn.SmoothL1Loss(beta=0.25)**
1.21	1.94	2.67	4.07	4.89	7.98	8.07	13.06	8.98	15.02	9.26	14.09	**nn.SmoothL1Loss(beta=0.5)**
1.17	2.29	2.46	4.07	4.96	6.57	8.17	13.22	9.00	13.00	8.93	15.20	**nn.SmoothL1Loss(beta=1)**
1.84	2.15	2.46	3.44	5.07	8.42	8.90	12.75	8.50	15.52	8.78	13.77	**nn.HuberLoss(delta=0.1)**
2.65	1.84	2.74	4.08	4.76	7.20	8.69	11.50	8.92	14.91	9.41	14.59	**nn.HuberLoss(delta=0.25)**
1.27	2.02	2.52	3.53	4.97	7.81	8.92	12.05	9.07	13.70	10.20	13.70	**nn.HuberLoss(delta=0.5)**
0.95	0.95	2.17	2.20	4.72	4.74	7.72	7.65	7.61	10.78	11.83	8.13	**nn.KLDivLoss()**

Supplementary Table 2: Run-times for each experiment, measured in minutes. Each table cell corresponds to the cell of Supplementary Table 1. Bold text match the best JSD divergence from Supplementary Table 1.

Based on Supplementary Table 1, L1 (nn.L1Loss()) seems to perform best when not used in conjunction with dropout (datasets 1-6), while when dropout is used the top performing method is shared between between L1 and SL1 (Smooth L1 Loss with beta=0.1, datasets 7-12). Although they both use L1 normalization, it seems that the high fluctuation of the L1 might be due to bad initiations or unlucky dropouts which could be dropout of some of the most important marker genes. Prolonging the run-time by increasing the patience counter or by adding more samples should help to decimate this effect.

Overall, the true difference between L1, SmoothL1, MSE, and, Huber seems insignificant, while KLDivLoss doesn't seem to work for this method. Supplementary Table 2 simply shows the run-time of AntiSplodge, which are in the range of 1 to 20 min approximately. We continue for larger datasets with L1 and SmoothL1 (beta=0.1) below.

Extended experiments

This time, we try with larger datasets for L1 and SL1, in the range of 200.000 to 500.000 training samples, only increasing the number of synthethic training profiles. We run these experiments with 100 warm-restarts and a patience threshold of 3.

Without dropout:
1=[200.000, 100.000, 100.000], dropout=0
2=[300.000, 100.000, 100.000], dropout=0
3=[400.000, 100.000, 100.000], dropout=0
4=[500.000, 100.000, 100.000], dropout=0

With dropout:
5=[200.000, 100.000, 100.000], dropout=0.33
6=[300.000, 100.000, 100.000], dropout=0.33
7=[400.000, 100.000, 100.000], dropout=0.33
8=[500.000, 100.000, 100.000], dropout=0.33

(The synthetic datasets (and their generated profiles), are the same for 1 and 5, 2 and 6 etc.)

**1**	**2**	**3**	**4**	**5**	**6**	**7**	**8**	**Loss function**
13.11%	12.37%	12.32%	**11.69%**	12.58%	**11.54%(*)**	11.90%	**11.70%**	**nn.L1Loss()**
14.21%	13.33%	13.14%	12.98%	13.20%	12.61%	12.79%	12.81%	**nn.SmoothL1Loss(beta=0.1)**

Supplementary Table 3: Loss function JSD-divergences for larger datasets. (*) Although this is the lowest JSD, with multiple runs, we would expect it to be larger than column 7 and 8, the increased run-time from Supplementary Table 4 also indicates that we simply ran for a longer period (because we found more better weight settings).

**1**	**2**	**3**	**4**	**5**	**6**	**7**	**8**	**Loss function**
21.70	23.05	45.79	**44.81**	86.38	**101.50**	89.41	**92.20**	**nn.L1Loss()**
22.41	21.12	43.81	44.77	89.11	98.63	88.47	92.68	**nn.SmoothL1Loss(beta=0.1)**

Supplementary Table 4: Run-times for each experiment, measured in minutes. Each table cell corresponds to the cell of Supplementary Table 3. Bold text match the best JSD divergence from Supplementary Table 3.

Based on the results in Supplementary Table 3, it is clear that more training data, means better models (as expected), even with the outlier in column 6 (we ran each experiment once, repeating each experiment multiple times would give us more stable results). It also seems that it is beneficial to use L1 rather than SL1, as L1 on average is approximately 1% point lower than SL1 in terms of JSD.

Realistic-sized experiments

We now try with datasets that are more realistic in terms of how an actual study should proceed, again for L1 and SL1. Only increasing the number of synthethic training profiles. This time in the range of 1 million to 4 million samples.

Without dropout:
1=[1.000.000, 100.000, 100.000], dropout=0
2=[2.000.000, 100.000, 100.000], dropout=0
3=[3.000.000, 100.000, 100.000], dropout=0
4=[4.000.000, 100.000, 100.000], dropout=0

With dropout:
1=[1.000.000, 100.000, 100.000], dropout=0.33
2=[2.000.000, 100.000, 100.000], dropout=0.33
3=[3.000.000, 100.000, 100.000], dropout=0.33
4=[4.000.000, 100.000, 100.000], dropout=0.33

(The synthetic datasets (and their generated profiles), are the same for 1 and 5, 2 and 6 etc.)

Looking at the results for realistic-sized experiments (Supplementary Table 5), we see that overall L1 seems to be the best error function (at least for this dataset), for the 4 million synthetic training profiles dataset, it is approximately 1.5%-points lower than SL1. The difference in results for using drop-out vs. not using drop-out seems insignificant, however, the run-time is considerable larger for the drop-out version (Supplementary Table 6, columns 5-8), this is

1	2	3	4	5	6	7	8	Loss function
11.06%	10.72%	11.03%	**10.46%**	11.05%	10.63%	10.66%	**10.44%**	**nn.L1Loss()**
12.40%	12.21%	12.25%	12.06%	11.91%	11.88%	11.95%	11.93%	**nn.SmoothL1Loss(beta=0.1)**

Supplementary Table 5: Loss function JSD-divergences for realistic-sized datasets.

1	2	3	4	5	6	7	8	Loss function
62.55	98.28	136.68	**216.79**	222.95	250.28	189.76	**340.66**	**nn.L1Loss()**
71.89	101.81	121.97	135.01	190.48	210.09	219.85	341.93	**nn.SmoothL1Loss(beta=0.1)**

Supplementary Table 6: Run-times for each experiment, measured in minutes. Each table cell corresponds to the cell of Supplementary Table 5. Bold text match the best JSD divergence from Supplementary Table 5.

because the number of epochs increases as improvements usually comes in smaller error reductions, because of the missing features at random in the drop-out version. We run these experiments with 100 warm-restarts and a patience threshold of 3.

Conclusion: Overall, L1 loss seems to be the best choice of error function, dropout takes approximately double the number of solutions to reach the same plateau as the non-dropout version, so unless it is needed, use the non-dropout version as using a validation check already solves the over-fitting. Also, use as many training samples as your system can handle, up to a reasonable number. You might think, why doesn't we reach the same JSD as in the comparison figure in the main text, this is because the comparison was made with a quality controlled and preprocessed version of the dataset, while these tests use the dataset as is.

Supplementary Section 3: Comparison

Cell population

In Supplementary Table 7 is the number of each cell type after preprocessing.

**Cell type**	**#**	**Cell type**	**#**	**Cell type**	**#**	**Cell type**	**#**
**Adipocytes**	2,943	**Fibroblast**	54,272	**Myeloid**	10,135	**Smooth_muscle_cells**	8,947
**Atrial_Cardiomyocyte**	22,583	**Lymphoid**	5,189	**Neuronal**	3,137	**Ventricular_Cardiomyocyte**	119,205
**Endothelial**	25,468	**Mesothelial**	387	**Pericytes**	53,609		

Supplementary Table 7: Cell types after preprocessing from the heart cell atlas (https://www.heartcellatlas.org/, dataset="Heart Global")

Marker genes

Below are the marker genes used (in python format, N=1389).

marker_genes = ['ABCA1', 'ABCA6', 'ABCA8', 'ABCA9', 'ABCA9-AS1', 'ABCC1', 'ABCC9', 'ABI1', 'ABI3BP', 'ABL1', 'ABTB2', 'AC003991.1', 'AC005037.1', 'AC005358.1', 'AC005699.1', 'AC007319.1', 'AC008056.1', 'AC008250.1', 'AC009264.1', 'AC010609.1', 'AC011369.1', 'AC011389.1', 'AC012636.1', 'AC013640.1', 'AC013652.1', 'AC015712.2', 'AC016766.1', 'AC016831.7', 'AC017002.5', 'AC018464.1', 'AC018742.1', 'AC019197.1', 'AC020637.1', 'AC022034.2', 'AC022075.1', 'AC027097.2', 'AC058822.1', 'AC068234.2', 'AC079336.4', 'AC084357.2', 'AC091057.6', 'AC091691.2', 'AC092114.1', 'AC092164.1', 'AC092567.1', 'AC092683.1', 'AC098617.1', 'AC100803.3', 'AC109587.1', 'AC109779.1', 'AC113386.1', 'AC114760.2', 'AC114763.1', 'AC120193.1', 'AC130650.1', 'AC140912.1', 'ACACB', 'ACAP1', 'ACKR1', 'ACLY', 'ACSL1', 'ACSL4', 'ACSM1', 'ACSM3', 'ACSS2', 'ACTA1', 'ACTB', 'ACTC1', 'ACTN4', 'ACVR1C', 'ADAM19', 'ADAM28', 'ADAMTS12', 'ADAMTS2', 'ADAMTS4', 'ADAMTS9', 'ADAMTSL1', 'ADAP2', 'ADARB2', 'ADCY3', 'ADCY4', 'ADD3', 'ADGRB3', 'ADGRD1', 'ADGRF5', 'ADGRL3', 'ADH1B', 'ADIPOQ', 'ADIPOR2', 'ADIRF', 'ADK', 'AFAP1L1', 'AFDN', 'AFF3', 'AGAP1', 'AGAP2', 'AGBL1', 'AGBL4', 'AGL', 'AGPAT2', 'AGT', 'AHCTF1', 'AHCYL2', 'AIFM1', 'AKAP12', 'AKAP6', 'AL034397.3', 'AL109930.1', 'AL117190.2', 'AL132671.2', 'AL138828.1', 'AL139383.1', 'AL139807.1', 'AL157871.4', 'AL450384.2', 'ALCAM', 'ALDH1A2', 'ALKBH3', 'AMPD3', 'ANAPC5', 'ANGPTL1', 'ANGPTL4', 'ANK2', 'ANK3', 'ANKRD1', 'ANKRD2', 'ANKRD44', 'ANKRD45', 'ANKS1B', 'ANKUB1', 'ANLN', 'ANO1', 'ANO2', 'ANXA1', 'AP000561.1', 'AP001021.2', 'AP001825.1', 'AP002518.2', 'AP002989.1', 'AP003108.2', 'APBB1IP', 'APCDD1', 'APOD', 'APPBP2', 'AQP1', 'AQP7', 'ARAP2', 'ARGLU1', 'ARHGAP1', 'ARHGAP15', 'ARHGAP18', 'ARHGAP21', 'ARHGAP24', 'ARHGAP31', 'ARID5B', 'ARL15', 'ARMH4', 'ARPC3', 'ASAP1', 'ASB11', 'ASPH', 'ASPN', 'ASTN2', 'ASXL2', 'ATF3', 'ATF6', 'ATG7', 'ATP10A', 'ATP1A2', 'ATP2B1', 'ATP2B4', 'ATRNL1', 'B2M', 'B3GLCT', 'B4GALT5', 'BACH1', 'BACH2', 'BANP', 'BBX', 'BCKDHB', 'BCL11B', 'BCL2', 'BCL6', 'BCO2', 'BHLHE40', 'BICC1', 'BICD1', 'BICDL1', 'BMP2K', 'BMPR1B', 'BMPR2', 'BMS1P14', 'BNC2', 'BOD1L1', 'BRCA1', 'BRCA2', 'BRD3', 'BRIP1', 'BST2', 'BTBD9', 'BTG2', 'BTNL9', 'C14orf180', 'C1QC', 'C1R', 'C2CD2', 'C3', 'C6', 'C7', 'CA1', 'CA4', 'CACNA2D3', 'CADM1', 'CADM2', 'CADPS', 'CALD1', 'CALM2', 'CAMK1D', 'CARD11', 'CARD8', 'CARMIL1', 'CARMN', 'CASQ2', 'CAV1', 'CAV2', 'CC2D2B', 'CCDC102B', 'CCDC18', 'CCDC3', 'CCDC80', 'CCDC88A', 'CCL2', 'CCL21', 'CCND3', 'CCNH', 'CCSER1', 'CD14', 'CD163', 'CD200', 'CD247', 'CD36', 'CD47', 'CD68', 'CD69', 'CD74', 'CD86', 'CD9', 'CDC42BPA', 'CDC42BPB', 'CDC42EP4', 'CDH13', 'CDH19', 'CDH6', 'CDK13', 'CDK15', 'CDKN1A', 'CDON', 'CELF2', 'CEMIP2', 'CENPK', 'CENPP', 'CEP128', 'CEP350', 'CEP95', 'CFH', 'CFLAR', 'CHD9', 'CHL1', 'CHRM3', 'CHST11', 'CHSY3', 'CKMT2', 'CKMT2-AS1', 'CLASP2', 'CLDN1', 'CLDN5', 'CLEC11A', 'CLEC2D', 'CLEC7A', 'CLIC4', 'CLIC5', 'CLK1', 'CLMN', 'CLMP', 'CLSPN', 'CLSTN2', 'CMIP', 'CMTM6', 'CMTM8', 'CNGA1', 'CNOT6L', 'CNR1', 'CNTN4', 'CNTNAP2', 'COBLL1', 'COL12A1', 'COL14A1', 'COL1A1', 'COL1A2', 'COL21A1', 'COL23A1', 'COL24A1', 'COL27A1', 'COL4A1', 'COL4A2', 'COL4A3', 'COL4A4', 'COL4A5', 'COL5A1', 'COL5A2', 'COL6A3', 'COL6A6', 'COL8A1', 'COL9A3', 'COLGALT2', 'COMT', 'COQ10A', 'CORO2B', 'COX4I2', 'CPA3', 'CPAMD8', 'CPED1', 'CPLX3', 'CPM', 'CPNE8', 'CPXM2', 'CRADD', 'CREB5', 'CRIM1', 'CROT', 'CSDC2', 'CSRNP3', 'CSRP3', 'CST3', 'CTDSPL', 'CTNNA1', 'CTNNA3', 'CTNND2', 'CTSB', 'CTSC', 'CXADR', 'CXCL1', 'CXCL2', 'CYB5R3', 'CYP1B1', 'CYP4Z1', 'CYR61', 'CYTIP', 'CYTOR', 'CYYR1', 'DAPK1', 'DAPK2', 'DCBLD1', 'DCLK1', 'DCN', 'DDAH1', 'DDR2', 'DDX17', 'DDX5', 'DECR1', 'DEDD', 'DENND1A', 'DES', 'DGKH', 'DGLUCY', 'DIAPH3', 'DISC1', 'DKK3', 'DLC1', 'DLG4', 'DNAJA1', 'DNAJC1', 'DNAJC6', 'DNM3', 'DOCK10', 'DOCK3', 'DOCK4', 'DOCK6', 'DOCK8', 'DOCK9', 'DPF3', 'DPP6', 'DPYD', 'DPYSL2', 'DST', 'DSTN', 'DTNB', 'DTX3', 'DUSP1', 'DYM', 'DYNC1I1', 'EBF1', 'ECE1', 'ECHDC2', 'EDC3', 'EDF1', 'EDNRA', 'EFEMP1', 'EFL1', 'EFNA5', 'EGFL7', 'EGFR', 'EHBP1', 'EIF1B', 'EIF2AK3', 'EIF3A', 'EIF4A1', 'ELL2', 'ELMO1', 'ELN', 'EMC10', 'EMCN', 'EMP1', 'ENG', 'ENOSF1', 'ENOX1', 'ENPEP', 'ENTPD1', 'ENTPD1-AS1', 'EPAS1', 'EPB41L2', 'EPG5', 'EPHA4', 'EPHB1', 'ERBB4', 'ERBIN', 'ERH', 'ERICH1', 'ERRFI1', 'ESR2', 'ETFB', 'EVA1C', 'EVI5', 'EVL', 'EWSR1', 'EXOC4', 'EXOC6B', 'EXT1', 'EXTL3', 'EYA4', 'EZR', 'F13A1', 'FABP4', 'FABP5', 'FAF1', 'FAM107B', 'FAM111B', 'FAM126B', 'FAM129A', 'FAM13A', 'FAM155A', 'FAM189A2', 'FAM198B-AS1', 'FAM45A', 'FAM49B', 'FAR2', 'FASN', 'FAT3', 'FBLN2', 'FBLN5', 'FBN1', 'FBXL13', 'FBXL7', 'FBXO21', 'FBXW7', 'FCER1G', 'FCHSD2', 'FER', 'FEZ2', 'FGD4', 'FGF14', 'FGF7', 'FGFR1', 'FHIT', 'FHL2', 'FHL5', 'FHOD3', 'FIGN', 'FILIP1L', 'FKBP5', 'FLI1', 'FLNA', 'FLNC-AS1', 'FLT1', 'FMN1', 'FMO2', 'FN1', 'FN3K', 'FNBP1', 'FNDC3B', 'FOS', 'FOSB', 'FOXO1', 'FOXO3', 'FOXP2', 'FRAS1', 'FRK', 'FRMD4A', 'FRMD4B', 'FRMD5', 'FRMPD2', 'FSTL1', 'FTH1', 'FUT8', 'FYB1', 'FYN', 'G0S2', 'GABPB1-AS1', 'GALK2', 'GALNT17', 'GALNT18', 'GAPVD1', 'GAS7', 'GBE1', 'GCNT2', 'GDAP1', 'GFPT2', 'GGT5', 'GHR', 'GIMAP8', 'GLCCI1', 'GLIS3', 'GMDS', 'GNA12', 'GNA14-AS1', 'GNAQ', 'GNLY', 'GOLGA4', 'GPAM', 'GPATCH8', 'GPBP1', 'GPC6', 'GPM6A', 'GPM6B', 'GPR155', 'GPR20', 'GPR34', 'GPRIN3', 'GPX3', 'GRAMD1B', 'GRB10', 'GREB1L', 'GRIK2', 'GRIP1', 'GRIP2', 'GRK3', 'GSE1', 'GTF2I', 'GTF2IRD2', 'GYG2', 'HAMP', 'HAND2-AS1', 'HAS1', 'HCK', 'HCN1', 'HDAC2-AS2', 'HDAC5', 'HDAC9', 'HDC', 'HEATR3', 'HECTD4', 'HECW2', 'HEG1', 'HELLS', 'HERC2', 'HERPUD1', 'HES4', 'HIF1A', 'HIF3A', 'HIGD1B', 'HIST1H2AC', 'HLA-A', 'HLA-B', 'HLA-C', 'HLA-DRA', 'HNRNPDL', 'HNRNPH1', 'HOOK2', 'HP', 'HPGD', 'HSPA12A', 'HSPA9', 'HSPB7', 'HSPH1', 'HTR4', 'HYAL2', 'ICAM2', 'ID1', 'ID2', 'ID3', 'IER2', 'IER3', 'IFI27', 'IFITM2', 'IFNAR1', 'IFNG-AS1', 'IGF1', 'IGF1R', 'IGFBP2', 'IGFBP7', 'IGKC', 'IGSF21', 'IKBKB', 'IKZF1', 'IL16', 'IL18R1', 'IL1R1', 'IL1RAPL1', 'IL1RAPL2', 'IL7R', 'ILK', 'IMMT', 'INPP4A', 'INPP4B', 'INPP5D', 'INSR', 'IPO9-AS1', 'IQGAP2', 'IRAK3', 'ITGA1', 'ITGA7', 'ITGA8', 'ITGA9', 'ITGB1', 'ITGB8', 'ITIH5', 'ITK', 'ITLN1', 'ITPK1', 'ITPKB-AS1', 'ITPR2', 'ITSN1', 'ITSN2', 'IVD', 'JPH2', 'JUN', 'JUNB', 'JUP', 'KALRN', 'KANK1', 'KANK3', 'KANSL1L', 'KAZN', 'KCNAB1', 'KCNE1', 'KCNE4', 'KCNH5',

'KCNJ3', 'KCNJ8', 'KCNMA1', 'KCNMB2', 'KCNMB2-AS1', 'KCNMB4', 'KCNQ5', 'KCNT2', 'KDM5B', 'KIAA0232', 'KIAA0355', 'KIAA1217', 'KIAA1549', 'KIF13A', 'KIF18B', 'KIF20B', 'KIRREL3', 'KIT', 'KLF12', 'KLF13', 'KLF2', 'KLF6', 'KLHL29', 'KMT2A', 'KMT2E', 'KNTC1', 'KRT18', 'KRT19', 'L3MBTL4', 'LAMA2', 'LAMA3', 'LBH', 'LCP2', 'LDB2', 'LDB3', 'LDLRAD3', 'LEMD3', 'LGI4', 'LGMN', 'LGR6', 'LHFPL2', 'LIAS', 'LIMA1', 'LIMCH1', 'LIMS1', 'LINC00486', 'LINC00598', 'LINC00882', 'LINC01088', 'LINC01091', 'LINC01099', 'LINC01411', 'LINC01568', 'LINC01572', 'LINC01592', 'LINC01828', 'LINC02147', 'LINC02208', 'LINC02240', 'LINC02248', 'LINC02253', 'LINC02388', 'LINC02511', 'LINGO1', 'LIPE', 'LIPE-AS1', 'LMCD1', 'LMO7', 'LMOD1', 'LMOD2', 'LOXL2', 'LPAR1', 'LPAR6', 'LPIN1', 'LPL', 'LRIG1', 'LRMDA', 'LRP1', 'LRP1B', 'LRP2', 'LRRTM3', 'LRRTM4', 'LSAMP', 'LSM14A', 'LST1', 'LTBP1', 'LTBP4', 'LYN', 'LYST', 'LZTS1-AS1', 'MACROD2', 'MAGI1', 'MAGI2', 'MAGT1', 'MAML3', 'MAP3K11', 'MAP4', 'MAPK10', 'MAPK4', 'MARC1', 'MARCH1', 'MARCH3', 'MARK3', 'MAST4', 'MBD5', 'MBNL1', 'MCM7', 'MCTP1', 'MCTP2', 'MDFIC', 'ME1', 'MECOM', 'MED13L', 'MEF2C', 'MEG3', 'MEG8', 'MEIS2', 'MELK', 'MEST', 'MFGE8', 'MGAT5', 'MGP', 'MGST1', 'MGST2', 'MGST3', 'MI-CALL2', 'MIPEP', 'MIR193BHG', 'MIR22HG', 'MIR29B2CHG', 'MIR4435-2HG', 'MIR99AHG', 'MITF', 'MLIP', 'MLLT10', 'MLLT6', 'MLXIPL', 'MME', 'MMP16', 'MMRN1', 'MMS22L', 'MPZ', 'MRC1', 'MRC2', 'MS4A6A', 'MSC-AS1', 'MSR1', 'MT1E', 'MT2A', 'MTHFD1L', 'MTHFD2', 'MTUS1', 'MX2', 'MYBL1', 'MYBPC3', 'MYCBP2', 'MYH10', 'MYH11', 'MYH13', 'MYH6', 'MYH7', 'MYH7B', 'MYL2', 'MYL3', 'MYL4', 'MYL6', 'MYL7', 'MYO18B', 'MYO1F', 'MYOCD', 'MYOM2', 'MYPN', 'MYRIP', 'MYSM1', 'N4BP2L2', 'NAA35', 'NAIP', 'NAMPT', 'NAV1', 'NAV2', 'NCAM1', 'NCAM2', 'NDRG4', 'NDUFA4L2', 'NDUFAF8', 'NEAT1', 'NEB', 'NEBL', 'NEDD4L', 'NEGR1', 'NEXN', 'NFATC2', 'NFATC2IP', 'NFIB', 'NFKB1', 'NFKBIA', 'NFKBIZ', 'NFRKB', 'NGF', 'NHS', 'NHSL1', 'NID1', 'NIPBL', 'NKAIN2', 'NKAIN3', 'NKTR', 'NLGN1', 'NLGN4X', 'NLK', 'NLRC5', 'NNMT', 'NOL10', 'NOSTRIN', 'NOVA1', 'NPPA', 'NR2F1', 'NR4A1', 'NR4A3', 'NREP', 'NRG2', 'NRG3', 'NRXN1', 'NRXN3', 'NSD3', 'NSMCE2', 'NTM', 'NTN1', 'NTRK3', 'NUP210', 'NUP210L', 'NUP50-DT', 'NUPR1', 'NUSAP1', 'NXN', 'OBSCN', 'ODF2L', 'OGA', 'OGFRL1', 'OLFM2', 'OPCML', 'ORC2', 'OSBPL10', 'OSMR', 'OTUB1', 'P2RY8', 'PABPC4', 'PACS1', 'PAIP1', 'PALLD', 'PALM2-AKAP2', 'PALMD', 'PAM', 'PAPPA', 'PARD3B', 'PARP14', 'PARP4', 'PARP8', 'PATJ', 'PATL1', 'PBX1', 'PBX3', 'PC', 'PCDH15', 'PCDH7', 'PCDH9', 'PCED1A', 'PCED1B', 'PCSK7', 'PDCD6IP', 'PDE10A', 'PDE1C', 'PDE3B', 'PDE4B', 'PDE4D', 'PDE5A', 'PDE7A', 'PDE7B', 'PDGFD', 'PDGFRB', 'PDK4', 'PDLIM3', 'PDLIM7', 'PDXDC1', 'PDZRN4', 'PECAM1', 'PER1', 'PER3', 'PFKFB3', 'PGAP1', 'PGK1', 'PGM1', 'PHACTR1', 'PHACTR2', 'PHLDB1', 'PHLDB2', 'PIAS2', 'PID1', 'PIGX', 'PIK3R1', 'PIK3R5', 'PIP4K2A', 'PIP5K1B', 'PIR', 'PITPNC1', 'PITPNM2', 'PKHD1L1', 'PKIA', 'PKN2-AS1', 'PKN3', 'PLA2G2A', 'PLCB1', 'PLCB2', 'PLCE1', 'PLCG2', 'PLEKHA5', 'PLEKHG1', 'PLIN1', 'PLIN4', 'PLIN5', 'PLN', 'PLOD2', 'PLPP1', 'PLSCR1', 'PLVAP', 'PLXDC2', 'PLXNA4', 'PMP22', 'PNISR', 'PNPLA2', 'PODXL', 'POLQ', 'POPDC2', 'POSTN', 'POU6F2', 'PPARG', 'PPP1R12A', 'PPP1R13B', 'PPP1R16B', 'PPP1R17', 'PPP1R42', 'PPP2R2B', 'PPP2R5C', 'PPP3CA', 'PRDM16', 'PRELP', 'PRG4', 'PRICKLE1', 'PRIM2', 'PRIMA1', 'PRKAR2A', 'PRKAR2B', 'PRKCA', 'PRKCA-AS1', 'PRKCE', 'PRKCH', 'PRKCQ', 'PRKD1', 'PRKDC', 'PRKG1', 'PRLR', 'PRR16', 'PRRX1', 'PRSS23', 'PSEN1', 'PSMB6', 'PSMB9', 'PSMD4', 'PTEN', 'PTGIS', 'PTK2', 'PTP4A3', 'PTPN2', 'PTPRA', 'PTPRB', 'PTPRC', 'PTPRG', 'PTPRJ', 'PTPRM', 'PTPRQ', 'PTPRS', 'PVT1', 'PXDNL', 'PYGL', 'QKI', 'RAB27B', 'RAB2A', 'RABEP1', 'RABL3', 'RAF1', 'RALGPS1', 'RALGPS2', 'RALYL', 'RAMP3', 'RANBP3L', 'RAP1GAP2', 'RASD1', 'RASGEF1B', 'RASGEF1C', 'RASSF8', 'RBFOX1', 'RBL1', 'RBM47', 'RBMS3-AS2', 'RBPJ', 'RBPMS', 'RCSD1', 'RELN', 'RERG', 'RETSAT', 'REV1', 'RFWD3', 'RGS5', 'RGS6', 'RGS7BP', 'RHBDL3', 'RIN2', 'RIN3', 'RMND5B', 'RNF115', 'RNF121', 'RNF13', 'RNF149', 'RNF150', 'RNF24', 'RNGTT', 'ROBO1', 'ROCK1', 'ROR2', 'RORA', 'RPL10', 'RPL29', 'RPL3L', 'RPLP1', 'RPS15A', 'RPS23', 'RPS25', 'RPS6KA2', 'RPS6KA3', 'RPS6KA5', 'RPS6KC1', 'RRAD', 'RRBP1', 'RSRP1', 'RUNX1', 'RUNX1T1', 'RUNX3', 'S100A6', 'S100A9', 'SAA1', 'SAMD8', 'SAMHD1', 'SAMSN1', 'SASH1', 'SAT1', 'SAV1', 'SCAPER', 'SCD', 'SCMH1', 'SCN3A', 'SCN5A', 'SCN7A', 'SCN9A', 'SCPEP1', 'SDK1', 'SEC14L1', 'SEMA3B', 'SEMA3C', 'SEPT6', 'SERINC5', 'SERPINE2', 'SERPINF1', 'SESTD1', 'SETD5', 'SGCD', 'SGCG', 'SGIP1', 'SGK1', 'SGMS2', 'SH2B3', 'SH2D3C', 'SH3D19', 'SH3PXD2B', 'SH3RF2', 'SH3RF3', 'SHANK3', 'SHISA9', 'SIK2', 'SIK3', 'SIMC1', 'SIPA1L1', 'SIPA1L2', 'SKAP1', 'SKAP2', 'SKIL', 'SLC11A1', 'SLC16A10', 'SLC16A12', 'SLC16A7', 'SLC18A2', 'SLC19A3', 'SLC1A3', 'SLC20A1', 'SLC22A23', 'SLC22A3', 'SLC24A3', 'SLC25A36', 'SLC26A3', 'SLC2A13', 'SLC35F1', 'SLC38A1', 'SLC38A11', 'SLC39A8', 'SLC44A1', 'SLC4A4', 'SLC7A6', 'SLC8A1-AS1', 'SLC9A3R2', 'SLC9A9', 'SLC9C1', 'SLCO1C1', 'SLCO3A1', 'SLIT2', 'SLIT3', 'SLPI', 'SMAD6', 'SMAP1', 'SMAP2', 'SMC5', 'SMCHD1', 'SMIM19', 'SMOC2', 'SMYD2', 'SNAP23', 'SNCG', 'SNED1', 'SNHG9', 'SNTB2', 'SNTG2', 'SNX1', 'SNX31', 'SOCS3', 'SORBS1', 'SORBS2', 'SORCS1', 'SOX5', 'SOX6', 'SPACA6', 'SPATA6', 'SPIDR', 'SPNS2', 'SPON1', 'SPRED1', 'SPSB1', 'SQSTM1', 'SRBD1', 'SREBF1', 'SRGAP2', 'SRGN', 'SRPK2', 'SRPX', 'SRPX2', 'SRSF11', 'SSBP2', 'ST6GAL1', 'ST6GALNAC3', 'ST6GALNAC5', 'ST7', 'ST8SIA6', 'STAB1', 'STARD13', 'STC1', 'STEAP4', 'STK4', 'STOX2', 'STT3A', 'STX3', 'STXBP5', 'SULF1', 'SVEP1', 'SYNE1', 'SYNE2', 'SYNPO2', 'SYNPR', 'SYNRG', 'SYTL3', 'TACC1', 'TAF1D', 'TAGLN', 'TBC1D14', 'TBC1D22B', 'TBCK', 'TBL1XR1', 'TBX20', 'TBX5', 'TBXAS1', 'TC2N', 'TCEAL9', 'TCF4', 'TCF7L2', 'TCIM', 'TEAD1', 'TEC', 'TECPR2', 'TENM2', 'TENM3', 'TENM4', 'TENT5A', 'TET3', 'TEX14', 'TFPI', 'TGFB3', 'TGFBR3', 'THBS1', 'THBS2', 'THEMIS', 'THRAP3', 'THSD4', 'THUMPD1', 'THUMPD3', 'TIE1', 'TIMP1', 'TIMP3', 'TLL1', 'TLN2', 'TM4SF1', 'TMCO1', 'TMCO4', 'TMEFF2', 'TMEM106A', 'TMEM108', 'TMEM131L', 'TMEM132C', 'TMEM135', 'TMEM161B-AS1', 'TMEM163', 'TMEM178B', 'TMEM51', 'TMEM88', 'TMSB10', 'TMSB4X', 'TMTC1', 'TNC', 'TNFAIP6', 'TNFRSF21', 'TNIK', 'TNKS2', 'TNNI3', 'TNNT2', 'TNNT3', 'TNRC18', 'TNRC6B', 'TNRC6C', 'TNS1', 'TNXB', 'TOGARAM2', 'TOPBP1', 'TPM2', 'TPM4', 'TPO', 'TPRG1', 'TPSB2', 'TRAF3', 'TRAPPC10', 'TRDN', 'TRERF1', 'TRIM54', 'TRIO', 'TRMT9B', 'TRPS1', 'TSC22D1', 'TSHZ2', 'TSHZ3', 'TSPAN9', 'TTLL7', 'TXLNB', 'TYROBP', 'U91319.1', 'UAP1', 'UBAC1', 'UBAC2', 'UBC', 'UBE2E1', 'UBE2E2', 'UBE2R2', 'UBE3A', 'UGT2B4', 'UPF2', 'UPF3A', 'USP15', 'USP32', 'USP36', 'USP42', 'USP53', 'VAMP5', 'VDAC2', 'VEGFC', 'VIPR2', 'VIT', 'VKORC1L1', 'VMP1', 'VOPP1', 'VPS8', 'VWC2', 'VWF', 'WAPL', 'WDR20', 'WDR76', 'WIPF1', 'WSB1', 'WTIP', 'WWC1', 'WWOX', 'XAF1', 'XIRP2', 'XIST', 'XKR4', 'YWHAE', 'ZBTB20', 'ZBTB7C', 'ZC3H6', 'ZDHHC14', 'ZEB1', 'ZEB2', 'ZFHX3', 'ZFP36', 'ZFP36L1', 'ZFP36L2', 'ZFPM2', 'ZGRF1', 'ZMYM2', 'ZMYND11', 'ZNF337', 'ZNF385B', 'ZNF521', 'ZNF532', 'ZNF536', 'ZNF608', 'ZNF630', 'ZNF644', 'ZNRF3', 'ZRANB2-AS1', 'ZSWIM6']

Supplementary Section 4: Developmental human heart analysis

Cell population

In Supplementary Table 8 is the number of each cell type after preprocessing. Note, this is a rather small scRNA dataset (N=3717 cells), but we can still sample as many synthetic spatial transcriptomics profiles as we wish

Cell type	#	Cell type	#	Cell type	#	Cell type	#
Atrial cardiomyocytes	152	Epicardial cells	128	Fibroblast-like (related to larger vascular development)	150	Smooth muscle cells / fibroblast-like)	263
Capillary endothelium	662	Epicardium-derived cells	389	Fibroblast-like (related to smaller vascular development)	341	Ventricular cardiomyocytes	497
Cardiac neural crest cells & Schwann progenitor cells	75	Erythrocytes	298	Immune cells	75		
Endothelium / pericytes / adventitia	127	Fibroblast-like (related to cardiac skeleton connective tissue)	462	Myoz2-enriched cardiomyocytes	97		

Supplementary Table 8: Cell types after preprocessing from the developmental human heart study (https://www.spatialresearch.org/resources-published-datasets/doi-10-1016-j-cell-2019-11-025/)

Predicted cell type proportions

Based on the trained model (one for all samples, based on the accompanied scRNA) for Application 1, these are the predicted proportions for each cell type, shown for each tissue sample (zoom in to see the numbers).

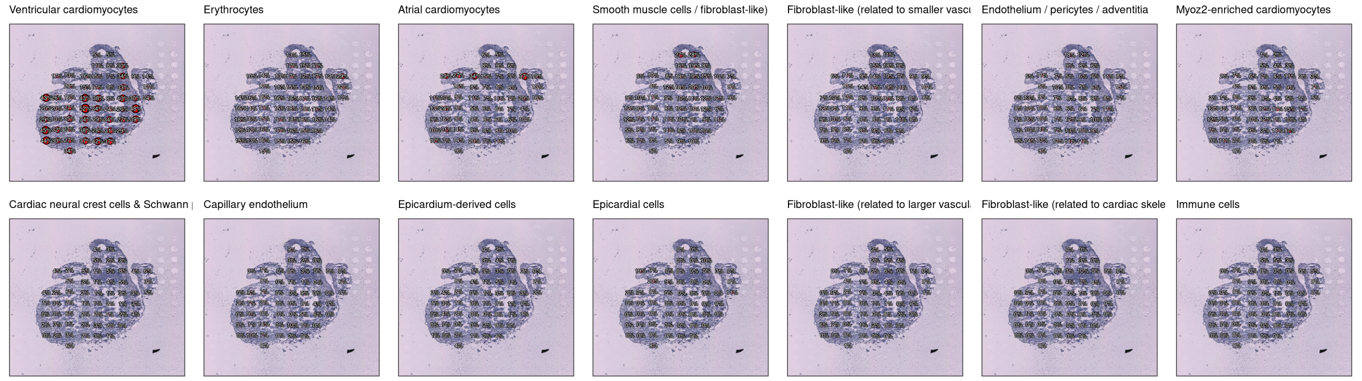

Supplementary Figure 4: Developmental human heart - Sample #1

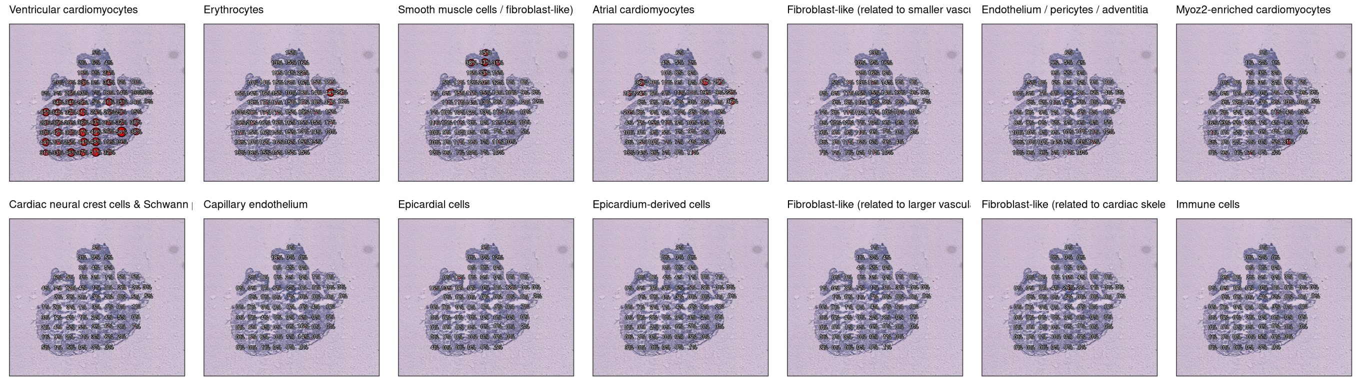

Supplementary Figure 5: Developmental human heart - Sample #2

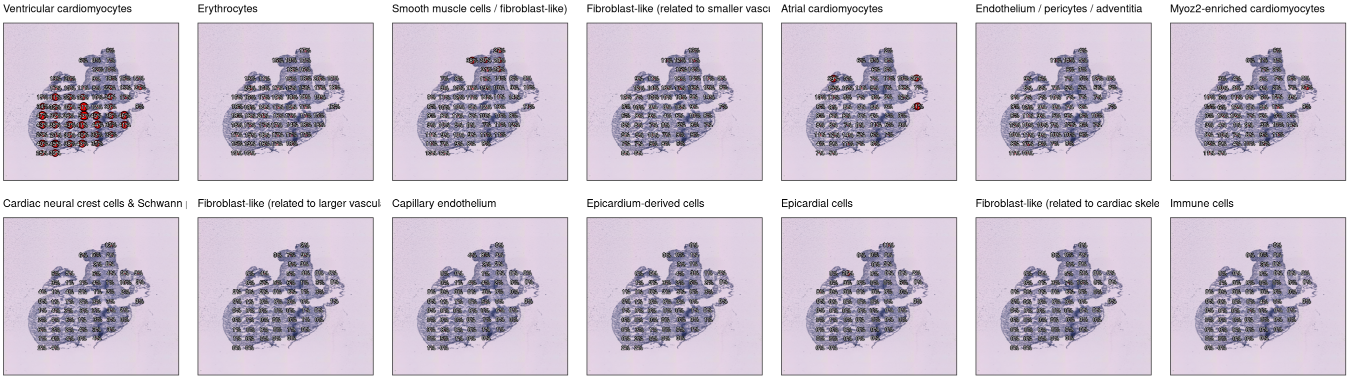

Supplementary Figure 6: Developmental human heart - Sample #3

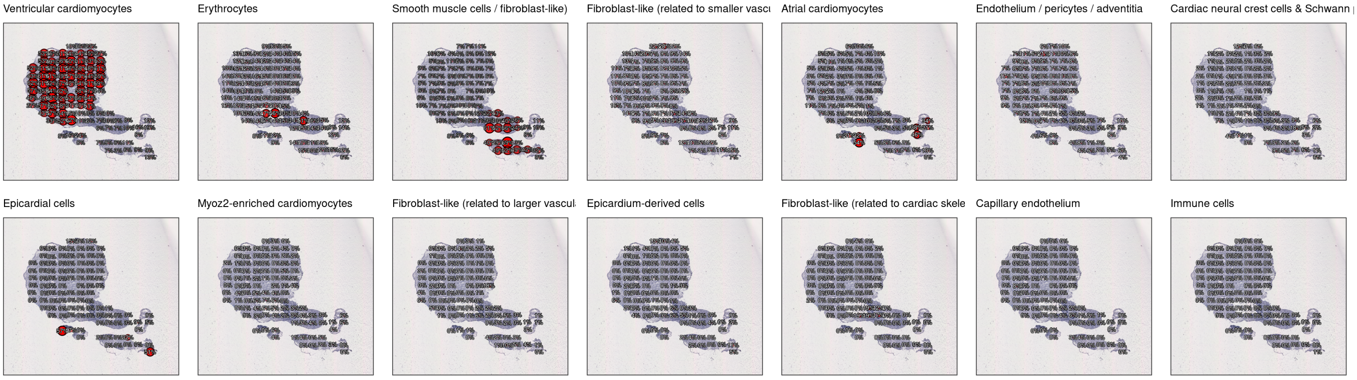

Supplementary Figure 7: Developmental human heart - Sample #4

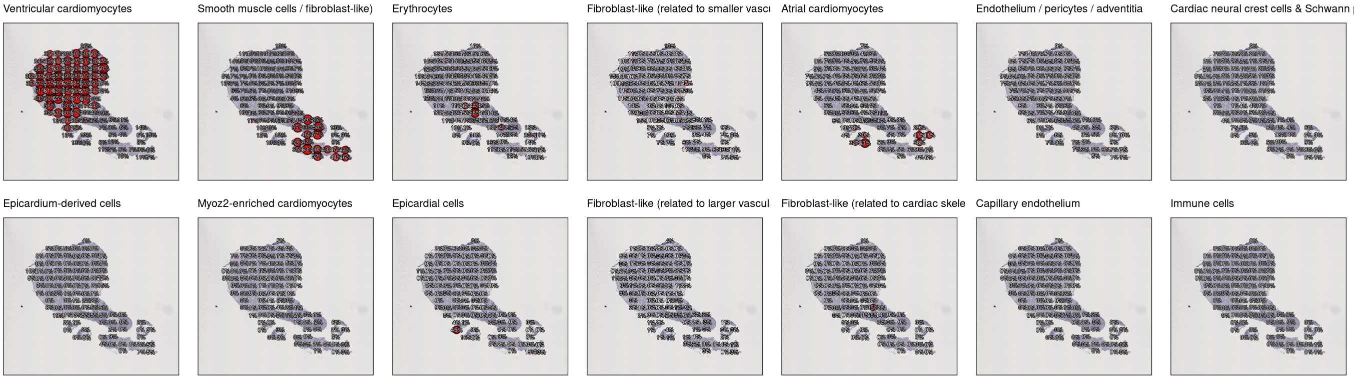

Supplementary Figure 8: Developmental human heart - Sample #5

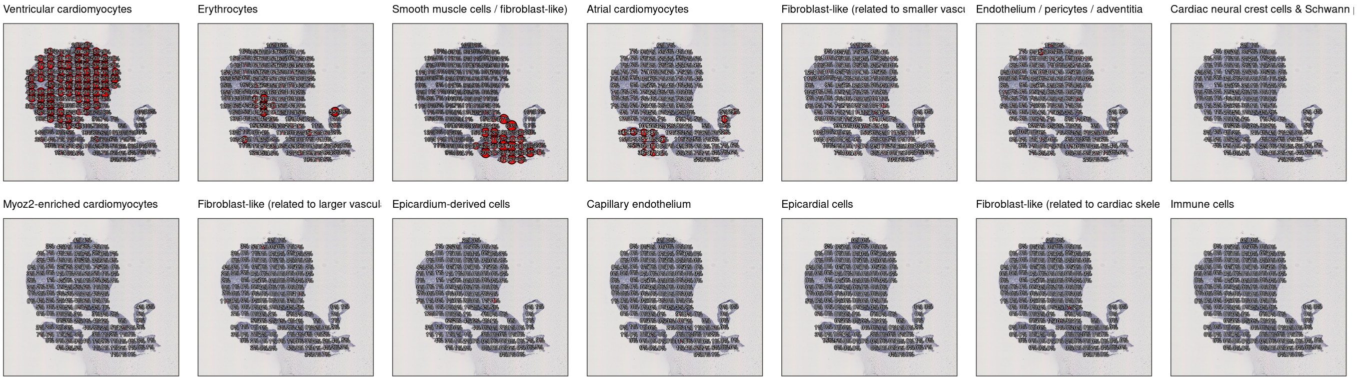

Supplementary Figure 9: Developmental human heart - Sample #6

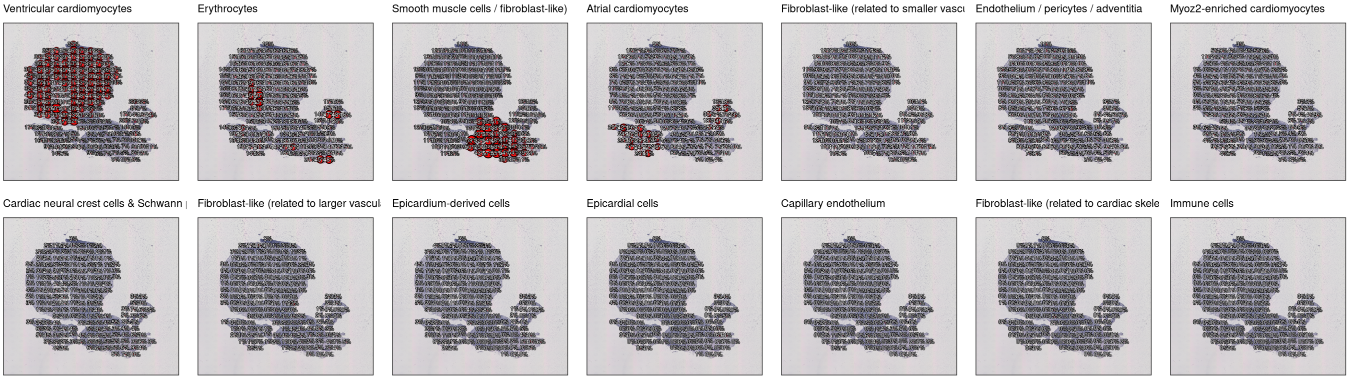

Supplementary Figure 10: Developmental human heart - Sample #7

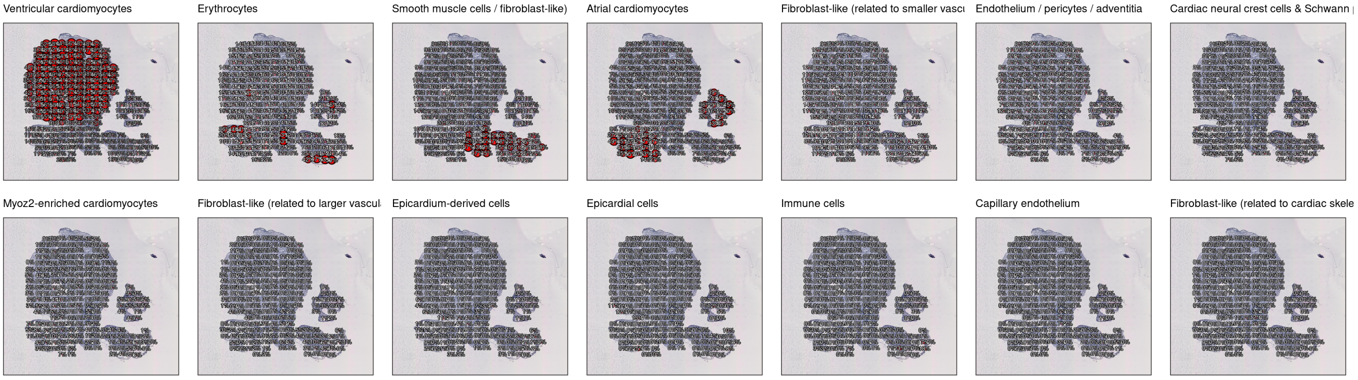

Supplementary Figure 11: Developmental human heart - Sample #8

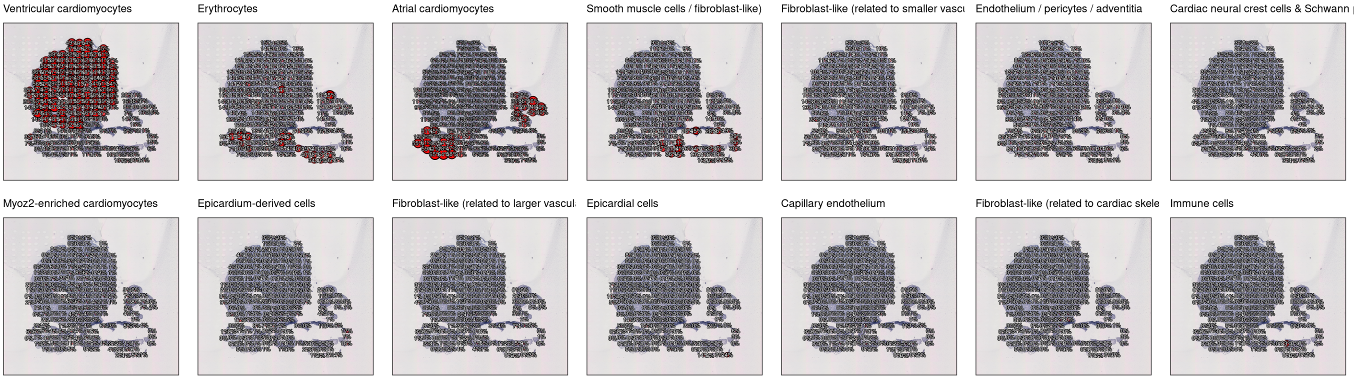

Supplementary Figure 12: Developmental human heart - Sample #9

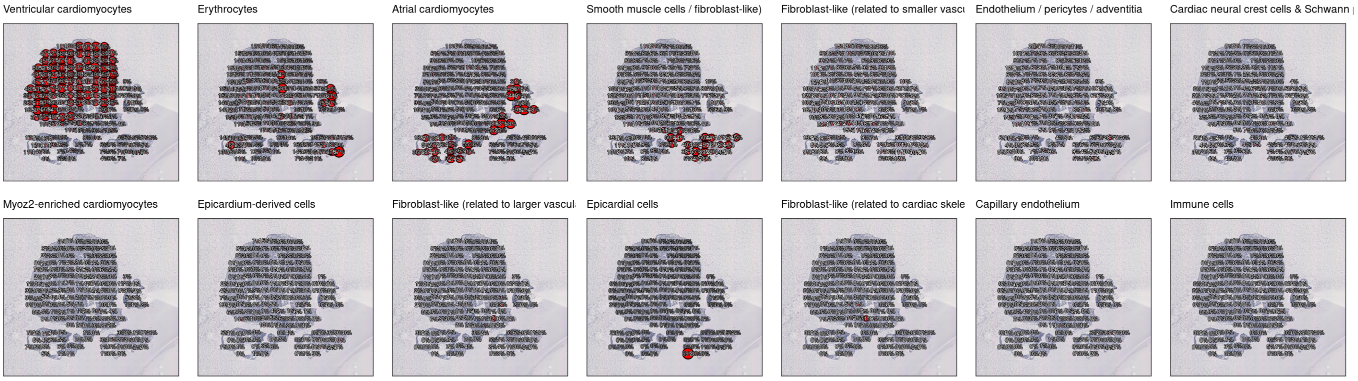

Supplementary Figure 13: Developmental human heart - Sample #10

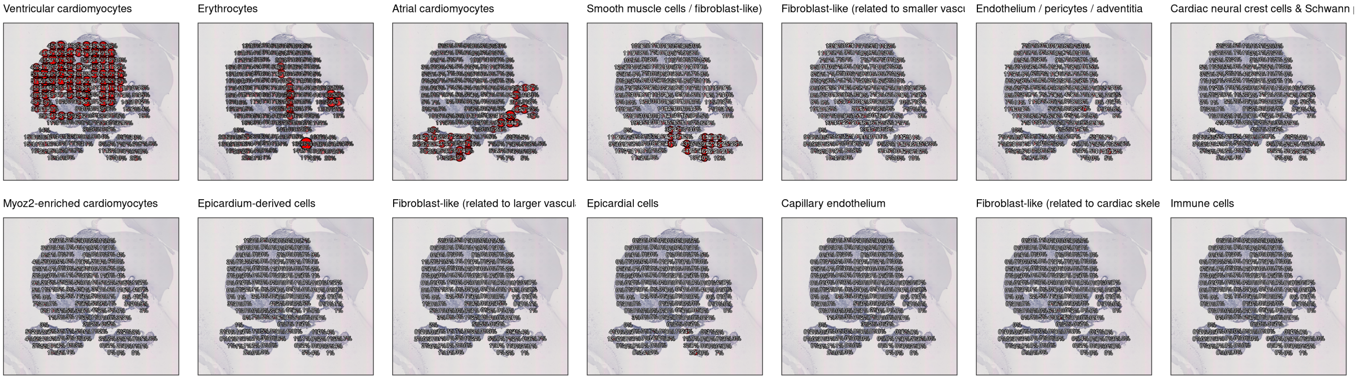

Supplementary Figure 14: Developmental human heart - Sample #11

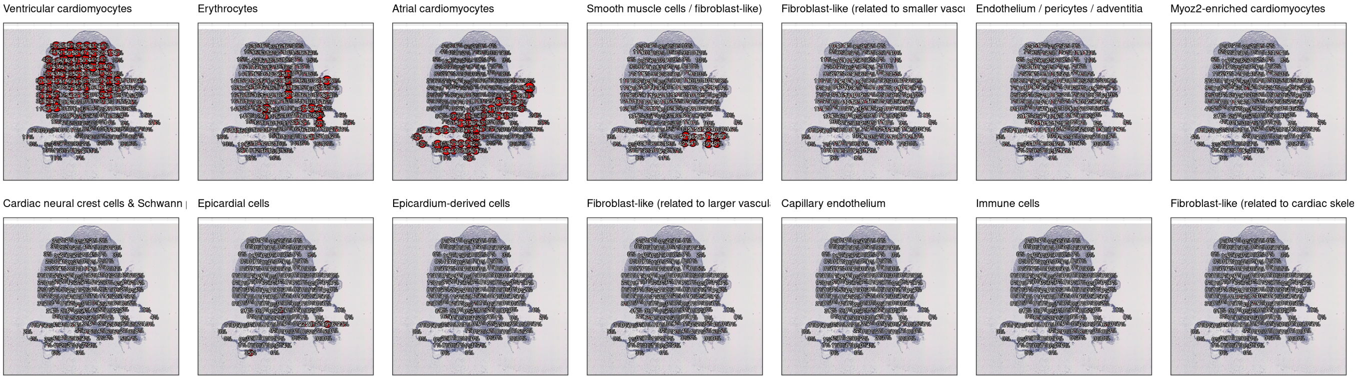

Supplementary Figure 15: Developmental human heart - Sample #12

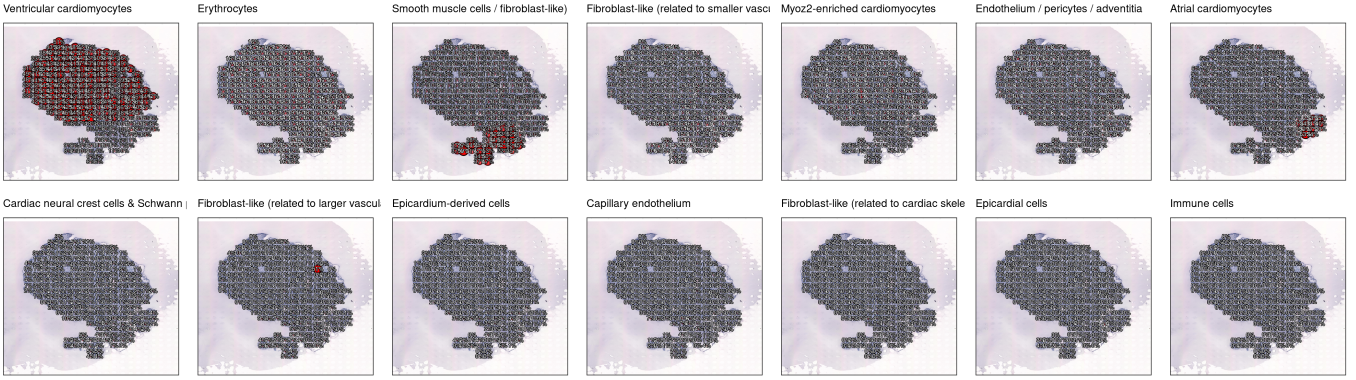

Supplementary Figure 16: Developmental human heart - Sample #13

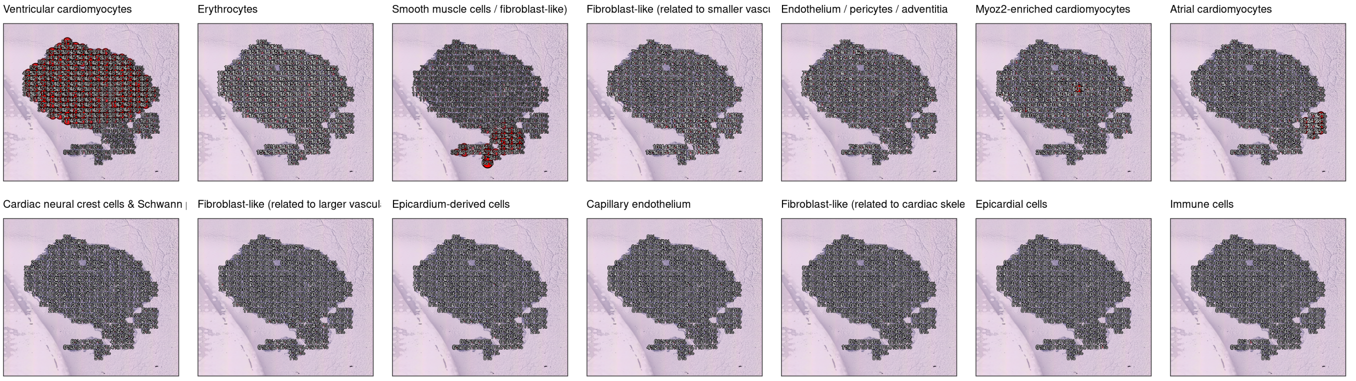

Supplementary Figure 17: Developmental human heart - Sample #14

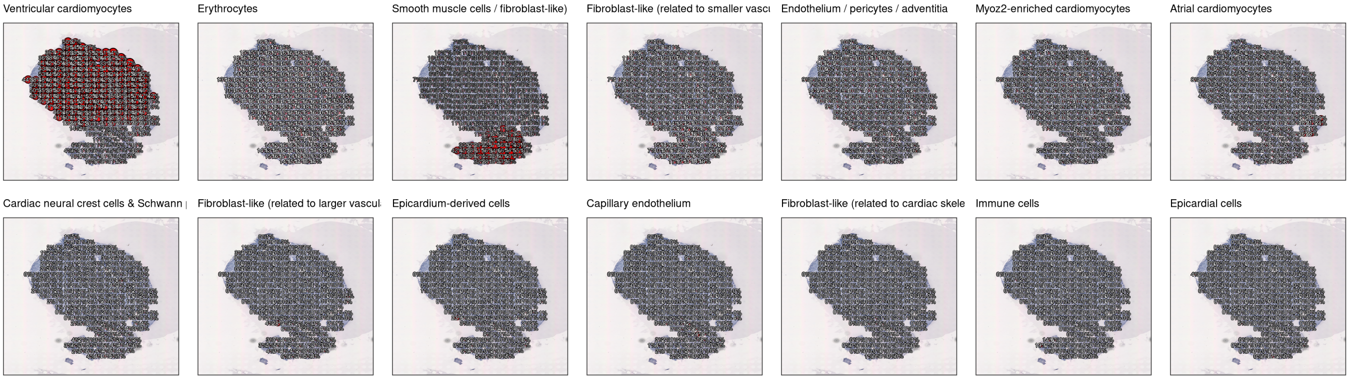

Supplementary Figure 18: Developmental human heart - Sample #15

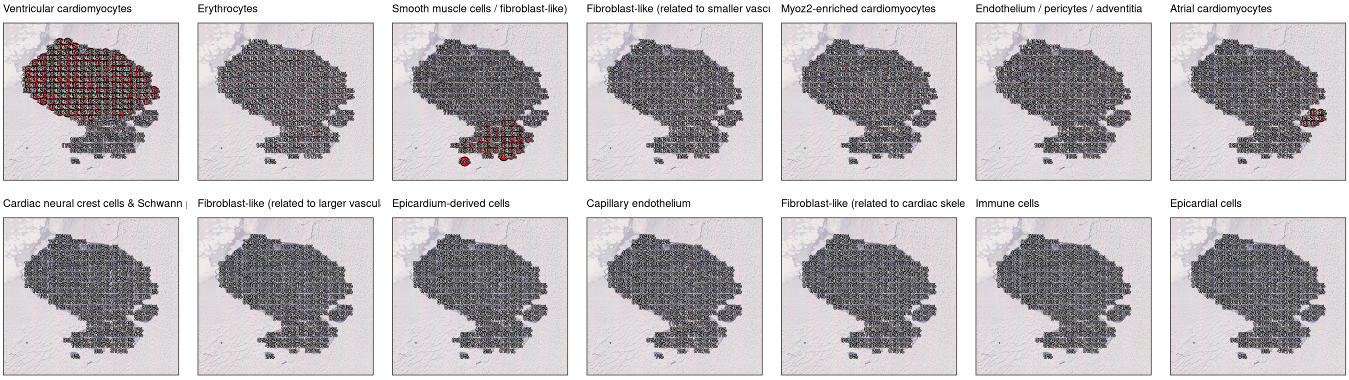

Supplementary Figure 19: Developmental human heart - Sample #16

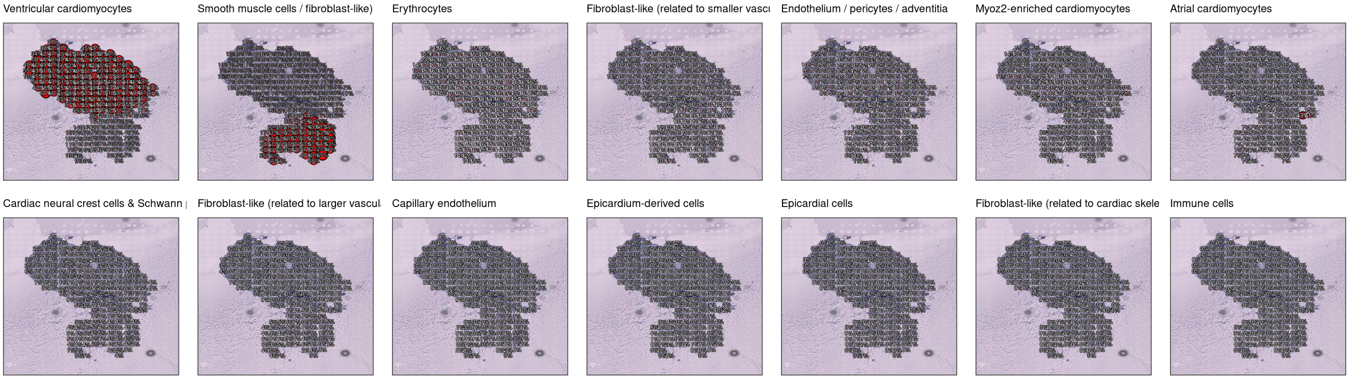

Supplementary Figure 20: Developmental human heart - Sample #17

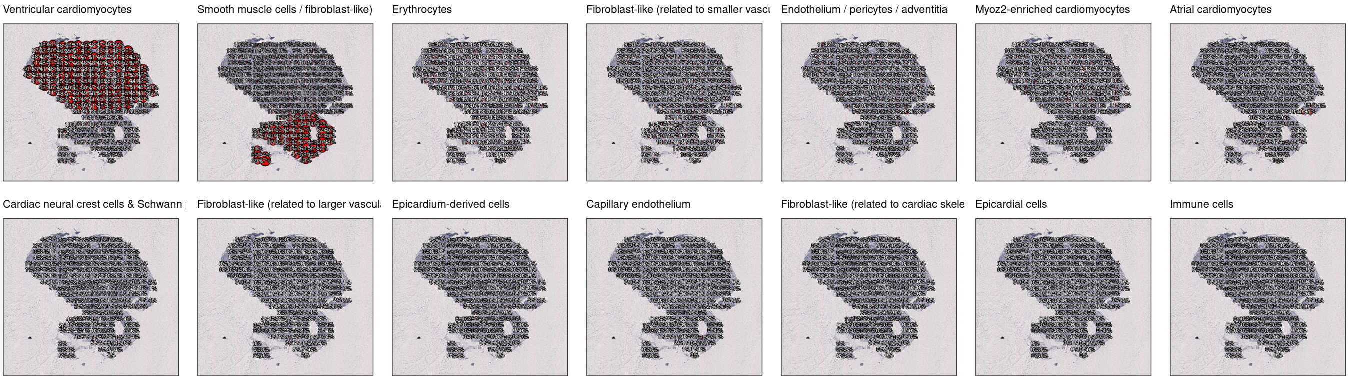

Supplementary Figure 21: Developmental human heart - Sample #18

Marker genes

Below are the marker genes used (in python format, N=1387).

marker_genes = ['A2M', 'ABCA8', 'ACOT13', 'ACP1', 'ACTA1', 'ACTA2', 'ACTB', 'ACTC1', 'ACTG1', 'ACTG2', 'ACTN1', 'ACTN2', 'ACTN4', 'ACTR2', 'ACTR3', 'ACVRL1', 'ACYP1', 'ADAM15', 'ADAMTS6', 'ADAMTS9', 'ADD3', 'ADGRL4', 'ADIPOR1', 'AFAP1L1', 'AGRN', 'AHNAK', 'AKAP12', 'AKAP9', 'AKR1A1', 'AKR1B1', 'ALCAM', 'ALDH1A2', 'ALDH2', 'ALDOA', 'ALKBH7', 'ANAPC16', 'ANGPTL2', 'ANKRD1', 'ANKRD9', 'ANP32B', 'ANXA1', 'ANXA2', 'ANXA3', 'ANXA5', 'ANXA6', 'ANXA7', 'AP1S2', 'AP2M1', 'AP2S1', 'AP3S1', 'APCDD1', 'APEX1', 'APLNR', 'APLP1', 'APLP2', 'APOBEC2', 'APOE', 'APP', 'APRT', 'ARF4', 'ARGLU1', 'ARHGAP29', 'ARHGDIA', 'ARHGDIB', 'ARID5B', 'ARL4D', 'ARL6IP1', 'ARL6IP5', 'ARMCX3', 'ARPC1B', 'ARPC2', 'ARPC3', 'ARPC4', 'ARPC5', 'ARRB2', 'ASAH1', 'ASH2L', 'ASPN', 'ASS1', 'ATF4', 'ATOH8', 'ATOX1', 'ATP1A1', 'ATP1B1', 'ATP1B3', 'ATP2A2', 'ATP2B1', 'ATP6V0B', 'ATP6V0E1', 'ATP6V1F', 'ATP6V1G1', 'ATRX', 'AURKA', 'AXL', 'B2M', 'BAALC', 'BAG1', 'BAMBI', 'BANF1', 'BASP1', 'BAX', 'BCAM', 'BCL2L1', 'BCL7C', 'BEX1', 'BEX3', 'BGN', 'BID', 'BIN1', 'BIRC5', 'BLVRB', 'BMP4', 'BNIP3', 'BNIP3L', 'BPGM', 'BRI3', 'BSG', 'BST2', 'BTF3', 'BTG1', 'BTG2', 'BUB3', 'BZW2', 'C11orf96', 'C12orf57', 'C12orf75', 'C1D', 'C1QA', 'C1orf122', 'C20orf27', 'C4orf3', 'C4orf48', 'C7', 'C7orf50', 'C9orf78', 'CACYBP', 'CADM1', 'CALCRL', 'CALD1', 'CALM1', 'CALM2', 'CALR', 'CALU', 'CAMK2D', 'CAMTA1', 'CAP1', 'CAP2', 'CAPZA2', 'CAPZB', 'CARHSP1', 'CAT', 'CAV1', 'CAV2', 'CBX5', 'CCDC3', 'CCDC34', 'CCDC80', 'CCDC85B', 'CCDC88A', 'CCL2', 'CCL21', 'CCNA2', 'CCNB1', 'CCNB2', 'CCND1', 'CCNDBP1', 'CCNG1', 'CCNI', 'CCNL1', 'CCS', 'CCT3', 'CCT5', 'CCT8', 'CD14', 'CD151', 'CD24', 'CD248', 'CD34', 'CD36', 'CD37', 'CD40', 'CD47', 'CD59', 'CD63', 'CD9', 'CD93', 'CD99', 'CDC20', 'CDC37', 'CDC42', 'CDC42EP1', 'CDC42EP3', 'CDH11', 'CDH13', 'CDH2', 'CDH5', 'CDK1', 'CDKN1A', 'CDKN2D', 'CDKN3', 'CEBPB', 'CEBPD', 'CENPF', 'CENPV', 'CENPW', 'CFAP20', 'CFL1', 'CFL2', 'CGNL1', 'CHCHD10', 'CHCHD2', 'CHCHD7', 'CHD9', 'CIRBP', 'CISD1', 'CISD2', 'CITED2', 'CKB', 'CKLF', 'CKM', 'CKS1B', 'CLEC11A', 'CLIC1', 'CLTA', 'CLTC', 'CLU', 'CMC2', 'CMTM3', 'CMTM6', 'CMYA5', 'CNBP', 'CNDP2', 'CNN1', 'CNN2', 'CNN3', 'CNP', 'CNPY2', 'CNPY3', 'CNTFR', 'COL14A1', 'COL15A1', 'COL18A1', 'COL1A1', 'COL1A2', 'COL21A1', 'COL2A1', 'COL3A1', 'COL4A1', 'COL4A2', 'COL5A1', 'COL5A2', 'COL6A1', 'COL6A2', 'COL6A3', 'COL6A6', 'COL9A1', 'COL9A2', 'COL9A3', 'COLEC11', 'COMMD6', 'CORO1B', 'COTL1', 'COX14', 'COX4I1', 'COX5A', 'COX5B', 'COX6A1', 'COX6A2', 'COX6B1', 'COX6C', 'COX7A1', 'COX7A2', 'COX7A2L', 'COX7B', 'COX7C', 'COX8A', 'CPE', 'CPVL', 'CRABP2', 'CREB5', 'CREG1', 'CRIP2', 'CRISPLD1', 'CRMP1', 'CRYAB', 'CSRP1', 'CSRP2', 'CSRP3', 'CST3', 'CSTB', 'CTHRC1', 'CTNNAL1', 'CTNNBIP1', 'CTSB', 'CTSC', 'CTSD', 'CTSK', 'CTSL', 'CTSZ', 'CUEDC2', 'CUTA', 'CXADR', 'CXCL12', 'CXCR4', 'CXXC5', 'CYB5A', 'CYBA', 'CYC1', 'CYCS', 'CYFIP1', 'CYYR1', 'DAAM1', 'DAB2', 'DAD1', 'DAZAP2', 'DBI', 'DBNL', 'DCAF12', 'DCN', 'DDAH2', 'DDIT3', 'DDOST', 'DDX5', 'DECR1', 'DEK', 'DES', 'DKK3', 'DLC1', 'DMD', 'DMKN', 'DNAJA1', 'DNAJC1', 'DNPH1', 'DPM2', 'DPP7', 'DPYSL3', 'DRAM2', 'DSP', 'DST', 'DSTN', 'DUSP1', 'DUSP6', 'DUT', 'DYNLL1', 'ECE1', 'ECI1', 'EDF1', 'EDIL3', 'EDN1', 'EDNRA', 'EDNRB', 'EEF1A1', 'EEF1B2', 'EEF1D', 'EEF2', 'EFEMP2', 'EFNA1', 'EFNA5', 'EGFL7', 'EGR1', 'EID1', 'EIF1', 'EIF1B', 'EIF3A', 'EIF3E', 'EIF3F', 'EIF3H', 'EIF3K', 'EIF3M', 'EIF4A2', 'EIF4EBP1', 'EIF4G2', 'EIF5', 'EIF5A', 'ELK3', 'ELN', 'ELOF1', 'EMC10', 'EMC3', 'EMCN', 'EMILIN1', 'EMP1', 'EMP2', 'EMP3', 'ENAH', 'ENC1', 'ENG', 'ENO1', 'ENO3', 'ENPP1', 'ENY2', 'EPAS1', 'EPB41L2', 'EPHA7', 'ERP29', 'ESAM', 'ETFB', 'ETV1', 'EVA1B', 'EVL', 'EZR', 'F13A1', 'F2R', 'FABP3', 'FABP5', 'FAM104A', 'FAM110B', 'FAM126A', 'FAM162A', 'FAM200B', 'FAM210B', 'FAM43A', 'FAU', 'FBLN1', 'FBLN2', 'FBLN5', 'FBN1', 'FBN2', 'FBXO32', 'FBXO7', 'FCGRT', 'FDPS', 'FECH', 'FERMT2', 'FGF12', 'FGFR1OP2', 'FH', 'FHL1', 'FHL2', 'FHL3', 'FILIP1', 'FIS1', 'FITM1', 'FJX1', 'FKBP1A', 'FKBP8', 'FLI1', 'FLNA', 'FLNB', 'FLNC', 'FLRT2', 'FMOD', 'FN1', 'FOS', 'FOSB', 'FOXC1', 'FOXP1', 'FREM1', 'FSCN1', 'FSTL1', 'FTH1', 'FUS', 'FXYD5', 'FXYD6', 'FZD1', 'GABARAPL2', 'GADD45A', 'GADD45B', 'GADD45GIP1', 'GAMT', 'GAPDH', 'GAS1', 'GAS6', 'GATA6', 'GATA6-AS1', 'GATM', 'GBP4', 'GDI1', 'GHITM', 'GJA1', 'GJA4', 'GJA5', 'GLIPR2', 'GLRX', 'GLRX5', 'GLUL', 'GMDS', 'GMPR', 'GNAI2', 'GNAS', 'GNG11', 'GNG5', 'GOT1', 'GPC3', 'GPC6', 'GPI', 'GPM6B', 'GPSM3', 'GPX1', 'GPX4', 'GPX8', 'GRB2', 'GRN', 'GSN', 'GSPT1', 'GSTM3', 'GSTO1', 'GSTP1', 'GTF3A', 'GUCY1A2', 'GUK1', 'GYG1', 'HACD1', 'HAGH', 'HAND2', 'HAPLN1', 'HCFC1R1', 'HDAC2', 'HEBP2', 'HERPUD1', 'HES4', 'HIGD1A', 'HIGD2A', 'HINT1', 'HK1', 'HLA-A', 'HLA-B', 'HLA-C', 'HLA-E', 'HMBS', 'HMGA1', 'HMGB1', 'HMGB2', 'HMGB3', 'HMGN1', 'HMGN2', 'HMGN3', 'HNMT', 'HNRNPA0', 'HNRNPA1', 'HNRNPA2B1', 'HNRNPA3', 'HNRNPC', 'HNRNPD', 'HNRNPDL', 'HNRNPK', 'HNRNPR', 'HSBP1', 'HSP90AA1', 'HSP90AB1', 'HSP90B1', 'HSPA5', 'HSPA8', 'HSPB1', 'HSPB2', 'HSPB3', 'HSPB7', 'HSPD1', 'HSPE1', 'HSPG2', 'HTRA1', 'HYAL2', 'ICAM2', 'ID1', 'ID2', 'ID3', 'ID4', 'IDH2', 'IER2', 'IER3', 'IFITM2', 'IFITM3', 'IFNGR1', 'IGF2', 'IGFBP2', 'IGFBP3', 'IGFBP4', 'IGFBP5', 'IGFBP7', 'IL11RA', 'IL33', 'INHBA', 'INPP1', 'IPO11', 'ISCA1', 'ISCU', 'ISLR', 'ISYNA1', 'ITGA1', 'ITGA5', 'ITGA8', 'ITGB1', 'ITLN1', 'ITM2B', 'ITM2C', 'JAG1', 'JAM2', 'JTB', 'JUN', 'JUNB', 'JUP', 'KCNJ8', 'KCTD12', 'KDELR2', 'KDR', 'KHDRBS1', 'KIF21A', 'KLF6', 'KPNA2', 'KRT18', 'KRT19', 'KRT8', 'KTN1', 'LAMA4', 'LAMA5', 'LAMB1', 'LAMB2', 'LAMC1', 'LAMTOR1', 'LAMTOR2', 'LAMTOR4', 'LAMTOR5', 'LAP3', 'LAPTM4A', 'LAPTM4B', 'LAPTM5', 'LBH', 'LDB3', 'LDHA', 'LDHB', 'LEPR', 'LEPROT', 'LEPROTL1', 'LGALS1', 'LGALS3', 'LGALS3BP', 'LGMN', 'LIMA1', 'LIMCH1', 'LIMS1', 'LINC00881', 'LIPA', 'LITAF', 'LMCD1', 'LMNA', 'LMO2', 'LMO4', 'LMO7', 'LMOD2', 'LOXL1', 'LOXL2', 'LRP1', 'LRRC10', 'LRRC17', 'LRRC49', 'LSM4', 'LSM7', 'LTBP1', 'LTBP4', 'LUC7L3', 'LUM', 'LUZP1', 'LY6E', 'LYL1', 'LYN', 'MAF', 'MAF1', 'MAGED2', 'MANF', 'MAP1B', 'MAP1LC3A', 'MAP1LC3B', 'MARCKS', 'MARCKSL1', 'MASP1', 'MATN2', 'MB', 'MCAM', 'MCL1', 'MDFI', 'MDH1', 'MDH2', 'MDK', 'MEF2C', 'MEG3', 'MEIS2', 'MEOX1', 'MEST', 'METRN', 'MFAP2', 'MFAP4', 'MFGE8', 'MFSD1', 'MGAT4C', 'MGP', 'MGST1', 'MGST3', 'MIF', 'MIR4435-2HG', 'MIR503HG', 'MITF', 'MKKS', 'MKNK1', 'MKRN1', 'MLF1', 'MLLT11', 'MMP11', 'MMP2', 'MND1', 'MORF4L1', 'MPC1', 'MPC2', 'MRFAP1', 'MRPL12', 'MRPL41', 'MRPL47', 'MRPS6', 'MSN', 'MSRB2', 'MT1E', 'MT2A', 'MT3', 'MTUS1', 'MTUS2', 'MXRA5', 'MXRA8', 'MYBPC3', 'MYDGF', 'MYH10', 'MYH6', 'MYH7', 'MYH7B', 'MYH9', 'MYL12A', 'MYL12B', 'MYL2', 'MYL3', 'MYL4', 'MYL6', 'MYL7', 'MYL9', 'MYOM1', 'MYOZ2', 'MYRF', 'MZT1', 'MZT2B', 'NAA20', 'NAA38', 'NACA', 'NAP1L1', 'NARF', 'NASP', 'NAV1', 'NAXE', 'NCAM1', 'NCOA4', 'NDFIP1', 'NDN', 'NDUFA1', 'NDUFA11', 'NDUFA12', 'NDUFA13', 'NDUFA2', 'NDUFA4', 'NDUFA4L2', 'NDUFA5', 'NDUFA6', 'NDUFAB1', 'NDUFB1', 'NDUFB10', 'NDUFB11', 'NDUFB2', 'NDUFB3', 'NDUFB5', 'NDUFB7', 'NDUFB9', 'NDUFC1', 'NDUFC2', 'NDUFS5', 'NDUFS6', 'NDUFV1', 'NEAT1', 'NEBL', 'NECTIN2', 'NES', 'NEXN', 'NFIA', 'NFIB', 'NFIC', 'NFKBIA', 'NGFR', 'NID1', 'NINJ1', 'NKX2-5', 'NME1', 'NME4', 'NNAT', 'NOP10', 'NOTCH3', 'NPC2', 'NPDC1', 'NPM1', 'NPPA', 'NPPB', 'NPPC', 'NPR3', 'NR2F1', 'NR2F2', 'NREP', 'NRK', 'NRP1', 'NRP2', 'NSG1', 'NUCKS1', 'NUDT4', 'NUSAP1', 'OAZ1', 'OAZ2', 'ODC1', 'OGFRL1', 'OGN', 'OLA1', 'OLFML2B', 'OLFML3', 'OPTN', 'OSR1', 'OST4', 'OSTC', 'P4HB', 'PA2G4', 'PABPC1', 'PAFAH1B3', 'PAIP2', 'PALLD', 'PAM', 'PARK7', 'PBX1', 'PCBD1', 'PCBP2', 'PCBP4', 'PCDH17', 'PCDH18', 'PCDH7', 'PCGF5', 'PCOLCE', 'PDCD10', 'PDGFRA', 'PDGFRB', 'PDHA1', 'PDIA3', 'PDIA6', 'PDLIM1', 'PDLIM2', 'PDLIM3', 'PDLIM5', 'PDLIM7', 'PDPN', 'PEBP1', 'PEG10', 'PEG3', 'PELO', 'PENK', 'PERP', 'PFDN5', 'PFKP', 'PFN1', 'PFN2', 'PGAM1', 'PGAM2', 'PGK1', 'PGRMC1', 'PHACTR2', 'PHLDA1', 'PHPT1', 'PIM1', 'PITHD1', 'PKM', 'PKP2', 'PLA2G5', 'PLAC9', 'PLAGL1', 'PLAT', 'PLD3', 'PLEKHA1', 'PLEKHA5', 'PLIN2', 'PLK1', 'PLK2', 'PLLP', 'PLN', 'PLOD1', 'PLPP1', 'PLPP3', 'PLSCR1', 'PLTP', 'PLVAP', 'PLXDC1', 'PLXND1', 'PNISR', 'PNRC1', 'PODXL', 'POLD2', 'POLE4', 'POLR1D', 'POLR2J3', 'POLR2L', 'POMP', 'PON2', 'POPDC2', 'POPDC3', 'POSTN', 'PPA1', 'PPDPF', 'PPFIBP1', 'PPIA', 'PPIB', 'PPIC', 'PPIF', 'PPM1F', 'PPP1CC', 'PPP1R12B', 'PPP1R14A', 'PPP1R15A', 'PPP1R18', 'PPP1R1A', 'PRCP', 'PRDX1', 'PRDX2', 'PRDX3', 'PRDX4', 'PRDX5', 'PRDX6', 'PRKAG2', 'PROCR', 'PRR13', 'PRRX1', 'PRSS23', 'PRSS35',

'PSAP', 'PSIP1', 'PSMA7', 'PSMB5', 'PSMB6', 'PSMB7', 'PSMC4', 'PSMD8', 'PSMF1', 'PTGES3', 'PTMA', 'PTMS', 'PTN', 'PTPRB', 'PTPRF', 'PTTG1', 'PUF60', 'PXDN', 'PYCARD', 'QDPR', 'QKI', 'QPRT', 'QSOX1', 'R3HDM4', 'RAB11A', 'RAB11FIP1', 'RAB13', 'RAB31', 'RAB34', 'RAB5C', 'RAC1', 'RACK1', 'RAD23A', 'RALBP1', 'RAMP1', 'RAMP2', 'RAN', 'RANBP1', 'RAP1A', 'RARRES2', 'RASIP1', 'RASL11B', 'RASSF4', 'RB1', 'RBM20', 'RBM24', 'RBM3', 'RBMS3', 'RBMX', 'RBP1', 'RBPJ', 'RBX1', 'RCAN1', 'RCN1', 'RCN2', 'RCSD1', 'RDH10', 'RDX', 'REC8', 'REXO2', 'RGS19', 'RGS2', 'RGS3', 'RGS5', 'RHEB', 'RHOA', 'RHOB', 'RHOBTB3', 'RHOC', 'RHOG', 'RHOQ', 'RNASE1', 'RNASET2', 'RND3', 'RNF10', 'RNF130', 'RNF207', 'ROBO1', 'RPA3', 'RPL10', 'RPL10A', 'RPL11', 'RPL12', 'RPL13', 'RPL13A', 'RPL14', 'RPL15', 'RPL18', 'RPL18A', 'RPL19', 'RPL21', 'RPL22', 'RPL22L1', 'RPL23', 'RPL23A', 'RPL24', 'RPL26', 'RPL27', 'RPL27A', 'RPL28', 'RPL29', 'RPL3', 'RPL30', 'RPL31', 'RPL32', 'RPL34', 'RPL35', 'RPL35A', 'RPL36', 'RPL36A', 'RPL36AL', 'RPL37', 'RPL37A', 'RPL38', 'RPL39', 'RPL4', 'RPL41', 'RPL5', 'RPL6', 'RPL7', 'RPL7A', 'RPL8', 'RPL9', 'RPLP0', 'RPLP1', 'RPLP2', 'RPS10', 'RPS11', 'RPS12', 'RPS13', 'RPS14', 'RPS15', 'RPS15A', 'RPS16', 'RPS17', 'RPS18', 'RPS19', 'RPS2', 'RPS20', 'RPS21', 'RPS23', 'RPS24', 'RPS25', 'RPS26', 'RPS27', 'RPS27A', 'RPS27L', 'RPS28', 'RPS29', 'RPS3', 'RPS3A', 'RPS4X', 'RPS5', 'RPS6', 'RPS7', 'RPS8', 'RPS9', 'RPSA', 'RRAD', 'RRBP1', 'RSRP1', 'RSU1', 'RTN3', 'RTN4', 'RUNX1T1', 'RYR2', 'S100A10', 'S100A11', 'S100A13', 'S100A16', 'S100A4', 'S100A6', 'SAP18', 'SARAF', 'SAT1', 'SBSPON', 'SCAMP2', 'SCAND1', 'SC-CPDH', 'SCP2', 'SDC2', 'SDCBP', 'SDHA', 'SEC61B', 'SEC61G', 'SEC62', 'SELENBP1', 'SEMA3D', 'SEMA5A', 'SERBP1', 'SERF2', 'SERP1', 'SERPINE2', 'SERPINF1', 'SERPING1', 'SERPINH1', 'SET', 'SFPQ', 'SFRP1', 'SGK1', 'SH3BGR', 'SH3BGRL', 'SH3BGRL3', 'SH3RF2', 'SHD', 'SIAH2', 'SKP1', 'SLC16A1', 'SLC25A3', 'SLC25A39', 'SLC25A4', 'SLC25A5', 'SLC25A6', 'SLC27A3', 'SLC2A1', 'SLC2A3', 'SLC38A1', 'SLC39A8', 'SLC3A2', 'SLC40A1', 'SLC8A1', 'SLC9A3R1', 'SLC9A3R2', 'SLIT2', 'SLIT3', 'SMARCB1', 'SMC4', 'SMDT1', 'SMIM1', 'SMOC2', 'SMPX', 'SMS', 'SMTNL2', 'SMYD1', 'SNAI2', 'SNCA', 'SNHG8', 'SNRNP25', 'SNRPB', 'SNRPB2', 'SNRPD1', 'SNRPE', 'SNRPN', 'SNTA1', 'SNU13', 'SNX2', 'SNX3', 'SNX6', 'SOCS3', 'SOD1', 'SON', 'SORBS2', 'SOX4', 'SOX7', 'SOX9', 'SPARC', 'SPINT2', 'SPON2', 'SPP1', 'SPRY1', 'SPTBN1', 'SQSTM1', 'SRGN', 'SRI', 'SRP14', 'SRP9', 'SRPX', 'SRSF2', 'SRSF3', 'SRSF5', 'SRSF7', 'SSB', 'SSR2', 'SSR3', 'SSR4', 'ST13', 'STAB1', 'STK17A', 'STMN1', 'STOM', 'STRADB', 'STRAP', 'SUB1', 'SULF1', 'SULF2', 'SULT1E1', 'SUMO2', 'SUPT4H1', 'SVIL', 'SYNE2', 'SYNPO2', 'SYNPO2L', 'TAGLN', 'TAGLN2', 'TALDO1', 'TAX1BP1', 'TAX1BP3', 'TBCB', 'TBX18', 'TBX2', 'TBX3', 'TBX5', 'TCAP', 'TCEA3', 'TCEAL7', 'TCEAL9', 'TCF12', 'TCF21', 'TCF4', 'TECRL', 'TEK', 'TERF2IP', 'TESC', 'TFPI', 'TGFB1I1', 'TGFBI', 'TGM2', 'THY1', 'TIE1', 'TIMM13', 'TIMP1', 'TIMP3', 'TINAGL1', 'TK1', 'TKT', 'TLN1', 'TM4SF1', 'TMA7', 'TMBIM4', 'TMBIM6', 'TMED9', 'TMEM100', 'TMEM141', 'TMEM14B', 'TMEM14C', 'TMEM176B', 'TMEM258', 'TMEM47', 'TMEM59', 'TMEM88', 'TMEM98', 'TMOD1', 'TMSB10', 'TMSB15A', 'TMSB4X', 'TNC', 'TNFRSF12A', 'TNNC1', 'TNNI1', 'TNNI3', 'TNNT1', 'TNNT2', 'TOMM7', 'TOP1', 'TOP2A', 'TP53I11', 'TPBG', 'TPI1', 'TPM1', 'TPM2', 'TPM3', 'TPM4', 'TPP1', 'TPT1', 'TRAPPC2L', 'TRDN', 'TRIM55', 'TSC22D1', 'TSPAN13', 'TSPAN18', 'TSPAN5', 'TSPAN7', 'TSPO', 'TSTD1', 'TTC3', 'TTN', 'TUBA1A', 'TUBA1B', 'TUBA1C', 'TUBB', 'TUBB2A', 'TUBB2B', 'TUBB4B', 'TUBB6', 'TWF2', 'TXN', 'TXNDC17', 'TXNIP', 'TYMS', 'UACA', 'UBA52', 'UBAC1', 'UBB', 'UBC', 'UBE2B', 'UBE2C', 'UBE2D3', 'UBE2E3', 'UBE2T', 'UBL5', 'UCHL1', 'UCP2', 'UGP2', 'UNC45B', 'UQCC2', 'UQCR10', 'UQCR11', 'UQCRB', 'UQCRC1', 'UQCRC2', 'UQCRFS1', 'UQCRH', 'UQCRQ', 'UROD', 'UTRN', 'UXT', 'VAMP2', 'VAMP5', 'VASN', 'VASP', 'VBP1', 'VCAN', 'VDAC1', 'VDAC2', 'VDAC3', 'VGLL4', 'VIM', 'VPS28', 'VWF', 'WASF2', 'WBP11', 'WDR83OS', 'WNK1', 'WSB1', 'WWTR1', 'XIRP1', 'YBX1', 'YWHAB', 'YWHAE', 'YWHAH', 'YWHAQ', 'YWHAZ', 'ZEB2', 'ZFP36', 'ZFP36L1', 'ZFP36L2', 'ZMAT2', 'ZNF428', 'ZNF503', 'ZYX']

Supplementary Section 5: Mouse brain analysis

Cell population

In Supplementary Table 9 is the number of each cell type after preprocessing.

Cell type	#	Cell type	#	Cell type	#	Cell type	#	Cell type	#	Cell type	#
**Astro**	976	**DG**	2469	**L2/3 IT PPP**	1395	**L5 PPP**	47	**Lamp5**	4755	**SMC-Peri**	198
**CA1-ProS**	1701	**Endo**	213	**L3 IT ENT**	577	**L5 PT CTX**	1974	**Meis2**	172	**SUB-ProS**	467
**CA2**	21	**L2 IT ENTl**	179	**L3 RSP-ACA**	200	**L6 CT CTX**	6210	**Micro-PVM**	176	**Sncg**	1491
**CA3**	315	**L2 IT RHP**	375	**L4/5 IT CTX**	11522	**L6 IT CTX**	5015	**NP PPP**	150	**Sst**	5258
**CR**	32	**L2/3 IT CTX-1**	5959	**L5 IT CTX**	2934	**L6 IT ENTl**	83	**NP SUB**	257	**Sst Chodl**	268
**CT SUB**	173	**L2/3 IT CTX-2**	106	**L5 IT TPE-ENT**	338	**L6b CTX**	2213	**Oligo**	236	**VLMC**	159
**Car3**	1980	**L2/3 IT ENTl**	253	**L5 NP CTX**	2363	**L6b/CT ENT**	693	**Pvalb**	4365	**Vip**	6436

Supplementary Table 9: Cell types after preprocessing from the Allen's Mouse Brain Atlas (https://portal.brain-map.org/atlases-and-data/rnaseq/mouse-whole-cortex-and-hippocampus-smart-seq)

Predicted cell type proportions

Based on the trained model (one for all samples, based on the Allen's Mouse Brain Atlas) for Application 2, these are the predicted proportions for each cell type, shown for each tissue sample, more red and means higher proportions (zoom in to see more details).

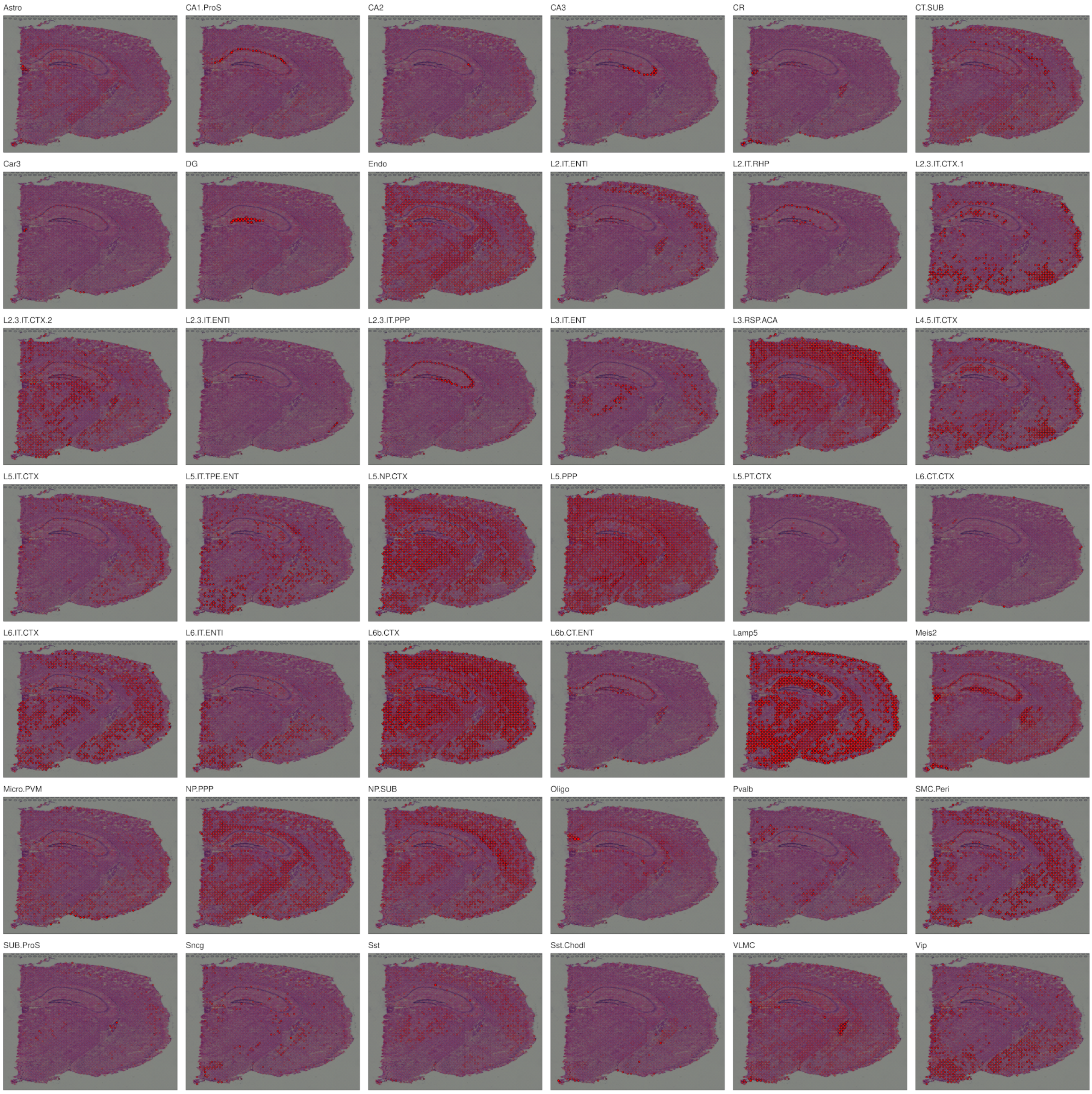

Supplementary Figure 22: Mouse brain - Sample #1

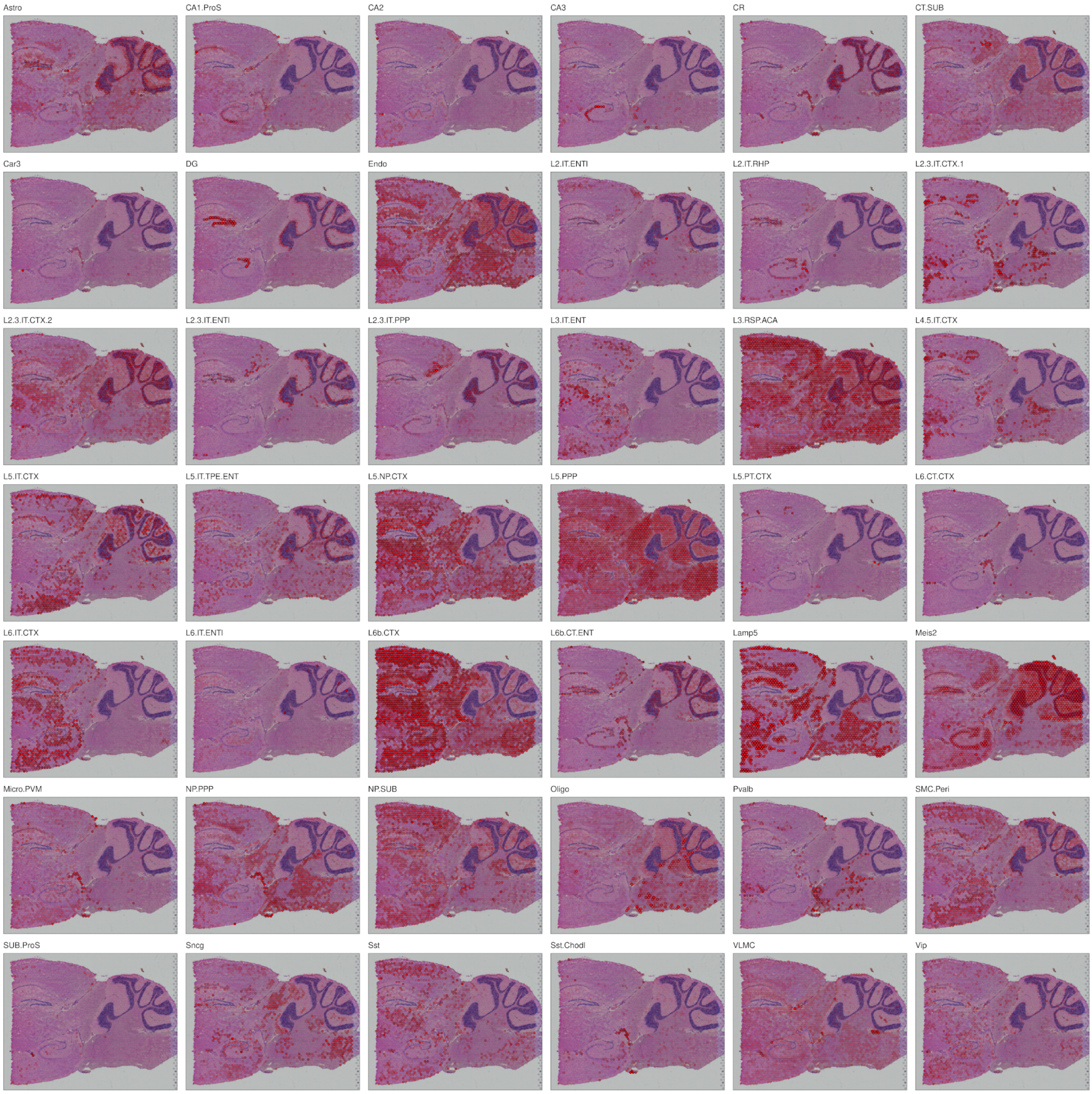

Supplementary Figure 23: Mouse brain - Sample #2

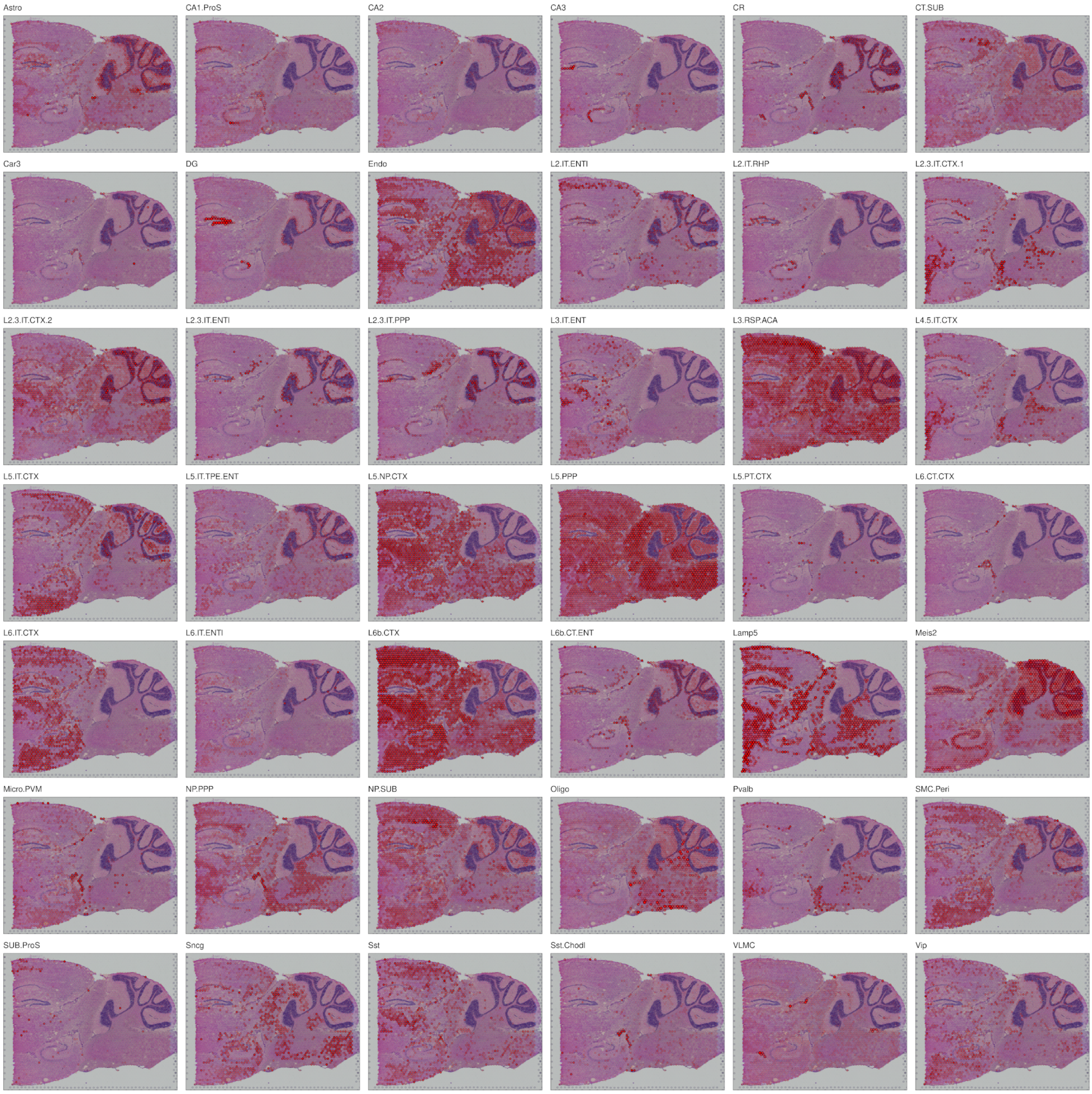

Supplementary Figure 24: Mouse brain - Sample #3

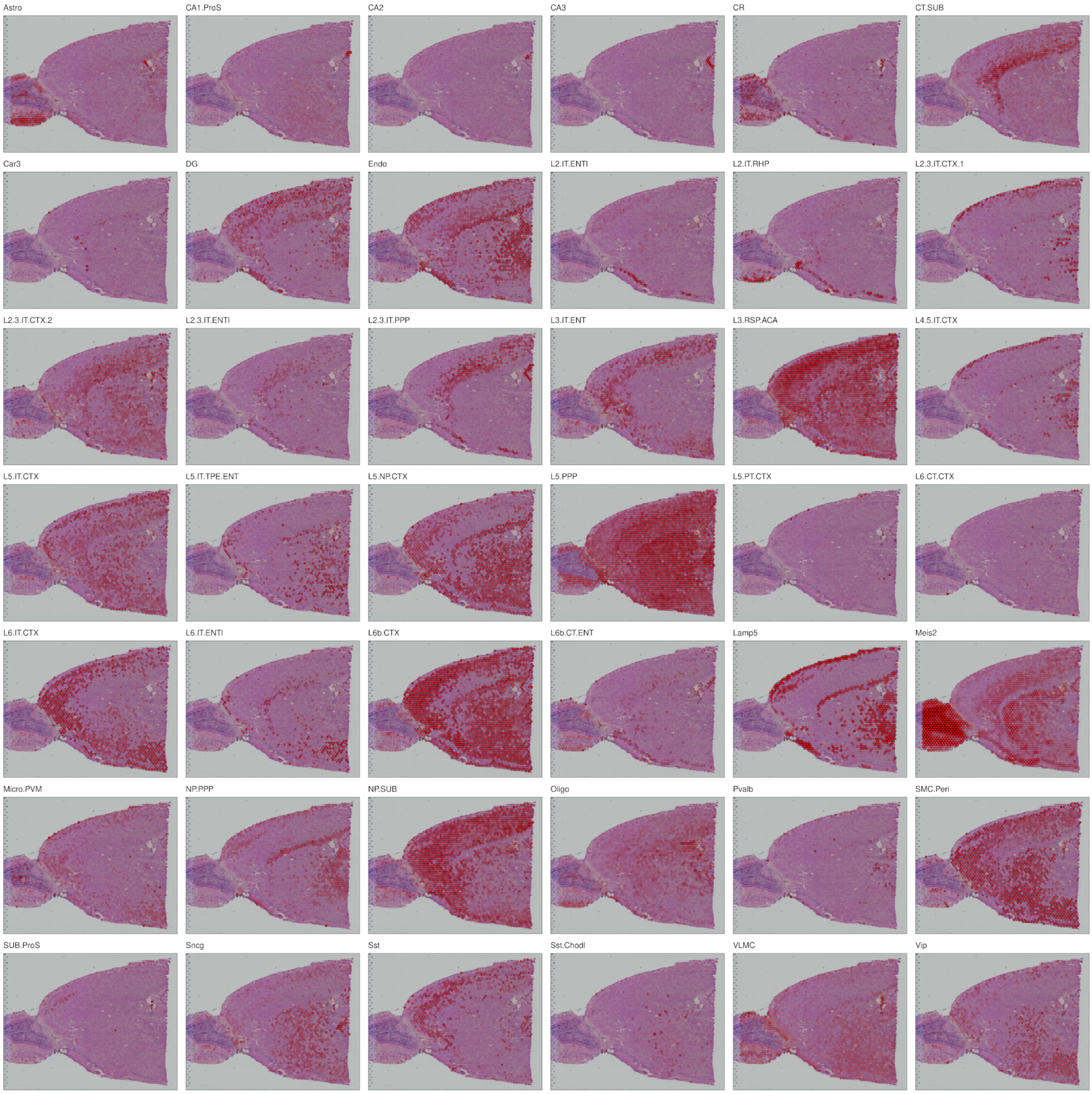

Supplementary Figure 25: Mouse brain - Sample #4

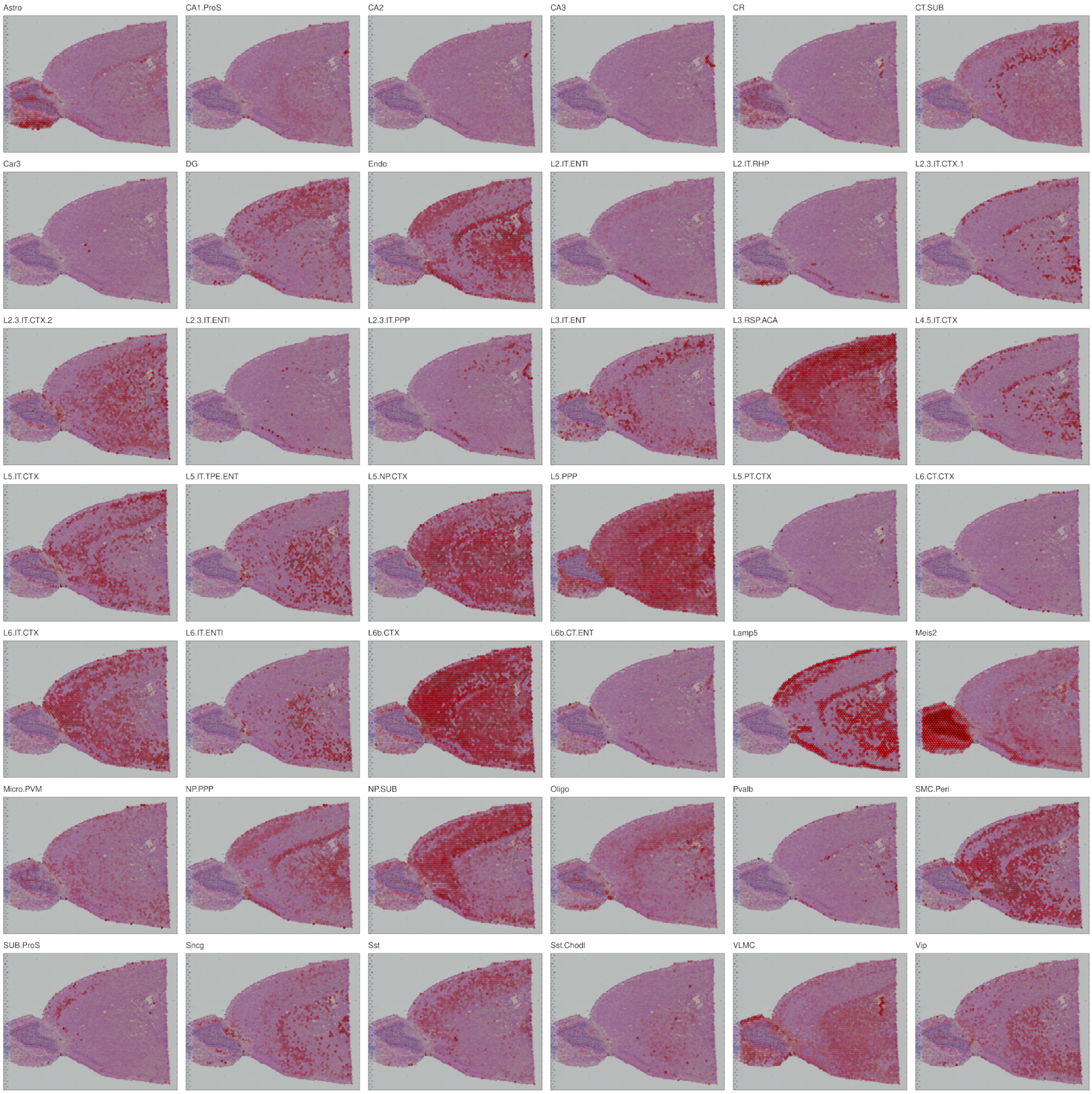

Supplementary Figure 26: Mouse brain - Sample #5

Marker genes

Below are the marker genes used (in python format, N=3557).

marker_genes = [’1110008P14Rik’, ’1110032F04Rik’, ’1190002N15Rik’, ’1500009L16Rik’, ’1500011B03Rik’, ’1500011K16Rik’, ’1700001L19Rik’, ’1700007K13Rik’, ’1700019D03Rik’, ’1700037H04Rik’, ’1700047M11Rik’, ’1810037I17Rik’, ’1810041L15Rik’, ’1810043G02Rik’, ’2010107E04Rik’, ’2010111I01Rik’, ’2010300C02Rik’, ’2610001J05Rik’, ’2810032G03Rik’, ’2810459M11Rik’, ’2900011O08Rik’, ’2900026A02Rik’, ’2900055J20Rik’, ’2900079G21Rik’, ’3110002H16Rik’, ’3110035E14Rik’, ’3300002P13Rik’, ’4930402H24Rik’, ’4930452B06Rik’, ’4930523C07Rik’, ’4931406P16Rik’, ’5031439G07Rik’, ’5330416C01Rik’, ’5330434G04Rik’, ’5730522E02Rik’, ’6330403K07Rik’, ’6330420H09Rik’, ’6430548M08Rik’, ’6430573F11Rik’, ’6530403H02Rik’, ’9330158H04Rik’, ’9330182L06Rik’, ’9530059O14Rik’, ’9630002D21Rik’, ’A230006K03Rik’, ’A330008L17Rik’, ’A330009N23Rik’, ’A330033J07Rik’, ’A330102I10Rik’, ’A630023P12Rik’, ’A730017C20Rik’, ’A830009L08Rik’, ’A830010M20Rik’, ’A830018L16Rik’, ’A930029G22Rik’, ’AF529169’, ’AI413582’, ’AI593442’, ’AU021092’, ’Aacs’, ’Abat’, ’Abca1’, ’Abca8b’, ’Abca9’, ’Abcb1a’, ’Abcc4’, ’Abcg2’, ’Abhd12’, ’Abhd17b’, ’Abhd17c’, ’Abhd3’, ’Abhd6’, ’Abi1’, ’Abi3bp’, ’Abl2’, ’Ablim1’, ’Ablim2’, ’Abracl’, ’Acat2’, ’Acer2’, ’Ache’, ’Ackr3’, ’Acot13’, ’Acot7’, ’Acrbp’, ’Acsbg1’, ’Acsl1’, ’Acsl3’, ’Acsl5’, ’Acsl6’, ’Acss1’, ’Acta1’, ’Actb’, ’Actg1’, ’Actn1’, ’Actr3’, ’Actr3b’, ’Acvr1c’, ’Acvrl1’, ’Acyp1’, ’Adam11’, ’Adam12’, ’Adam18’, ’Adam23’, ’Adamts13’, ’Adamts17’, ’Adamts2’, ’Adamts3’, ’Adamtsl2’, ’Adap1’, ’Adap2’, ’Adarb2’, ’Adcy1’, ’Adcy2’, ’Adcy8’, ’Adcy9’, ’Adcyap1’, ’Adcyap1r1’, ’Add2’, ’Add3’, ’Adk’, ’Adora1’, ’Adra1a’, ’Adra1b’, ’Adra2a’, ’Adra2c’, ’Adssl1’, ’Aebp1’, ’Aes’, ’Afap1’, ’Afap1l1’, ’Agap1’, ’Agap3’, ’Agbl4’, ’Agfg2’, ’Agpat4’, ’Ahcyl2’, ’Ahi1’, ’Ahnak’, ’Aif1’, ’Aifm3’, ’Aimp2’, ’Ajap1’, ’Ak1’, ’Ak4’, ’Ak5’, ’Akap12’, ’Akap13’, ’Akap2’, ’Akap5’, ’Akap7’, ’Akirin2’, ’Akr1b3’, ’Alcam’, ’Aldh1a1’, ’Aldh1a2’, ’Aldh1b1’, ’Aldh2’, ’Aldoa’, ’Aldoc’, ’Alg2’, ’Alox5ap’, ’Amigo2’, ’Amn’, ’Amz1’, ’Ang’, ’Angpt1’, ’Ank1’, ’Ank2’, ’Ank3’, ’Ankrd12’, ’Ankrd28’, ’Ankrd29’, ’Ankrd33b’, ’Ankrd34b’, ’Ankrd35’, ’Ankrd55’, ’Ankrd6’, ’Anks1b’, ’Ano10’, ’Ano3’, ’Ano4’, ’Anp32a’, ’Anxa1’, ’Anxa2’, ’Anxa3’, ’Anxa5’, ’Anxa6’, ’Aox3’, ’Ap1s2’, ’Ap2a2’, ’Ap2m1’, ’Ap2s1’, ’Ap3m2’, ’Apaf1’, ’Apba1’, ’Apba2’, ’Apbb2’, ’Apc’, ’Apex1’, ’Aplp1’, ’Aplp2’, ’Apod’, ’Apoe’, ’App’, ’Appl2’, ’Aprt’, ’Aqp4’, ’Araf’, ’Arap2’, ’Arc’, ’Arf3’, ’Arg2’, ’Arhgap10’, ’Arhgap12’, ’Arhgap15’, ’Arhgap20’, ’Arhgap24’, ’Arhgap25’, ’Arhgap26’, ’Arhgap29’, ’Arhgap31’, ’Arhgap32’, ’Arhgap33’, ’Arhgap39’, ’Arhgap42’, ’Arhgap44’, ’Arhgap5’, ’Arhgap6’, ’Arhgdib’, ’Arhgdig’, ’Arhgef10’, ’Arhgef25’, ’Arhgef26’, ’Arhgef28’, ’Arhgef4’, ’Arhgef6’, ’Arhgef9’, ’Arid5a’, ’Arid5b’, ’Arl15’, ’Arl3’, ’Arl4a’, ’Arl4c’, ’Arl6ip1’, ’Arnt2’, ’Arpc1a’, ’Arpc1b’, ’Arpc2’, ’Arpc3’, ’Arpc5’, ’Arpp19’, ’Arpp21’, ’Arsb’, ’Arsj’, ’Arx’, ’As3mt’, ’Asap1’, ’Asap2’, ’Ascl1’, ’Asic1’, ’Asic2’, ’Asic4’, ’Asns’, ’Aspa’, ’Asph’, ’Aspn’, ’Asrgl1’, ’Ass1’, ’Astn2’, ’Atat1’, ’Atf4’, ’Atf6b’, ’Atox1’, ’Atp10a’, ’Atp11c’, ’Atp13a4’, ’Atp1a1’, ’Atp1a2’, ’Atp1a3’, ’Atp1b1’, ’Atp1b2’, ’Atp1b3’, ’Atp2a2’, ’Atp2b1’, ’Atp2b2’, ’Atp2b4’, ’Atp5a1’, ’Atp5b’, ’Atp5c1’, ’Atp5e’, ’Atp5f1’, ’Atp5g1’, ’Atp5g3’, ’Atp5h’, ’Atp5j’, ’Atp5j2’, ’Atp5k’, ’Atp5l’, ’Atp6ap1l’, ’Atp6ap2’, ’Atp6v0b’, ’Atp6v0c’, ’Atp6v0e’, ’Atp6v1c2’, ’Atp6v1d’, ’Atp6v1e1’, ’Atp6v1f’, ’Atp6v1g2’, ’Atp9b’, ’Atpif1’, ’Atxn1’, ’Auts2’, ’Avpi1’, ’Axl’, ’B230209E15Rik’, ’B2m’, ’B3galt1’, ’B3galt2’, ’B3gat1’, ’B3gat2’, ’B3glct’, ’B4galt6’, ’B630019K06Rik’, ’BC028528’, ’BC030499’, ’BC030500’, ’Baalc’, ’Bace2’, ’Bag1’, ’Baiap2’, ’Baiap2l2’, ’Basp1’, ’Bc1’, ’Bcam’, ’Bcan’, ’Bcap29’, ’Bcas1’, ’Bcat1’, ’Bcl11a’, ’Bcl11b’, ’Bcl2l1’, ’Bcl6’, ’Bdnf’, ’Begain’, ’Bend4’, ’Bend5’, ’Bend6’, ’Bex1’, ’Bex2’, ’Bex4’, ’Bgn’, ’Bhlhe22’, ’Bhlhe40’, ’Bicc1’, ’Blcap’, ’Blnk’, ’Bloc1s1’, ’Bmp1’, ’Bmp3’, ’Bmp6’, ’Bmp7’, ’Bmper’, ’Bmpr1b’, ’Boc’, ’Bok’, ’Bphl’, ’Brd9’, ’Brinp1’, ’Brinp2’, ’Brinp3’, ’Brk1’, ’Brms1’, ’Bscl2’, ’Bsg’, ’Bsn’, ’Btbd10’, ’Btbd11’, ’Btbd17’, ’Btbd3’, ’Btbd9’, ’Btf3’, ’Btg1’, ’Btg2’, ’Btg3’, ’Bzw2’, ’C130026L21Rik’, ’C130074G19Rik’, ’C1qa’, ’C1qb’, ’C1qc’, ’C1ql2’, ’C1ql3’, ’C1qtnf2’, ’C1qtnf4’, ’C2cd2l’, ’C3ar1’, ’C530008M17Rik’, ’C730002L08Rik’, ’Cabp1’, ’Cabp7’, ’Cachd1’, ’Cacna1e’, ’Cacna1g’, ’Cacna1h’, ’Cacna2d1’, ’Cacna2d2’, ’Cacna2d3’, ’Cacnb1’, ’Cacnb2’, ’Cacnb3’, ’Cacnb4’, ’Cacng2’, ’Cacng3’, ’Cacng4’, ’Cadm1’, ’Cadm2’, ’Cadm4’, ’Cadps’, ’Cadps2’, ’Calb1’, ’Calb2’, ’Calca’, ’Cald1’, ’Calm1’, ’Calm2’, ’Caln1’, ’Caly’, ’Camk1’, ’Camk1d’, ’Camk1g’, ’Camk2a’, ’Camk2b’, ’Camk2d’, ’Camk2n1’, ’Camk2n2’, ’Camk4’, ’Camkk1’, ’Camkk2’, ’Camkv’, ’Camta1’, ’Camta2’, ’Canx’, ’Cap1’, ’Cap2’, ’Capsl’, ’Capza2’, ’Car10’, ’Car11’, ’Car12’, ’Car14’, ’Car2’, ’Car3’, ’Car4’, ’Car7’, ’Carhsp1’, ’Cartpt’, ’Casp1’, ’Cast’, ’Casz1’, ’Cat’, ’Cav2’, ’Cbln1’, ’Cbln2’, ’Cbln4’, ’Cbr3’, ’Cbs’, ’Cbwd1’, ’Ccbe1’, ’Ccdc107’, ’Ccdc12’, ’Ccdc136’, ’Ccdc141’, ’Ccdc148’, ’Ccdc155’, ’Ccdc25’, ’Ccdc3’, ’Ccdc60’, ’Ccdc65’, ’Ccdc80’, ’Ccdc85a’, ’Ccdc85b’, ’Cck’, ’Cckbr’, ’Ccl27a’, ’Ccnb1’, ’Ccnd1’, ’Ccnd2’, ’Ccne1’, ’Ccng1’, ’Ccnh’, ’Ccnjl’, ’Ccpg1’, ’Ccser1’, ’Cd14’, ’Cd164’, ’Cd200’, ’Cd274’, ’Cd302’, ’Cd34’, ’Cd37’, ’Cd44’, ’Cd47’, ’Cd53’, ’Cd59a’, ’Cd63’, ’Cd68’, ’Cd7’, ’Cd81’, ’Cd82’, ’Cd83’, ’Cd84’, ’Cd86’, ’Cd9’, ’Cdc14a’, ’Cdc40’, ’Cdc42’, ’Cdc42ep1’, ’Cdc42ep3’, ’Cdc42ep5’, ’Cdc5l’, ’Cdh10’, ’Cdh11’, ’Cdh12’, ’Cdh13’, ’Cdh18’, ’Cdh2’, ’Cdh20’, ’Cdh22’, ’Cdh4’, ’Cdh5’, ’Cdh6’, ’Cdh7’, ’Cdh8’, ’Cdh9’, ’Cdk14’, ’Cdk17’, ’Cdk18’, ’Cdk19’, ’Cdk2ap1’, ’Cdkl4’, ’Cdkl5’, ’Cdkn1a’, ’Cdo1’, ’Cds1’, ’Cdyl2’, ’Ceacam10’, ’Cebpb’, ’Cebpd’, ’Celf2’, ’Celf4’, ’Celf5’, ’Celf6’, ’Celsr2’, ’Cemip’, ’Cend1’, ’Cenpa’, ’Cenpp’, ’Cep112’, ’Cercam’, ’Cerk’, ’Cers2’, ’Cers5’, ’Cers6’, ’Cfap52’, ’Cfh’, ’Cfl1’, ’Cfl2’, ’Cflar’, ’Cgnl1’, ’Cgref1’, ’Chac1’, ’Chchd10’, ’Chchd2’, ’Chchd3’, ’Chd3’, ’Chd3os’, ’Chd5’, ’Chd7’, ’Chd9’, ’Chga’, ’Chgb’, ’Chl1’, ’Chmp1a’, ’Chn1’, ’Chn2’, ’Chodl’, ’Chrd’, ’Chrm1’, ’Chrm2’, ’Chrm3’, ’Chrna4’, ’Chrna7’, ’Chst1’, ’Chst11’, ’Chst12’, ’Chst2’, ’Chst7’, ’Chst8’, ’Chst9’, ’Ciapin1’, ’Ciart’, ’Cib2’, ’Cidea’, ’Cinp’, ’Cisd1’, ’Cit’, ’Cited2’, ’Cited4’, ’Ckb’, ’Ckmt1’, ’Clcn2’, ’Cldn10’, ’Cldn11’, ’Cldn22’, ’Cldn5’, ’Cldnd1’, ’Clec2l’, ’Clic1’, ’Clic4’, ’Clic5’, ’Clip4’, ’Clk1’, ’Clmn’, ’Clmp’, ’Clpp’, ’Clstn1’, ’Clstn2’, ’Clstn3’, ’Clta’, ’Clu’, ’Clvs2’, ’Clybl’, ’Cmas’, ’Cmbl’, ’Cmc2’, ’Cmip’, ’Cmtm5’, ’Cmtm7’, ’Cnbp’, ’Cnih2’, ’Cnih3’, ’Cnksr2’, ’Cnn2’, ’Cnn3’, ’Cnp’, ’Cnr1’, ’Cntfr’, ’Cntn1’, ’Cntn3’, ’Cntn4’, ’Cntn5’, ’Cntn6’, ’Cntnap2’, ’Cntnap3’, ’Cntnap4’, ’Cntnap5a’, ’Cntnap5b’, ’Cntnap5c’, ’Coa5’, ’Cobl’, ’Cobll1’, ’Coch’, ’Cog5’, ’Col11a1’, ’Col12a1’, ’Col19a1’, ’Col1a1’, ’Col1a2’, ’Col23a1’, ’Col25a1’, ’Col4a1’, ’Col4a3bp’, ’Col5a1’, ’Col6a1’, ’Colec12’, ’Comtd1’, ’Copg2’, ’Coprs’, ’Copz2’, ’Coro1a’, ’Coro6’, ’Cort’, ’Cotl1’, ’Cox17’, ’Cox4i1’, ’Cox4i2’, ’Cox5a’, ’Cox5b’, ’Cox6a1’, ’Cox6a2’, ’Cox6b1’, ’Cox6c’, ’Cox7a2’, ’Cox7b’, ’Cox7c’, ’Cox8a’, ’Cp’, ’Cpe’, ’Cpeb1’, ’Cped1’, ’Cplx1’, ’Cplx2’, ’Cplx3’, ’Cpm’, ’Cpne2’, ’Cpne4’, ’Cpne5’, ’Cpne6’, ’Cpne7’, ’Cpne8’, ’Cpne9’, ’Cpq’, ’Creg1’, ’Creg2’, ’Crh’, ’Crhbp’, ’Crhr1’, ’Crim1’, ’Crip1’, ’Crip2’, ’Crispld1’, ’Crlf1’, ’Crls1’, ’Crmp1’, ’Crtac1’, ’Crtc1’, ’Cryab’, ’Crybb1’, ’Cryl1’, ’Crym’, ’Csdc2’, ’Csf1r’, ’Csgalnact1’, ’Csmd1’, ’Csmd2’, ’Csmd3’, ’Cspg4’, ’Cspg5’, ’Csrp1’, ’Csrp2’, ’Cst3’, ’Cst6’, ’Ctbp2’, ’Ctdspl’, ’Ctgf’, ’Cthrc1’, ’Ctla2a’, ’Ctnna2’, ’Ctnnd2’, ’Ctsa’, ’Ctsb’, ’Ctsc’, ’Ctsd’, ’Ctsh’, ’Ctsl’, ’Ctss’, ’Ctsz’, ’Cttn’, ’Cttnbp2’, ’Ctxn1’, ’Ctxn2’, ’Cux1’, ’Cux2’, ’Cwh43’, ’Cx3cr1’, ’Cxcl12’, ’Cxcl14’, ’Cxcr4’, ’Cxxc4’, ’Cxxc5’, ’Cyb561’, ’Cyb5a’, ’Cyb5r1’, ’Cyba’, ’Cycs’, ’Cyfip1’, ’Cygb’, ’Cyp1b1’, ’Cyp26b1’, ’Cyp46a1’, ’Cyp4f15’, ’Cyp7b1’, ’Cyth2’, ’Cyth4’, ’Cyyr1’, ’D16Ertd472e’, ’D430041D05Rik’, ’D630023F18Rik’, ’D630045J12Rik’, ’Daam1’, ’Daam2’, ’Dab1’, ’Dab2’, ’Dach1’, ’Dach2’, ’Dact2’, ’Dact3’, ’Dad1’, ’Dap3’, ’Dapk1’, ’Dapk2’, ’Dbi’, ’Dbn1’, ’Dbndd1’, ’Dbnl’, ’Dbp’, ’Dbpht2’, ’Dbx2’, ’Dcaf12l1’, ’Dcbld1’, ’Dcc’, ’Dcdc2a’, ’Dclk1’, ’Dclk3’, ’Dcn’, ’Dctpp1’, ’Dcx’, ’Ddah1’, ’Ddah2’, ’Ddhd1’, ’Ddit3’, ’Ddit4l’, ’Ddn’, ’Ddr1’, ’Ddr2’, ’Ddx5’, ’Degs1’, ’Degs2’, ’Dennd2a’, ’Depdc7’, ’Deptor’, ’Dexi’, ’Dgat2’, ’Dgcr6’, ’Dgkb’, ’Dgkg’, ’Dgkh’, ’Dgki’, ’Dgkz’, ’Dhcr24’, ’Dhrs4’, ’Diablo’, ’Dio2’, ’Dip2a’, ’Diras1’, ’Diras2’, ’Disp2’, ’Dkk3’, ’Dkkl1’, ’Dlat’, ’Dlc1’, ’Dleu7’, ’Dlg1’, ’Dlg2’, ’Dlg5’, ’Dlgap1’, ’Dlgap3’, ’Dlgap4’, ’Dlx1’, ’Dlx1as’, ’Dlx2’, ’Dlx5’, ’Dlx6’, ’Dlx6os1’, ’Dmd’, ’Dmtn’, ’Dmwd’, ’Dnah9’, ’Dnaja4’, ’Dnajb12’, ’Dnajb5’, ’Dnajc12’, ’Dnajc15’, ’Dnajc21’, ’Dnal4’, ’Dner’, ’Dnm1’, ’Dnm3’, ’Doc2a’, ’Doc2b’, ’Dock1’, ’Dock10’, ’Dock2’, ’Dock4’, ’Dock6’, ’Dock8’, ’Dock9’, ’Dok5’, ’Dpf3’, ’Dpp10’, ’Dpp4’, ’Dpp6’, ’Dpy19l1’, ’Dpyd’, ’Dpysl3’, ’Dpysl4’, ’Dpysl5’, ’Drc1’, ’Drd1’, ’Dscam’, ’Dscaml1’, ’Dstn’, ’Dtl’, ’Dtna’, ’Dtnb’, ’Dtnbp1’, ’Dusp1’, ’Dusp10’, ’Dusp14’, ’Dusp15’, ’Dusp18’, ’Dusp26’, ’Dusp5’, ’Dusp6’, ’Dync1i1’, ’Dynll1’, ’Dynll2’, ’Dynlrb1’, ’Dzank1’, ’Ebf1’, ’Ebf3’, ’Ebpl’, ’Ece2’, ’Ech1’, ’Edil3’, ’Edn3’, ’Eef1a1’, ’Eef1e1’, ’Eef2’, ’Eepd1’, ’Efcab1’, ’Efemp1’, ’Efemp2’, ’Efhd2’, ’Efna3’, ’Efna5’, ’Efnb3’, ’Efr3a’, ’Egfem1’, ’Egfl7’, ’Egfr’, ’Egln3’, ’Egr1’, ’Egr3’, ’Egr4’, ’Ehbp1l1’, ’Ehd4’, ’Eif1’, ’Eif1b’, ’Eif4a1’, ’Eif4g3’, ’Elavl2’, ’Elavl4’, ’Elfn1’, ’Elmo1’, ’Elmo2’, ’Elmod1’, ’Eln’, ’Elovl1’, ’Elovl2’, ’Elovl5’, ’Emb’, ’Emc10’, ’Emc3’, ’Emcn’, ’Eml2’, ’Emp3’, ’Emx1’, ’Emx2’, ’Enc1’, ’Endod1’, ’Eng’, ’Enho’, ’Eno1’, ’Eno2’, ’Enox1’, ’Enpp2’, ’Enpp5’, ’Ensa’, ’Entpd1’, ’Entpd6’, ’Eogt’, ’Epas1’, ’Epha10’, ’Epha3’, ’Epha4’, ’Epha5’,

'Epha6', 'Epha7', 'Ephb1', 'Ephb2', 'Ephb6', 'Ephx1', 'Ephx2', 'Ephx4', 'Epn2', 'Epor', 'Eps8', 'Eps8l2', 'Erbb4', 'Erc2', 'Ermn', 'Esam', 'Esrrg', 'Etl4', 'Ets1', 'Ets2', 'Etv1', 'Etv5', 'Etv6', 'Eva1c', 'Evc2', 'Evi2a', 'Evl', 'Exph5', 'Ext1', 'Extl1', 'Extl3', 'Ezr', 'F11r', 'F3', 'Fa2h', 'Faah', 'Fabp5', 'Fabp7', 'Faim2', 'Fam105a', 'Fam107a', 'Fam124a', 'Fam126a', 'Fam131a', 'Fam131b', 'Fam131c', 'Fam135b', 'Fam155a', 'Fam159b', 'Fam160a1', 'Fam163b', 'Fam171a2', 'Fam180a', 'Fam183b', 'Fam184a', 'Fam189a1', 'Fam19a1', 'Fam19a2', 'Fam19a5', 'Fam20a', 'Fam20c', 'Fam210b', 'Fam212a', 'Fam212b', 'Fam213b', 'Fam3c', 'Fam46a', 'Fam49b', 'Fam69a', 'Fam81a', 'Fam84a', 'Fam84b', 'Fam89b', 'Fancd2', 'Fap', 'Far2', 'Farp1', 'Fat3', 'Fau', 'Fbln5', 'Fbxl16', 'Fbxo2', 'Fbxo21', 'Fbxo34', 'Fbxo44', 'Fbxo6', 'Fbxw7', 'Fcer1g', 'Fcgr1', 'Fcgr2b', 'Fcgr3', 'Fcgr4', 'Fcgrt', 'Fcho1', 'Fchsd2', 'Fcor', 'Fcrls', 'Fdft1', 'Fdps', 'Fermt2', 'Fermt3', 'Fez1', 'Fez2', 'Fezf2', 'Fgf1', 'Fgf10', 'Fgf11', 'Fgf12', 'Fgf13', 'Fgf14', 'Fgf9', 'Fgfr1', 'Fgfr2', 'Fgfr3', 'Fhl1', 'Fhl2', 'Fhod3', 'Fibcd1', 'Fign', 'Filip1', 'Filip1l', 'Fkbp1a', 'Fkbp1b', 'Fkbp2', 'Fkbp3', 'Fkbp7', 'Fli1', 'Flna', 'Flot1', 'Flrt1', 'Flrt3', 'Flt1', 'Flt3', 'Flywch1', 'Flywch2', 'Fmn1', 'Fmnl1', 'Fmo1', 'Fmod', 'Fn1', 'Fnbp1', 'Fnbp1l', 'Fndc1', 'Fndc3b', 'Fndc4', 'Fndc5', 'Fosl2', 'Foxc1', 'Foxg1', 'Foxn3', 'Foxo1', 'Foxo6', 'Foxp1', 'Foxp2', 'Foxp4', 'Foxq1', 'Foxs1', 'Fra10ac1', 'Fras1', 'Frat2', 'Frem1', 'Frmd4a', 'Frmd4b', 'Frmd5', 'Frmd6', 'Frmd8', 'Frmpd1', 'Frmpd4', 'Fry', 'Fscn1', 'Fstl4', 'Fstl5', 'Fth1', 'Ftl1', 'Fuca1', 'Fuom', 'Fut9', 'Fxyd1', 'Fxyd5', 'Fxyd6', 'Fxyd7', 'Fyb', 'Fyco1', 'Fzd2', 'G0s2', 'G630016G05Rik', 'Gaa', 'Gab1', 'Gabarap', 'Gabarapl1', 'Gabarapl2', 'Gabra1', 'Gabra2', 'Gabra5', 'Gabrb1', 'Gabrb2', 'Gabrb3', 'Gabrd', 'Gabrg1', 'Gabrg3', 'Gad1', 'Gad1os', 'Gad2', 'Gadd45a', 'Gal3st1', 'Gal3st3', 'Galnt10', 'Galnt13', 'Galnt14', 'Galnt16', 'Galnt18', 'Galnt9', 'Galntl6', 'Galt', 'Gap43', 'Gapdh', 'Garnl3', 'Gars', 'Gas6', 'Gas7', 'Gatm', 'Gba2', 'Gbe1', 'Gcnt4', 'Gda', 'Gdf10', 'Gdi1', 'Gdi2', 'Gdpd2', 'Gfm1', 'Gfra1', 'Gfra2', 'Gfra4', 'Gimap6', 'Ginm1', 'Gipc1', 'Gja1', 'Gja4', 'Gjb2', 'Gjb6', 'Gjc1', 'Gjc3', 'Gjd2', 'Gkap1', 'Glce', 'Gli3', 'Glis3', 'Glo1', 'Glra2', 'Glra3', 'Glrx', 'Glrx5', 'Glt8d2', 'Gltp', 'Glul', 'Gm10076', 'Gm10269', 'Gm11549', 'Gm11808', 'Gm12166', 'Gm12371', 'Gm13629', 'Gm13861', 'Gm13889', 'Gm14322', 'Gm15688', 'Gm16070', 'Gm16083', 'Gm1673', 'Gm17634', 'Gm17750', 'Gm19410', 'Gm1976', 'Gm20063', 'Gm20754', 'Gm2115', 'Gm2237', 'Gm2464', 'Gm2694', 'Gm27199', 'Gm28175', 'Gm2824', 'Gm29674', 'Gm34006', 'Gm34466', 'Gm36251', 'Gm3764', 'Gm5468', 'Gm867', 'Gm9844', 'Gmfb', 'Gmfg', 'Gmpr', 'Gnai1', 'Gnai2', 'Gnal', 'Gnao1', 'Gnas', 'Gnaz', 'Gnb4', 'Gng10', 'Gng11', 'Gng12', 'Gng13', 'Gng2', 'Gng3', 'Gng4', 'Gng5', 'Gng7', 'Gns', 'Golga7b', 'Golm1', 'Got1', 'Got2', 'Gpc1', 'Gpc4', 'Gpc5', 'Gpc6', 'Gpcpd1', 'Gpd1', 'Gpd2', 'Gper1', 'Gphn', 'Gpm6a', 'Gpm6b', 'Gpr101', 'Gpr12', 'Gpr150', 'Gpr151', 'Gpr155', 'Gpr158', 'Gpr161', 'Gpr176', 'Gpr183', 'Gpr22', 'Gpr27', 'Gpr34', 'Gpr37', 'Gpr37l1', 'Gpr45', 'Gpr6', 'Gpr83', 'Gpr88', 'Gprasp1', 'Gprasp2', 'Gprin1', 'Gprin3', 'Gpsm1', 'Gpx1', 'Gpx3', 'Gpx4', 'Gpx8', 'Gramd3', 'Grasp', 'Grb10', 'Grb14', 'Grcc10', 'Grhl1', 'Gria1', 'Gria2', 'Gria3', 'Gria4', 'Grid2', 'Grik1', 'Grik2', 'Grik3', 'Grik4', 'Grin2a', 'Grin2b', 'Grin2c', 'Grin3a', 'Grina', 'Grip1', 'Grk5', 'Grm1', 'Grm2', 'Grm3', 'Grm4', 'Grm5', 'Grm7', 'Grm8', 'Grn', 'Grp', 'Gsg1l', 'Gsk3b', 'Gsn', 'Gsta4', 'Gstm1', 'Gstm2', 'Gstm5', 'Gstm6', 'Gstm7', 'Gsto1', 'Gstp1', 'Gstt1', 'Gtf2a1l', 'Gtf2f2', 'Gucy1a2', 'Gucy2g', 'Gusb', 'Gyg', 'H1fx', 'H2-D1', 'H2-DMa', 'H2-K1', 'H2-T23', 'H2afj', 'H2afz', 'H3f3a', 'H3f3b', 'Haghl', 'Hap1', 'Hapln1', 'Hapln4', 'Hbegf', 'Hbp1', 'Hbq1b', 'Hcn1', 'Hcrtr1', 'Hcrtr2', 'Hdac9', 'Hddc2', 'Hebp1', 'Heg1', 'Hepacam', 'Herc3', 'Herc6', 'Hes1', 'Hexa', 'Hexb', 'Hey1', 'Higd1b', 'Higd2a', 'Hint1', 'Hipk2', 'Hist1h2bc', 'Hist3h2a', 'Hivep1', 'Hivep2', 'Hk2', 'Hkdc1', 'Hlf', 'Hmces', 'Hmcn1', 'Hmcn2', 'Hmgb1', 'Hmgb3', 'Hmgcr', 'Hmgcs1', 'Hmgn1', 'Hmgn2', 'Hmgn3', 'Hmgn5', 'Hnmt', 'Hnrnpa2b1', 'Hnrnpc', 'Hnrnpk', 'Homer1', 'Homer2', 'Homer3', 'Hopx', 'Hpca', 'Hpcal1', 'Hpcal4', 'Hpgd', 'Hpgds', 'Hprt', 'Hrasls', 'Hrh2', 'Hrh3', 'Hrk', 'Hs2st1', 'Hs3st1', 'Hs3st2', 'Hs3st4', 'Hs3st5', 'Hs6st2', 'Hsbp1', 'Hsd11b1', 'Hsd17b12', 'Hsp90aa1', 'Hsp90ab1', 'Hspa12a', 'Hspa1b', 'Hspa2', 'Hspa4l', 'Hspa8', 'Hspb1', 'Hspb3', 'Hspe1', 'Htr1a', 'Htr1f', 'Htr2a', 'Htr2c', 'Htr3a', 'Htr5b', 'Htr7', 'Htra1', 'Hunk', 'Iah1', 'Icam2', 'Icam5', 'Id1', 'Id2', 'Id3', 'Id4', 'Idh1', 'Idh2', 'Idh3a', 'Idnk', 'Ier2', 'Ier3', 'Ier5', 'Ifi203', 'Ifi27', 'Ifi35', 'Ifit2', 'Ifit3', 'Ifitm10', 'Ifitm2', 'Ifitm3', 'Ifnar2', 'Ifngr1', 'Ifngr2', 'Ift122', 'Ift57', 'Igf1', 'Igf1r', 'Igf2', 'Igfbp2', 'Igfbp4', 'Igfbp5', 'Igfbp6', 'Igfbp7', 'Igfn1', 'Igsf11', 'Igsf21', 'Igsf3', 'Igsf8', 'Igsf9b', 'Ikzf2', 'Il10rb', 'Il11ra1', 'Il12a', 'Il16', 'Il17ra', 'Il18', 'Il1rap', 'Il1rapl2', 'Il34', 'Ildr2', 'Ilk', 'Iltifb', 'Impact', 'Ina', 'Inafm1', 'Inafm2', 'Incenp', 'Inf2', 'Inhba', 'Inpp4b', 'Inpp5a', 'Inpp5d', 'Inpp5f', 'Inpp5j', 'Insl6', 'Ints4', 'Ints7', 'Ip6k2', 'Ipcef1', 'Ippk', 'Iqgap2', 'Irak2', 'Irf5', 'Iscu', 'Islr', 'Islr2', 'Isoc1', 'Isyna1', 'Itga1', 'Itga11', 'Itga6', 'Itga7', 'Itga8', 'Itgam', 'Itgav', 'Itgb1', 'Itgb5', 'Itgb8', 'Itih2', 'Itih5', 'Itm2a', 'Itm2b', 'Itm2c', 'Itpka', 'Itpkb', 'Itpr1', 'Ivns1abp', 'Izumo4', 'Jam2', 'Jam3', 'Jarid2', 'Jdp2', 'Jmjd1c', 'Josd2', 'Jph1', 'Jph4', 'Jsrp1', 'Jun', 'Junb', 'Jund', 'Jup', 'Kalrn', 'Kank3', 'Kazn', 'Kbtbd11', 'Kcna1', 'Kcna2', 'Kcna6', 'Kcnab1', 'Kcnab2', 'Kcnab3', 'Kcnb1', 'Kcnb2', 'Kcnc1', 'Kcnc2', 'Kcnc3', 'Kcnc4', 'Kcnd2', 'Kcnd3', 'Kcnf1', 'Kcng1', 'Kcng2', 'Kcnh1', 'Kcnh3', 'Kcnh4', 'Kcnh5', 'Kcnh7', 'Kcnh8', 'Kcnip1', 'Kcnip2', 'Kcnip3', 'Kcnip4', 'Kcnj10', 'Kcnj3', 'Kcnj4', 'Kcnj6', 'Kcnk1', 'Kcnk10', 'Kcnk2', 'Kcnk3', 'Kcnk4', 'Kcnma1', 'Kcnmb2', 'Kcnmb4', 'Kcnmb4os2', 'Kcnn2', 'Kcnn3', 'Kcnq1ot1', 'Kcnq3', 'Kcnq5', 'Kcns3', 'Kcnt2', 'Kcnv1', 'Kctd1', 'Kctd12', 'Kctd16', 'Kctd17', 'Kctd2', 'Kctd4', 'Kctd6', 'Kctd8', 'Kdr', 'Khdrbs2', 'Khdrbs3', 'Kidins220', 'Kif1a', 'Kif21a', 'Kif5c', 'Kirrel3', 'Kit', 'Kitl', 'Klf10', 'Klf12', 'Klf13', 'Klf2', 'Klf3', 'Klf4', 'Klf5', 'Klf6', 'Klf7', 'Klf9', 'Klhdc8a', 'Klhl13', 'Klhl2', 'Klhl26', 'Klhl32', 'Klk8', 'Kndc1', 'Kremen1', 'Krt1', 'Krt12', 'Krt77', 'Krt80', 'Ksr1', 'L1cam', 'Lama1', 'Lama2', 'Lamb2', 'Lamc2', 'Lamp1', 'Lamp2', 'Lamp5', 'Lancl1', 'Laptm4a', 'Laptm4b', 'Laptm5', 'Lars2', 'Lats2', 'Lbh', 'Lcat', 'Lcorl', 'Lcp1', 'Ldb2', 'Ldha', 'Ldhb', 'Ldlrad4', 'Lef1', 'Lefty1', 'Lefty2', 'Lgals1', 'Lgals8', 'Lgals9', 'Lgi2', 'Lgi3', 'Lgmn', 'Lgr5', 'Lhfp', 'Lhfpl3', 'Lhfpl5', 'Lhx1os', 'Lhx2', 'Lhx5', 'Lhx6', 'Lhx9', 'Lifr', 'Lima1', 'Limch1', 'Limd2', 'Lims1', 'Lims2', 'Lin7a', 'Lin7b', 'Lin7c', 'Lingo1', 'Lingo2', 'Lingo3', 'Lipg', 'Litaf', 'Lix1', 'Lman2', 'Lman2l', 'Lmo1', 'Lmo2', 'Lmo3', 'Lmo4', 'Lmo7', 'Lmtk2', 'Lnx1', 'Lonrf1', 'Lor', 'Lpar1', 'Lpar6', 'Lpcat2', 'Lpcat4', 'Lpgat1', 'Lpl', 'Lpp', 'Lrfn2', 'Lrp1', 'Lrp12', 'Lrp1b', 'Lrrc10b', 'Lrrc38', 'Lrrc3b', 'Lrrc4c', 'Lrrc57', 'Lrrc8b', 'Lrrk1', 'Lrrk2', 'Lrrn1', 'Lrrn2', 'Lrrn3', 'Lrrtm1', 'Lrrtm2', 'Lrrtm3', 'Lrrtm4', 'Lsamp', 'Lsp1', 'Lsr', 'Lst1', 'Ltbp4', 'Ltc4s', 'Ltk', 'Lum', 'Lurap1l', 'Luzp1', 'Luzp2', 'Lxn', 'Ly6a', 'Ly6c1', 'Ly6c2', 'Ly6e', 'Ly6g6e', 'Ly6h', 'Ly86', 'Lyn', 'Lynx1', 'Lypd1', 'Lypd6', 'Lypd6b', 'Lyst', 'Lztr1', 'Lzts1', 'Lzts3', 'Macf1', 'Macrod1', 'Maf', 'Mafb', 'Mag', 'Maged1', 'Maged2', 'Magee1', 'Magi2', 'Magi3', 'Mal', 'Mal2', 'Malat1', 'Maml2', 'Maml3', 'Man1a', 'Man1c1', 'Man2a2', 'Man2b1', 'Maob', 'Map1b', 'Map1lc3a', 'Map2', 'Map2k1', 'Map3k1', 'Map3k5', 'Map4k3', 'Map4k4', 'Map7d2', 'Mapk1', 'Mapk11', 'Mapk3', 'Mapk4', 'Mapk8', 'Mapt', 'Marc2', 'March1', 'March11', 'Mar-cks', 'Marcksl1', 'Mark2', 'Mas1', 'Masp1', 'Mast3', 'Matk', 'Matn2', 'Matr3', 'Mb21d2', 'Mbnl1', 'Mbp', 'Mcam', 'Mcc', 'Mcfd2', 'Mchr1', 'Mctp1', 'Mcu', 'Mcur1', 'Mdga1', 'Mdga2', 'Mdh1', 'Mdh2', 'Mdk', 'Me1', 'Me3', 'Mecom', 'Med10', 'Med16', 'Medag', 'Mef2a', 'Mef2c', 'Meg3', 'Megf10', 'Megf9', 'Meis2', 'Mertk', 'Mest', 'Metap1d', 'Metrn', 'Metrnl', 'Mfge8', 'Mfsd2a', 'Mfsd6', 'Mgat4b', 'Mgat4c', 'Mgat5', 'Mgat5b', 'Mgll', 'Mgp', 'Mgst3', 'Miat', 'Mical2', 'Mid1ip1', 'Mif', 'Minos1', 'Mkl1', 'Mkl2', 'Mkx', 'Mlc1', 'Mlf2', 'Mlip', 'Mmd', 'Mmd2', 'Mmel1', 'Mmp11', 'Mmp16', 'Mmp17', 'Mndal', 'Mobp', 'Mog', 'Morf4l1', 'Morf4l2', 'Mpc1', 'Mpc2', 'Mpdz', 'Mpi', 'Mpp1', 'Mpp3', 'Mpp6', 'Mpp7', 'Mpped1', 'Mpped2', 'Mprip', 'Mpv17l', 'Mpv17l2', 'Mpzl1', 'Mrap2', 'Mras', 'Mrfap1', 'Mrps21', 'Mrps30', 'Mrps5', 'Msi2', 'Msmo1', 'Msn', 'Msra', 'Msrb2', 'Mt1', 'Mt2', 'Mt3', 'Mtfp1', 'Mtss1', 'Mtss1l', 'Mturn', 'Mtus1', 'Mustn1', 'Mxra7', 'Mxra8', 'Myadm', 'Mycbp2', 'Mycl', 'Myh14', 'Myh3', 'Myh7', 'Myh9', 'Myl12a', 'Myl12b', 'Myl4', 'Myl6', 'Myl9', 'Mylip', 'Mylk', 'Myo10', 'Myo16', 'Myo1b', 'Myo1e', 'Myo3b', 'Myo5a', 'Myo5b', 'Myo6', 'Myrf', 'Myt1l', 'Myzap', 'N4bp2l1', 'N6amt1', 'Nab1', 'Nabp1', 'Nacc2', 'Nagk', 'Nap1l3', 'Nap1l5', 'Napa', 'Napb', 'Napepld', 'Nars', 'Nat14', 'Nat8', 'Nav1', 'Nav2', 'Nbea', 'Nbl1', 'Ncald', 'Ncam1', 'Ncam2', 'Ncaph', 'Ncdn', 'Nceh1', 'Nckap1', 'Nckap1l', 'Ndfip1', 'Ndnf', 'Ndrg1', 'Ndrg2', 'Ndrg3', 'Ndrg4', 'Ndst3', 'Ndst4', 'Ndufa1', 'Ndufa11', 'Ndufa12', 'Ndufa13', 'Ndufa2', 'Ndufa3', 'Ndufa4', 'Ndufa4l2', 'Ndufa5', 'Ndufa6', 'Ndufa7', 'Ndufab1', 'Ndufb10', 'Ndufb11', 'Ndufb2', 'Ndufb3', 'Ndufb4', 'Ndufb8', 'Ndufb9', 'Ndufc1', 'Ndufc2', 'Ndufs5', 'Ndufs6', 'Ndufs7', 'Ndufv2', 'Neat1', 'Nebl', 'Necab1', 'Necab2', 'Necab3', 'Nedd8', 'Nefh', 'Nefl', 'Nefm', 'Negr1', 'Nek6', 'Nek7', 'Nell1', 'Nell2', 'Nenf', 'Neo1', 'Neto1', 'Neto2', 'Neu2', 'Neurl1a', 'Neurl1b', 'Neurod1', 'Neurod2', 'Neurod6', 'Nf2', 'Nfe2l2', 'Nfia', 'Nfib', 'Nfix', 'Nfkbia', 'Ngef', 'Ngf', 'Nhlh2', 'Nhp2', 'Nhs', 'Nhsl1', 'Nhsl2', 'Nin', 'Ninj1', 'Ninj2', 'Nipa1', 'Nipal3', 'Nipsnap3b', 'Nkain2', 'Nkain3', 'Nkain4', 'Nkd1', 'Nkiras1', 'Nkx6-2', 'Nlgn1', 'Nlk', 'Nme1', 'Nmnat2', 'Nnat', 'Nog', 'Nop10', 'Nos1', 'Nos1ap', 'Nostrin', 'Notch3', 'Nov', 'Nova1', 'Npas1', 'Npas2', 'Npas3', 'Npc1', 'Npc2', 'Nppc', 'Npr3', 'Npsr1', 'Nptn', 'Nptx1', 'Nptx2', 'Nptxr', 'Npy', 'Npy1r', 'Npy2r', 'Nr2e1', 'Nr2f2', 'Nr3c2', 'Nr4a1', 'Nr4a2', 'Nr4a3',

'Nrep', 'Nrg1', 'Nrg3', 'Nrgn', 'Nrip2', 'Nrip3', 'Nrn1', 'Nrp1', 'Nrp2', 'Nrros', 'Nrsn1', 'Nrsn2', 'Nrtn', 'Nrxn1', 'Nrxn3', 'Nsdhl', 'Nsf', 'Nsg1', 'Nsg2', 'Nsmf', 'Nt5dc2', 'Nt5dc3', 'Ntf3', 'Ntm', 'Ntn1', 'Ntn5', 'Ntng1', 'Ntng2', 'Ntrk2', 'Ntrk3', 'Nts', 'Ntsr2', 'Nuak1', 'Nucb2', 'Nudt11', 'Nudt17', 'Nudt4', 'Numb', 'Numbl', 'Nupr1', 'Nwd1', 'Nwd2', 'Nxph1', 'Nxph2', 'Nxph3', 'Nxph4', 'Nyap2', 'Oaf', 'Oaz1', 'Oaz2', 'Ociad2', 'Ocln', 'Ocm', 'Ogfrl1', 'Ogn', 'Ogt', 'Olfm1', 'Olfm2', 'Olfm3', 'Olfml2b', 'Olfml3', 'Olig1', 'Olig2', 'Oma1', 'Opalin', 'Opcml', 'Ophn1', 'Opn3', 'Oprk1', 'Orai2', 'Orai3', 'Osbp2', 'Osbpl10', 'Osbpl1a', 'Osbpl6', 'Osbpl8', 'Oscp1', 'Oser1', 'Osr1', 'Ost4', 'Ostf1', 'Otof', 'Otub1', 'Otub2', 'Otud7b', 'Ovol2', 'Oxr1', 'Oxtr', 'P2rx4', 'P2ry12', 'P2ry13', 'P2ry14', 'P2ry6', 'P4ha1', 'Pacs2', 'Pacsin1', 'Pacsin2', 'Pacsin3', 'Pafah1b3', 'Paip2', 'Pak1', 'Pak3', 'Pak6', 'Pak7', 'Palm2', 'Palmd', 'Pam', 'Pamr1', 'Pantr1', 'Paqr5', 'Paqr8', 'Parm1', 'Parp1', 'Parp8', 'Pbx1', 'Pbx3', 'Pcbd1', 'Pcbd2', 'Pcbp3', 'Pcca', 'Pcdh11x', 'Pcdh15', 'Pcdh17', 'Pcdh18', 'Pcdh19', 'Pcdh20', 'Pcdh7', 'Pcdh8', 'Pcdh9', 'Pcgf5', 'Pclo', 'Pcnt', 'Pcolce', 'Pcp4', 'Pcp4l1', 'Pcsk1', 'Pcsk1n', 'Pcsk2', 'Pcsk4', 'Pcsk5', 'Pdcd5', 'Pde11a', 'Pde1a', 'Pde1c', 'Pde2a', 'Pde3a', 'Pde3b', 'Pde4a', 'Pde4b', 'Pde4d', 'Pde5a', 'Pde7b', 'Pde9a', 'Pdgfra', 'Pdgfrb', 'Pdia5', 'Pdk1', 'Pdk2', 'Pdlim1', 'Pdlim2', 'Pdlim5', 'Pdlim7', 'Pdp1', 'Pdrg1', 'Pdxk', 'Pdzd2', 'Pdzrn3', 'Pdzrn4', 'Pea15a', 'Pebp1', 'Pecam1', 'Peg3', 'Peli1', 'Penk', 'Pepd', 'Per3', 'Perp', 'Pex5l', 'Pfdn1', 'Pfkl', 'Pfkp', 'Pfn1', 'Pfn2', 'Pgap1', 'Pgbd5', 'Pglyrp1', 'Pgm2', 'Pgm2l1', 'Pgrmc1', 'Phactr1', 'Phactr2', 'Phb', 'Phf20', 'Phgdh', 'Phka1', 'Phkg1', 'Phlda1', 'Phlda3', 'Phldb1', 'Phldb2', 'Phlpp1', 'Phyh', 'Phyhd1', 'Phyhip', 'Phyhipl', 'Picalm', 'Pid1', 'Pih1d1', 'Pik3ip1', 'Pip4k2a', 'Pip4k2c', 'Pip5k1b', 'Pip5k1c', 'Pip5kl1', 'Pitpnc1', 'Pitpnm2', 'Pkib', 'Pkig', 'Pkp2', 'Pkp4', 'Pla2g16', 'Pla2g7', 'Plat', 'Plcb1', 'Plcb4', 'Plcd4', 'Plce1', 'Plch1', 'Plch2', 'Plcl1', 'Plcl2', 'Plcxd2', 'Plcxd3', 'Pld3', 'Pld4', 'Pld5', 'Plek', 'Plekha2', 'Plekha7', 'Plekhb1', 'Plekho1', 'Plk2', 'Plk5', 'Pllp', 'Plp1', 'Plp2', 'Pls1', 'Pls3', 'Pltp', 'Plxdc1', 'Plxdc2', 'Plxna1', 'Plxna2', 'Plxna4', 'Plxnb3', 'Plxnc1', 'Plxnd1', 'Pmepa1', 'Pmm1', 'Pmp22', 'Pnck', 'Pnisr', 'Pnkd', 'Pnma2', 'Pnmal2', 'Pnoc', 'Pnp', 'Pnpo', 'Podxl', 'Podxl2', 'Pole4', 'Polr2h', 'Polr3k', 'Pon2', 'Pop5', 'Popdc3', 'Por', 'Porcn', 'Pou3f1', 'Pou3f2', 'Pou6f2', 'Pparg', 'Ppargc1a', 'Ppfia2', 'Ppfibp1', 'Ppia', 'Ppif', 'Ppm1e', 'Ppm1l', 'Ppp1cc', 'Ppp1r12a', 'Ppp1r14a', 'Ppp1r14b', 'Ppp1r14c', 'Ppp1r16b', 'Ppp1r18', 'Ppp1r1a', 'Ppp1r1b', 'Ppp1r2', 'Ppp1r7', 'Ppp2r2b', 'Ppp2r2c', 'Ppp2r3a', 'Ppp3ca', 'Ppp3r1', 'Ppp4r4', 'Pptc7', 'Pradc1', 'Prdm16', 'Prdm5', 'Prdm8', 'Prdx1', 'Prdx2', 'Prdx4', 'Prdx5', 'Prdx6', 'Prelp', 'Prepl', 'Prex1', 'Prex2', 'Prickle1', 'Prickle2', 'Prkacb', 'Prkag2', 'Prkar1a', 'Prkar2b', 'Prkca', 'Prkcb', 'Prkcd', 'Prkce', 'Prkcg', 'Prkch', 'Prkcq', 'Prkg1', 'Prkg2', 'Prmt2', 'Prmt5', 'Prmt8', 'Prnp', 'Prodh', 'Prom1', 'Prox1', 'Prph', 'Prr13', 'Prr16', 'Prr36', 'Prr5', 'Prr5l', 'Prrg1', 'Prrt1', 'Prrt2', 'Prrt3', 'Prss12', 'Prss22', 'Prss23', 'Prss35', 'Prune2', 'Psat1', 'Psd', 'Psd3', 'Psip1', 'Psma3', 'Psmb1', 'Psme1', 'Psph', 'Psrc1', 'Ptchd1', 'Ptchd4', 'Pter', 'Ptgds', 'Ptger3', 'Ptgfrn', 'Pthlh', 'Ptk2', 'Ptk2b', 'Ptma', 'Ptn', 'Ptp4a3', 'Ptpn13', 'Ptpn14', 'Ptpn18', 'Ptpn3', 'Ptpn5', 'Ptprb', 'Ptprc', 'Ptprd', 'Ptpre', 'Ptprf', 'Ptprg', 'Ptprk', 'Ptprm', 'Ptprn2', 'Ptpro', 'Ptprr', 'Ptprs', 'Ptprt', 'Ptpru', 'Ptprz1', 'Pvalb', 'Pvt1', 'Pwwp2b', 'Pxylp1', 'Pycr2', 'Pygm', 'Pyurf', 'Qdpr', 'Qk', 'Qpct', 'Qrfpr', 'R3hdm1', 'R3hdm2', 'R3hdm4', 'Rab15', 'Rab1b', 'Rab26', 'Rab27b', 'Rab2a', 'Rab31', 'Rab38', 'Rab3a', 'Rab3b', 'Rab3c', 'Rab3il1', 'Rab3ip', 'Rab40b', 'Rab6a', 'Rab6b', 'Rab8b', 'Rabac1', 'Rabggta', 'Rac1', 'Rac3', 'Rai14', 'Ralbp1', 'Ralgapa2', 'Ralgds', 'Ralgps2', 'Ralyl', 'Ramp1', 'Ramp2', 'Ramp3', 'Ranbp3l', 'Rangap1', 'Rap1a', 'Rap1b', 'Rap1gap', 'Rap1gap2', 'Rap1gds1', 'Rap2a', 'Rap2b', 'Rapgef3', 'Rapgef4', 'Rapgef5', 'Rarres2', 'Rasa3', 'Rasal1', 'Rasd1', 'Rasgef1a', 'Rasgef1b', 'Rasgef1c', 'Rasgrf1', 'Rasgrf2', 'Rasgrp1', 'Rasgrp2', 'Rasgrp3', 'Rasl10a', 'Rasl11a', 'Rasl11b', 'Rasl12', 'Rassf2', 'Raver2', 'Rb1', 'Rbfox1', 'Rbfox3', 'Rbm24', 'Rbm39', 'Rbm47', 'Rbms1', 'Rbms3', 'Rbp1', 'Rbp4', 'Rbpms', 'Rbx1', 'Rcan2', 'Rcan3', 'Rcn1', 'Rcn3', 'Rdx', 'Reep3', 'Reep5', 'Rell1', 'Rell2', 'Reln', 'Rem2', 'Rere', 'Rerg', 'Resp18', 'Rfc5', 'Rfk', 'Rfx3', 'Rgcc', 'Rgl1', 'Rgma', 'Rgmb', 'Rgs10', 'Rgs12', 'Rgs14', 'Rgs17', 'Rgs19', 'Rgs2', 'Rgs20', 'Rgs3', 'Rgs4', 'Rgs5', 'Rgs6', 'Rgs7', 'Rgs7bp', 'Rgs8', 'Rgs9', 'Rhbdl3', 'Rhoa', 'Rhob', 'Rhoc', 'Rhog', 'Rhoj', 'Rhou', 'Rhpn2', 'Rilpl1', 'Rimbp2', 'Rims2', 'Rims3', 'Rin2', 'Rit2', 'Rln1', 'Rmnd5b', 'Rmst', 'Rnase4', 'Rnasek', 'Rnaset2a', 'Rnaset2b', 'Rnd1', 'Rnd2', 'Rnf112', 'Rnf128', 'Rnf13', 'Rnf130', 'Rnf144b', 'Rnf150', 'Rnf152', 'Rnf165', 'Rnf182', 'Rnh1', 'Rnpep', 'Robo1', 'Robo2', 'Rogdi', 'Romo1', 'Ror1', 'Rora', 'Rorb', 'Rph3a', 'Rpl10', 'Rpl13', 'Rpl13a', 'Rpl18', 'Rpl18a', 'Rpl19', 'Rpl21', 'Rpl22l1', 'Rpl23a', 'Rpl24', 'Rpl27', 'Rpl3', 'Rpl31', 'Rpl32', 'Rpl35', 'Rpl35a', 'Rpl36', 'Rpl37', 'Rpl37a', 'Rpl38', 'Rpl4', 'Rpl41', 'Rpl7', 'Rpl8', 'Rpl9', 'Rplp0', 'Rplp1', 'Rplp2', 'Rpp25', 'Rprm', 'Rprml', 'Rps11', 'Rps13', 'Rps14', 'Rps19', 'Rps2', 'Rps21', 'Rps23', 'Rps24', 'Rps25', 'Rps27', 'Rps27a', 'Rps27l', 'Rps27rt', 'Rps28', 'Rps29', 'Rps3', 'Rps5', 'Rps6ka2', 'Rps7', 'Rps8', 'Rps9', 'Rragb', 'Rragd', 'Rras', 'Rrbp1', 'Rreb1', 'Rsph3a', 'Rsph9', 'Rspo1', 'Rspo2', 'Rspo3', 'Rsrp1', 'Rsu1', 'Rtn1', 'Rtn2', 'Rtn3', 'Rtn4', 'Rtn4r', 'Rtn4rl1', 'Rtn4rl2', 'Runx1', 'Runx1t1', 'Rwdd2a', 'Rwdd3', 'Rxfp1', 'Rxrg', 'Ryr1', 'Ryr2', 'Ryr3', 'S100a1', 'S100a10', 'S100a11', 'S100a13', 'S100a16', 'S100a6', 'S100b', 'S1pr1', 'Sac3d1', 'Sall1', 'Samd5', 'Sars', 'Sash1', 'Sat1', 'Satb1', 'Satb2', 'Sc5d', 'Scai', 'Scamp2', 'Sccpdh', 'Scd2', 'Scg2', 'Scg3', 'Scg5', 'Scgn', 'Schip1', 'Scml4', 'Scn1a', 'Scn1b', 'Scn3b', 'Scn4b', 'Scn9a', 'Scnn1a', 'Scoc', 'Scrg1', 'Scrn1', 'Scube1', 'Sdc2', 'Sdc3', 'Sdcbp', 'Sdhb', 'Sdhc', 'Sdk1', 'Sdk2', 'Sdr39u1', 'Sebox', 'Sec11c', 'Sec14l1', 'Sec14l2', 'Sec61g', 'Sec62', 'Sel1l3', 'Selplg', 'Sema3a', 'Sema3c', 'Sema3e', 'Sema4a', 'Sema5a', 'Sema5b', 'Sema6a', 'Sema6d', 'Senp7', 'Sept11', 'Sept2', 'Sept3', 'Sept4', 'Sept5', 'Sept7', 'Sept8', 'Sept9', 'Serf2', 'Sergef', 'Serinc1', 'Serinc2', 'Serinc3', 'Serinc5', 'Serp2', 'Serpina3n', 'Serpinb9', 'Serpine2', 'Serpinf1', 'Serping1', 'Serpinh1', 'Serpini1', 'Sertm1', 'Sestd1', 'Sez6', 'Sez6l', 'Sfrp1', 'Sft2d1', 'Sfxn1', 'Sfxn5', 'Sgcd', 'Sgcz', 'Sgip1', 'Sgms1', 'Sgpp2', 'Sh3bgrl', 'Sh3bgrl3', 'Sh3bp5', 'Sh3d19', 'Sh3gl2', 'Sh3gl3', 'Sh3glb1', 'Sh3kbp1', 'Sh3rf1', 'Sh3rf3', 'Shank1', 'Shank2', 'Shb', 'Shc2', 'Shd', 'Shf', 'Shisa4', 'Shisa5', 'Shisa6', 'Shisa8', 'Shisa9', 'Shroom2', 'Siah1a', 'Sidt1', 'Siglech', 'Sigmar1', 'Sik3', 'Sipa1l1', 'Sipa1l3', 'Sirpa', 'Sirt2', 'Sirt3', 'Skap1', 'Skap2', 'Skp1a', 'Sla', 'Sla2', 'Slain1', 'Slbp', 'Slc12a2', 'Slc13a3', 'Slc13a4', 'Slc15a2', 'Slc16a12', 'Slc16a2', 'Slc17a6', 'Slc17a7', 'Slc17a8', 'Slc19a1', 'Slc1a1', 'Slc1a2', 'Slc1a3', 'Slc20a1', 'Slc22a17', 'Slc22a6', 'Slc22a8', 'Slc24a2', 'Slc24a3', 'Slc24a4', 'Slc25a1', 'Slc25a17', 'Slc25a18', 'Slc25a22', 'Slc25a3', 'Slc25a4', 'Slc26a4', 'Slc27a1', 'Slc27a2', 'Slc29a1', 'Slc2a1', 'Slc2a13', 'Slc2a6', 'Slc30a3', 'Slc30a4', 'Slc32a1', 'Slc35c2', 'Slc35f1', 'Slc35f3', 'Slc35f4', 'Slc35g2', 'Slc36a1', 'Slc38a1', 'Slc38a11', 'Slc38a3', 'Slc39a10', 'Slc39a11', 'Slc39a12', 'Slc39a6', 'Slc3a2', 'Slc40a1', 'Slc41a1', 'Slc44a1', 'Slc44a5', 'Slc4a10', 'Slc4a3', 'Slc4a4', 'Slc4a7', 'Slc5a5', 'Slc6a1', 'Slc6a11', 'Slc6a13', 'Slc6a17', 'Slc6a20a', 'Slc6a6', 'Slc6a7', 'Slc7a1', 'Slc7a10', 'Slc7a11', 'Slc7a2', 'Slc7a3', 'Slc7a4', 'Slc7a5', 'Slc8a1', 'Slc8a2', 'Slc9a2', 'Slc9a3r1', 'Slc9a3r2', 'Slc9a6', 'Slc9a9', 'Slco1a4', 'Slco1c1', 'Slco2b1', 'Slco3a1', 'Slit1', 'Slit2', 'Slit3', 'Slitrk1', 'Slk', 'Smad2', 'Smad3', 'Smap1', 'Smarcd3', 'Smc2', 'Smco3', 'Smim14', 'Smim3', 'Smim4', 'Smoc2', 'Smpd3', 'Smpdl3a', 'Smyd2', 'Smyd3', 'Snap25', 'Snap47', 'Snap91', 'Snca', 'Sncb', 'Sncg', 'Snhg11', 'Snhg18', 'Snrpb', 'Snrpn', 'Snta1', 'Sntb1', 'Sntb2', 'Sntg1', 'Sntg2', 'Snx10', 'Snx25', 'Snx3', 'Snx5', 'Snx7', 'Snx8', 'Sobp', 'Socs2', 'Sod3', 'Son', 'Sorbs1', 'Sorbs2', 'Sorcs1', 'Sorcs2', 'Sorcs3', 'Sorl1', 'Sort1', 'Sowaha', 'Sox1', 'Sox10', 'Sox11', 'Sox17', 'Sox18', 'Sox2', 'Sox5', 'Sox6', 'Sox8', 'Sox9', 'Sp100', 'Sp8', 'Sp9', 'Spag9', 'Sparc', 'Sparcl1', 'Spata2l', 'Spats2l', 'Spcs1', 'Speg', 'Sphkap', 'Spi1', 'Spin2c', 'Spink8', 'Spint2', 'Spns2', 'Spock1', 'Spock2', 'Spock3', 'Spon1', 'Spop', 'Spr', 'Sprn', 'Spsb1', 'Sptb', 'Sptbn1', 'Sqle', 'Sqstm1', 'Srcin1', 'Srebf1', 'Srgap1', 'Srgap2', 'Srgap3', 'Srgn', 'Srp14', 'Srrm2', 'Srrm4', 'Srsf5', 'Srsf9', 'Ssbp2', 'Ssbp3', 'Ssbp4', 'Ssh2', 'Sst', 'Sstr2', 'St18', 'St3gal1', 'St3gal4', 'St3gal5', 'St3gal6', 'St6gal1', 'St6gal2', 'St6galnac2', 'St6galnac3', 'St6galnac5', 'St8sia1', 'St8sia3', 'St8sia4', 'St8sia5', 'Stab1', 'Stac', 'Stac2', 'Stambpl1', 'Stard10', 'Stard13', 'Stard5', 'Stard7', 'Stard8', 'Stim2', 'Stk26', 'Stk32c', 'Stmn1', 'Stmn2', 'Stmn3', 'Stmn4', 'Stox2', 'Stpg1', 'Strbp', 'Strip2', 'Strn4', 'Stx1a', 'Stxbp1', 'Stxbp2', 'Stxbp5l', 'Stxbp6', 'Sub1', 'Sulf1', 'Sulf2', 'Sult2b1', 'Sumo1', 'Sumo2', 'Supt6', 'Susd4', 'Sv2a', 'Sv2b', 'Sv2c', 'Svil', 'Syce2', 'Syn1', 'Syn2', 'Syn3', 'Syndig1', 'Syndig1l', 'Syne1', 'Syngr3', 'Synj2', 'Synm', 'Synpo', 'Synpr', 'Syp', 'Syt1', 'Syt10', 'Syt11', 'Syt12', 'Syt13', 'Syt16', 'Syt17', 'Syt2', 'Syt3', 'Syt4', 'Syt5', 'Syt6', 'Syt7', 'Sytl2', 'Tac1', 'Tacr1', 'Tagln2', 'Tagln3', 'Taldo1', 'Tanc1', 'Tanc2', 'Taok3', 'Tarsl2', 'Tbata', 'Tbc1d23', 'Tbc1d30', 'Tbc1d4', 'Tbr1', 'Tbx18', 'Tbx2', 'Tbx3', 'Tbxas1', 'Tcap', 'Tceal1', 'Tceal3', 'Tceal5', 'Tceal8', 'Tcerg1l', 'Tcf12', 'Tcf4', 'Tcf7l2', 'Tcn2', 'Tcp11l1', 'Tecr', 'Tek', 'Tekt5', 'Tenm1', 'Tenm2', 'Tenm3', 'Tenm4', 'Tesc', 'Tesk1', 'Tex264', 'Tfap2b', 'Tfpi', 'Tgfb1', 'Tgfb1i1', 'Tgfb2', 'Tgfbr1', 'Tgfbr2', 'Tgfbr3', 'Thpo', 'Thra', 'Thrb', 'Thrsp', 'Thsd4', 'Thsd7a', 'Thsd7b', 'Tiam1', 'Tiam2', 'Timm8a1', 'Timp2', 'Timp3', 'Timp4', 'Tinagl1', 'Tjp1', 'Tjp2', 'Tlcd1', 'Tle1', 'Tle4', 'Tlk1', 'Tll1', 'Tm4sf1', 'Tm6sf1', 'Tma7', 'Tmbim1', 'Tmbim6', 'Tmcc3', 'Tmco5', 'Tmed10', 'Tmeff1',

’Tmeff2’, ’Tmem100’, ’Tmem108’, ’Tmem125’, ’Tmem130’, ’Tmem132a’, ’Tmem132c’, ’Tmem132d’, ’Tmem132e’, ’Tmem141’, ’Tmem145’, ’Tmem150c’, ’Tmem151a’, ’Tmem158’, ’Tmem159’, ’Tmem163’, ’Tmem176b’, ’Tmem178’, ’Tmem191c’, ’Tmem196’, ’Tmem200a’, ’Tmem204’, ’Tmem215’, ’Tmem232’, ’Tmem243’, ’Tmem255a’, ’Tmem255b’, ’Tmem258’, ’Tmem38a’, ’Tmem40’, ’Tmem44’, ’Tmem50a’, ’Tmem55a’, ’Tmem55b’, ’Tmem59’, ’Tmem59l’, ’Tmem64’, ’Tmem65’, ’Tmem88’, ’Tmem88b’, ’Tmem91’, ’Tmem97’, ’Tmem98’, ’Tmod1’, ’Tmod2’, ’Tmod3’, ’Tmsb10’, ’Tmsb4x’, ’Tmtc1’, ’Tmtc4’, ’Tmx1’, ’Tnfaip6’, ’Tnfaip8l1’, ’Tnfaip8l2’, ’Tnfaip8l3’, ’Tnfrsf19’, ’Tnfrsf21’, ’Tnfsf12’, ’Tnik’, ’Tnnc2’, ’Tnnt1’, ’Tnr’, ’Tnrc6a’, ’Tns1’, ’Tns3’, ’Tomm34’, ’Tomm6’, ’Tox’, ’Tox2’, ’Tox3’, ’Tpbg’, ’Tpd52’, ’Tpd52l1’, ’Tpi1’, ’Tpm1’, ’Tpm2’, ’Tpm3’, ’Tpm4’, ’Tppp3’, ’Tpst1’, ’Tpt1’, ’Trabd2b’, ’Traip’, ’Trem2’, ’Trf’, ’Trhde’, ’Trhr’, ’Trib2’, ’Tril’, ’Trim2’, ’Trim59’, ’Trim62’, ’Trim9’, ’Trnp1’, ’Trp53i11’, ’Trp73’, ’Trpc3’, ’Trpc4’, ’Trpc5’, ’Trpc6’, ’Trpm3’, ’Trps1’, ’Tsc22d1’, ’Tsc22d3’, ’Tsc22d4’, ’Tshz1’, ’Tshz2’, ’Tshz3’, ’Tsnax’, ’Tspan13’, ’Tspan15’, ’Tspan17’, ’Tspan18’, ’Tspan2’, ’Tspan3’, ’Tspan6’, ’Tspan7’, ’Tspan9’, ’Tspyl2’, ’Tspyl4’, ’Tst’, ’Ttc3’, ’Ttc39c’, ’Ttc9b’, ’Ttll7’, ’Ttyh1’, ’Ttyh2’, ’Tub’, ’Tuba1a’, ’Tuba1b’, ’Tuba4a’, ’Tuba8’, ’Tubb2a’, ’Tubb2b’, ’Tubb3’, ’Tubb4a’, ’Tubb4b’, ’Tubb5’, ’Tubg2’, ’Tubgcp2’, ’Tunar’, ’Tusc3’, ’Twsg1’, ’Txn1’, ’Txndc5’, ’Txnip’, ’Tyro3’, ’Tyrobp’, ’U2af1’, ’Uap1’, ’Uba52’, ’Ubald1’, ’Ubash3b’, ’Ubb’, ’Ubc’, ’Ube2b’, ’Ube2e2’, ’Ube2e3’, ’Ube2g2’, ’Ube2ql1’, ’Ube3a’, ’Ubl5’, ’Uchl1’, ’Uchl3’, ’Ucp2’, ’Ugdh’, ’Ugp2’, ’Ugt8a’, ’Uhrf1bp1l’, ’Unc119’, ’Unc13b’, ’Unc13c’, ’Unc5a’, ’Unc5c’, ’Unc5d’, ’Unc93b1’, ’Uqcc2’, ’Uqcr10’, ’Uqcr11’, ’Uqcrb’, ’Uqcrh’, ’Uqcrq’, ’Usmg5’, ’Usp2’, ’Usp29’, ’Usp3’, ’Usp46’, ’Usp54’, ’Ust’, ’Utrn’, ’Vamp1’, ’Vamp2’, ’Vamp5’, ’Vamp8’, ’Vapa’, ’Vat1’, ’Vat1l’, ’Vav2’, ’Vbp1’, ’Vcam1’, ’Vcl’, ’Vegfa’, ’Vegfb’, ’Vgf’, ’Vgll3’, ’Vim’, ’Vip’, ’Vmp1’, ’Vopp1’, ’Vps26a’, ’Vrk1’, ’Vsig2’, ’Vsnl1’, ’Vstm2a’, ’Vstm2b’, ’Vstm2l’, ’Vstm4’, ’Vtn’, ’Vwa1’, ’Vwc2’, ’Vwc2l’, ’Wasf1’, ’Wasf2’, ’Wasl’, ’Wbp11’, ’Wdr1’, ’Wdr6’, ’Wfdc1’, ’Wfs1’, ’Whrn’, ’Wipf3’, ’Wls’, ’Wnk1’, ’Wnt10a’, ’Wnt4’, ’Wnt5a’, ’Wnt7b’, ’Wnt9a’, ’Wsb1’, ’Wscd1’, ’Wwc1’, ’Wwox’, ’Wwtr1’, ’Xkr4’, ’Xkr6’, ’Xpnpep1’, ’Xpr1’, ’Xylt1’, ’Ybx1’, ’Ybx3’, ’Ydjc’, ’Yif1b’, ’Ykt6’, ’Ypel1’, ’Ypel2’, ’Ypel3’, ’Ypel5’, ’Ywhab’, ’Ywhae’, ’Ywhah’, ’Ywhaq’, ’Ywhaz’, ’Zbtb16’, ’Zbtb18’, ’Zbtb20’, ’Zc3h7a’, ’Zcchc12’, ’Zcchc18’, ’Zdbf2’, ’Zdhhc12’, ’Zdhhc14’, ’Zdhhc22’, ’Zdhhc23’, ’Zdhhc8’, ’Zeb1’, ’Zeb2’, ’Zfhx3’, ’Zfp189’, ’Zfp207’, ’Zfp296’, ’Zfp36’, ’Zfp365’, ’Zfp36l1’, ’Zfp36l2’, ’Zfp385a’, ’Zfp385b’, ’Zfp385c’, ’Zfp428’, ’Zfp467’, ’Zfp536’, ’Zfp599’, ’Zfp608’, ’Zfp703’, ’Zfp710’, ’Zfp804a’, ’Zfp827’, ’Zfp831’, ’Zfp941’, ’Zfpm2’, ’Zfyve28’, ’Zic1’, ’Zic4’, ’Zmat2’, ’Zmat4’, ’Zmat5’, ’Zwint’, ’Zyg11b’, ’Zyx’]
